# Comprehensive view of microscopic interactions between DNA-coated colloids

**DOI:** 10.1038/s41467-022-29853-w

**Published:** 2022-04-28

**Authors:** Fan Cui, Sophie Marbach, Jeana Aojie Zheng, Miranda Holmes-Cerfon, David J. Pine

**Affiliations:** 1 Department of Physics, New York University, New York, NY USA; 2 Courant Institute of Mathematical Sciences, New York University, New York, NY USA; 3 CNRS, Sorbonne Université, Physicochimie des Electrolytes et Nanosystèmes, Interfaciaux, F-75005 Paris, France; 4 Department of Chemical & Biomolecular Engineering, New York University, New York, NY USA

**Keywords:** Colloids, Organizing materials with DNA, Polymers

## Abstract

The self-assembly of DNA-coated colloids into highly-ordered structures offers great promise for advanced optical materials. However, control of disorder, defects, melting, and crystal growth is hindered by the lack of a microscopic understanding of DNA-mediated colloidal interactions. Here we use total internal reflection microscopy to measure in situ the interaction potential between DNA-coated colloids with nanometer resolution and the macroscopic melting behavior. The range and strength of the interaction are measured and linked to key material design parameters, including DNA sequence, polymer length, grafting density, and complementary fraction. We present a first-principles model that screens and combines existing theories into one coherent framework and quantitatively reproduces our experimental data without fitting parameters over a wide range of DNA ligand designs. Our theory identifies a subtle competition between DNA binding and steric repulsion and accurately predicts adhesion and melting at a molecular level. Combining experimental and theoretical results, our work provides a quantitative and predictive approach for guiding material design with DNA-nanotechnology and can be further extended to a diversity of colloidal and biological systems.

## Abstract (web summary)

A quantitative prediction of DNA-mediated interactions between colloids is crucial to the design of colloidal structures for optical applications. Cui et al. measure the interaction potential with nanometer resolution and propose a theory to accurately predict adhesion and melting at a molecular level.

## Introduction

DNA-coated colloids are our most versatile tool for the targeted self-assembly of colloidal materials^1–6^, which have important applications in photonics^7–9^, metamaterials^10,11^, and biomedical devices^12,13^. Their versatility stems from the programmability of their single-stranded DNA (ssDNA) sticky ends, a sequence of nucleobases that enable specific attractive binding to particles coated with complementary ssDNA^14–20^. While programming the sticky ends determines which DNA ligands on different particles bind, control of collective processes, such as the binding-unbinding transition, commonly referred to as melting, and crystal growth and size, is hindered by our limited understanding of the microscopic mechanisms at play^21–23^. Recent experimental work, for example, has highlighted the importance of the areal density of the DNA ligands for controlling binding kinetics and optimizing crystallization^24–26^. Moreover, with the advent of patchy particles^27,28^ and colloids with complex non-spherical shapes^19,29,30^, controlling the range of the interaction by adjusting the lengths of the ligands becomes increasingly important. Predicting how all these parameters interact to control the binding and unbinding of DNA-coated particles represents a formidable challenge, which we address in this paper.

Directly probing microscopic DNA-mediated interactions is an indisputable challenge^31–33^. This is due in part to the zoology of forces beyond DNA ligand binding occurring at these scales, including steric repulsion^34^, van der Waals^35^, and electrostatics^36,37^. Early foundational work highlighted the crucial role of entropy during binding, which is related to ligand conformations^31,38^ and the competition between binding partners^21,23,32,33,39,40^. However, limited investigation of different experimental designs makes it hard to pinpoint and control the interaction mechanism. In particular, at high ligand coverage, which promotes fast kinetics^6,26,41–43^, no direct comparison between experimental potential profiles and theoretical predictions exists. Standard modeling based on a discrete numerical account of ligands is intractable at high coverage due to the high number of ligands^31,33^. Finally, simultaneous measurement of microscopic interactions and macroscopic material properties remains uncharted.

In this paper, we directly measure the interaction potential between high-density DNA-coated colloids using total internal reflection microscopy (TIRM). We present first-principles modeling that reproduces experimental data over a wide range of ssDNA ligand designs without fitting parameters. Experiments and theory highlight the crucial role of steric repulsion between brushes, balancing attractive binding forces over a short distance to form an extremely narrow potential well. We demonstrate quantitative tuning of experimental systems to achieve high control over macroscopic material properties such as the melting temperature. Finally, we unveil a microscopic view of binding, where surfaces start interacting with about a dozen bonds at the melting temperature, then strongly compress the polymer brush by up to 20 nm upon cooling.

## Results and discussion

### Experiments

**Fig. 1:**
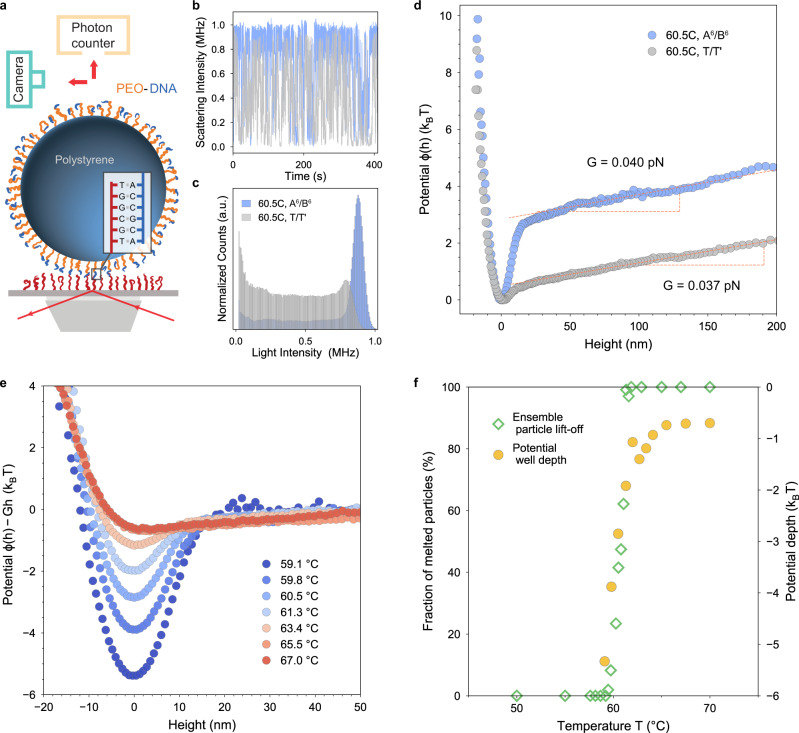
Measurement of potential between DNA-coated surfaces using total internal reflection microscopy. **a** Schematic of the experimental setup. A DNA-coated PS particle is illuminated by an evanescent wave generated by a 633 nm laser. The scattered light is detected by a PMT photon counter and simultaneously with a camera to track particle positions. The glass substrate surface is coated with the complementary DNA strands, here with 6 sticky bases. Drawings are not to scale. **b** Scattering intensity, **c** corresponding statistical histogram, and **d** potential energy profile of a DNA-coated particle measured at 60.5 °C. **b**–**d** Particle/substrate pairs with DNA strands with six sticky bases, A^6^/B^6^, (respectively with non-interacting bases, T/T${}^{\prime}$) are blue (resp. gray). **e** Potential energy profiles, with gravity removed, of a DNA-coated particle (A^6^/B^6^) at different temperatures. **f** Potential well depth vs. temperature (yellow circles) with a superimposed melting curve, based on ~200 particles (green diamonds) of DNA-coated particles (A^6^/B^6^). All particles measured have a diameter of 5 μm and PEO linkers with a molecular weight 34 kg/mol clicked to 20-base DNA strands. The glass is coated with 60-base DNA strands. Measurement solution consists of 140 mM PBS (pH = 7.4) and 0.3% F127. Refer to Table 1 for full DNA sequences.

We probe the interaction potential between DNA-coated surfaces using a custom-built, highly sensitive TIRM (Fig. 1a). Both surfaces of the polystyrene (PS) particle and the glass substrate are coated with ssDNA (Methods)^44,45^. On the PS particle, the DNA strands are anchored onto the surface through a polyethelyne oxide (PEO) linker with variable molecular weight *M*_*w*_. The brush-mediated DNA functionalization results in a high-density DNA coating with densities ranging from 0.1 to 0.02 nm^−2^ (Supplementary Discussion Section 2.2). Interactions between the two surfaces are modulated both by varying *M*_*w*_ of the PEO brush and the binding strength of the ssDNA sticky ends (Table 1).

**Table 1:** Sequence of the DNA strands used in this work.

Strands	Sequence
*DNA on colloids*	
Complementary A^6^	${5}^{\prime}$-/DBCO/-T_14_-**ACCGCA**-$3^{\prime}$
Complementary A^5^	$5^{\prime}$-/DBCO/-T_15_-**GACGC**-$3^{\prime}$
Complementary A^4^	$5^{\prime}$-/DBCO/-T_16_-**GCAG**-$3^{\prime}$
Non-interacting T	$5^{\prime}$-/DBCO/-T_20_-$3^{\prime}$
*DNA on glass surfaces*	
Complementary B^6^	$5^{\prime}$-/DBCO/-T_54_-**TGCGGT**-$3^{\prime}$
Complementary B^5^	$5^{\prime}$-/DBCO/-T_55_-**GCGTC**-$3^{\prime}$
Complementary B^4^	$5^{\prime}$-/DBCO/-T_56_-**CTGC**-$3^{\prime}$
Non-interacting T$^{\prime}$	$5^{\prime}$-/DBCO/-T_60_-$3^{\prime}$

DNA sequences in bold font indicate complementary base pairs.

To measure the interaction potential using TIRM, a particle is illuminated with an exponentially damped evanescent wave. As the particle travels vertically, the intensity of the light scattered by the particle, which decays exponentially with particle height^46,47^, is measured with a photomultiplier (PMT) photon counter (Fig. 1b). The statistical distribution of the scattered intensities (Fig. 1c) is therefore a measure of height distributions *p*(*h*), which is related to the potential *ϕ*(*h*) by Boltzmann’s equation:

1 $$p(h)=\frac{1}{Z}\exp \left[-\frac{\phi (h)}{{k}_{B}T}\right],$$

where *k*_*B*_ is Boltzmann’s constant, *T* the temperature, and *Z* is a normalization factor. When the number of observations is large (more than 350,000 in our experiments), *ϕ*(*h*) can be reliably inferred from light intensity distributions (Methods and Supplementary Discussion Section 1).

We employ the TIRM technique first to investigate colloid/substrate coatings with complementary (A^6^/B^6^, six sticky bases) and non-interacting (T/T${}^{\prime}$) DNA strands (Fig. 1a–c). For a sample with ssDNA sticky ends, the scattered intensities (blue trace in Fig. 1) exhibit long intervals of high scattered intensity and relatively short intervals of low intensity, indicating that the particle spends most of its time-bound to the surface and occasionally breaks bonds and diffuses away from the surface. TIRM analysis of this signal, shown in Fig. 1d, shows that the sticky particle exhibits a sharp attractive potential well with a depth ≃ 2.8*k*_*B*_*T* at small separation distances. By contrast, a particle with non-interacting strands spends much more time away from the surface and exhibits a very shallow well with a depth of < 0.5*k*_*B*_*T*. The sharp attractive potential well for the particle with complementary strands can thus be attributed to DNA hybridization interactions. The width of the well is artificially broadened by photon counting (see Methods). At larger separations beyond 50 nm, both potentials show a linear upward increase, consistent with the 0.037 pN gravitational force expected for our 5-μm diameter PS particles.

The temperature sensitivity of DNA hybridization is key for macroscopic assemblies. Figure 1e shows the potential profiles of a single particle with six sticky bases over an 8 °C temperature range. Here, the gravitational contribution is subtracted to emphasize surface interactions (Methods). The attractive well becomes shallower as the temperature is increased, indicating fewer hybridization bonds. Figure 1f shows that the potential well depth decreases from 5.5*k*_*B*_*T* to 0.8*k*_*B*_*T* between 59 °C and 64 °C. As temperature increases above 64 °C, the well depth plateaus around 0.8 *k*_*B*_*T*. Interestingly, we still observe a non-zero attractive interaction potential at high temperatures when DNA hybridization should be negligible. This potential well is also present in the non-interacting particle potential (Fig. 1d). This range and strength of the attraction are consistent with van der Waals interactions (Supplementary Discussion Section 3.5).

We can relate the well depth, a microscopic single-particle property, to the melting of the DNA-coated particles, an important macroscopic material property. The melting of DNA-coated colloids aggregates is usually characterized by the fraction of unpaired particles (singlets) as a function of temperature^43,48^. Here, we adopt a similar definition and plot the fraction of “melted” particles as a function of temperature. In the particle-substrate geometry, we take a particle as melted when it has lifted off from the surface, at least once 20 nm beyond the potential minimum during a 1-minute observation window (Supplementary Fig. S2). This method directly captures unbinding by measuring particle separation, in contrast with some other work^49^ that infers melting by tracking lateral particle motion. We observe ~200 DNA-coated particles using the camera on TIRM and plot the percentage of melted particles as a function of temperature in Fig. 1f. The melting curve shows a sharp transition with *T*_*m*_ ≃ 60.5 °C, which coincides with the potential well depth of roughly 3*k*_*B*_*T*. At potential depths above 1.5*k*_*B*_*T*, the particles are completely melted. Figure 1f shows a clear correspondence between microscopic interaction energy and macroscopic ensemble melting.

### Multiscale model

To understand how microscopic material design affects macroscopic melting, we build a predictive model. A careful account of the polymer brush, especially of entropic costs due to loss of degrees of freedom upon binding, is central for quantitative description^6,21,38–40^. Previous modeling approaches relied either on Monte Carlo simulations to infer the configurations of ligands^31,33^ or on approximate geometrical variables used as fitting parameters^22,38^. Here, our aim is to make quantitative predictions over a wide range of ligand designs. We, therefore, avoid loosely-defined variables and fitting and rely on a mean-field description to account for the detailed geometry of our brushes.

**Fig. 2:**
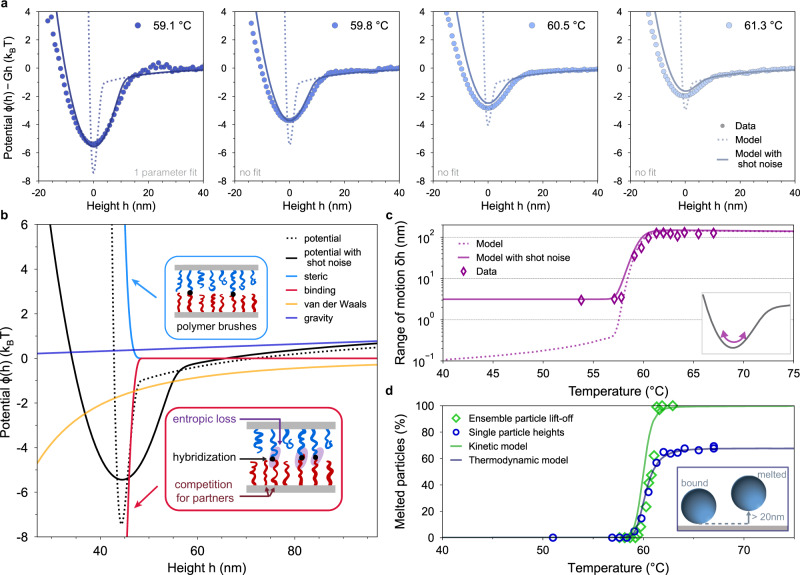
Sticky polymer brush model reproduces experimental data. **a** Model and experimental potential profiles of the DNA-coated particle are presented in Fig. 1d with a measured coating density *σ* = 0.021 nm^−2^ of 34 k PEO with six sticky bases. The potential is shown at four temperatures around *T*_*m*_ = 60.5 °C. The density of strands on the glass (*σ*_*g*_ = 0.011 nm^−2^) is the only fitting parameter and is fitted only once at 59.1 °C. No additional fitting is done for higher temperatures. Shot-noise consistent with experimental measurements is applied to the model predictions. **b**, Contributions to the model potential profile at 59.1 °C. (Insets) Schematics of components accounted for in the model. **c** Range of motion $\delta h={\langle {h}^{2}\rangle }_{\phi }^{1/2}$ around the potential minimum, (inset) describing the typical displacement of the particle in the well (Supplementary Discussion Section 3.2.1). **d** Experimental and model thermodynamic and kinetic melting curves. The experimental kinetic curve corresponds to the ensemble particle lift-off also reported in Fig. 1e). To compute the kinetic melting curve we use the hindered diffusion of the free particle at height *h* ≃ 0.3 nm above the hydrodynamic floor, *D*_⊥_ ≃ *D*_0_(*h*/*a*)^59^ where *D*_0_ = 2.09 × 10^−13^ m^2^/s is the measured bare diffusion coefficient and 2*a* = 5 μm is the diameter of the particle. Both methods are insensitive to measurement noise (Supplementary Discussion Section 4.2). (Inset) The thermodynamic method measures the fraction of particles lifted at least 20 nm above their equilibrium position.

A series of calibration experiments^50^ reveals PEO coatings that are thick, 12–40 nm, and dense, 3–7 nm between grafts, (Supplementary Discussion Section 2), which validates the use of a model based on the Milner-Witten-Cates (MWC) mean-field brush model^34,51,52^. We extend the MWC theory to our more complex and dense brushes. Notably, our calculation of the steric repulsion *ϕ*_steric_(*h*) includes the effects of having a fraction *f* of bound ends^53^ which modifies the brush’s structure due to excluded volume interactions between adjacent strands. Further, we account for the heterogeneous composition of the tether (DNA–PEO), and the different DNA coating densities on the particles and the substrate^54^. The binding attraction *ϕ*_binding_(*h*) includes the free energy of hybridization of Δ*G*^(0)^ of the complementary DNA strands used^55^. Competition for binding partners and entropic contributions due to the loss of degrees of freedom upon binding are included consistently within our brush model^21,39,40^. The fraction of bound ends *f* is then determined by minimizing *ϕ*_binding_(*h*) + *ϕ*_steric_(*h*) (Supplementary Discussion Section 3.4). Adding gravity and van der Waals attraction, the potential *ϕ*(*h*) = *ϕ*_grav_ + *ϕ*_binding_ + *ϕ*_steric_ + *ϕ*_vdw_, is shown by the dotted black line in Fig. 2b. The experimental shot noise, which cannot be eliminated, can be quantitatively controlled to a known level^56^. Using an analytic noise convolution kernel, we calculate the noise-broadened potential curve with the known Poisson distribution, shown by the solid black curve in Fig. 2b (Methods and Supplementary Discussion Section 4).

Below the melting temperature, competition between the binding attraction (red) and steric repulsion (blue) forms a narrow potential well, barely 2-nm wide. Both contributions occur at the same separation: when brushes touch, binding (attraction) is favored as well as compression (repulsion). The balance of these two forces is therefore subtle, and only observed around and below the melting temperature when the hybridization energy Δ*G*^(0)^(*T*) is sufficiently favorable. Importantly, our predictions of narrow potentials are well represented by new short-range Lennard–Jones potentials^57^ (and not by, e.g., Morse, Supplementary Discussion Section 6), validating their applicability for self-assembly simulations^32,58^.

### Comparison of model and data

We use this model to compare the predicted potential profiles *ϕ*(*h*) to the experimentally measured ones. Input parameters for the model, including temperature, brush length, DNA density, and target photon-counting *N*_photons_ are all taken directly from measured values. The hybridization energies of sticky ends are determined from tabulated values using the second nearest neighbor model of SantaLucia^55^. The only parameter that is not precisely known is the glass coating density *σ*_*g*_, which is determined by fitting the potential to data obtained at a single temperature, 59.1 °C, for a single particle type; the data and fit are shown in Fig. 2a (first panel). We obtain *σ*_*g*_ = 0.011 nm^−2^, consistent with the expected range (Supplementary Discussion Section 2.2), and fix it at this value for all remaining predictions. All the parameters entering the model are known with limited uncertainty and kept the same for the different systems explored (Supplementary Table S12). No additional fitting or noise blurring is done on our calculations.

Figure 2a (remaining three panels) compares the measured potentials with model predictions at three different temperatures. The predicted potentials are in remarkable agreement with experiments, including the temperature-dependence of the noise-adjusted widths and depths of the potential, as well as the overall shapes. Interestingly, while the measured potential width is ~10 nm (defined as the full width at ≃ 1*k*_*B*_*T* above minimum) at 59.1 °C, our model indicates that it is broadened by shot-noise from a potential ~2 nm wide. Such narrow widths are consistent with separate investigations of the effects of photon counting shot noise on TIRM measurements^56^.

Particle unbinding can be directly probed from potential measurements by plotting the root-mean-square height fluctuations $\delta h=\sqrt{{\langle {h}^{2}\rangle }_{\phi }}$, around the potential minimum (see Figure 2c.) The rms height *δh* quantifies the range of motion of the particle and can be calculated from both our TIRM measurements and our model. For temperatures below 57 °C, the particle is tightly confined within the potential well with a predicted range of motion *δh* ≲ 0.4 nm; the TIRM measurements saturate at *δh* = 3 nm, a value set by the photon counting shot noise (Methods and ref. ^56^). Clear evidence of particle unbinding is seen at 58 °C and above, where *δh* increases dramatically over a temperature window a few degrees wide. At temperatures above 62 °C, *δh* reaches a plateau set by the gravitational height of the particle, which is 124 nm (Supplementary Discussion Section 3.2.1).

Macroscopic quantities such as the melting temperature *T*_*m*_ can also be modeled. From a modeling perspective, the “lifting-off” criteria for the “melted” particles discussed earlier is essentially a kinetic definition and it corresponds to a mean first passage time problem for the particles to lift-off beyond the potential well^60^ (Supplementary Discussion Section 3.2). The kinetic melting curve depends on the vertical diffusion coefficient of the particle. Here we approximate the diffusion coefficient by a representative value corresponding to hindered hydrodynamic diffusion near a wall^61^ and find excellent agreement with the experimental measurement (see Fig. 2d).

To provide another perspective on melting, we introduce a single-particle, thermodynamic melting definition, where the fraction of melted particles corresponds to the fraction of time the particle remains unbound at heights *h* beyond the attractive binding well (*h* ≳ 20 nm):

2 $${p}_{{{{{{{{\rm{unbound}}}}}}}}}=1-\frac{1}{Z}\int\nolimits_{0}^{{h}_{c}}\exp \left[-\frac{\phi (h)}{{k}_{B}T}\right]dh.$$

**Fig. 3:**
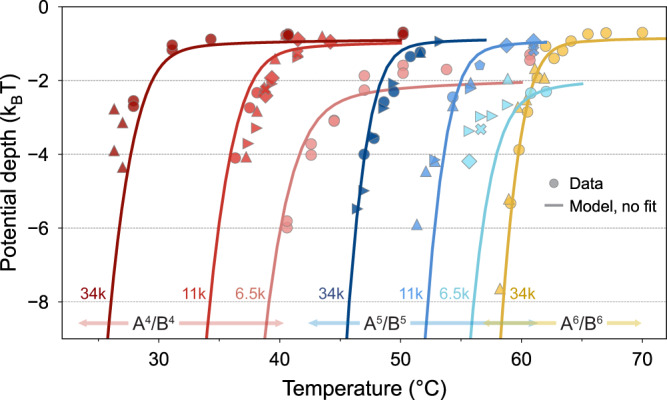
Control of melting properties with hybridizing pair number and chain length. Potential well depths measured for different experimental systems: PEO brushes with *M*_*w*_ of 6.5 k, 11 k, and 34 k, and colloid/glass surfaces attached with 4 (-GCAG/-CTGC), 5 (-GACGC/-GCGTC) or 6 (-TGCGGT/-ACCGCA) DNA complementary bases. The different symbols correspond to different particles. The model predictions (with no additional fitting) with photon shot noise are overlaid. The particles with 34 k and six sticky bases correspond to that in Fig. 1e, f, and Fig. 2. At high temperatures, all particles experience a constant ~1–2 *k*_*B*_*T* well depth from van der Waals interaction.

This definition, though on a single-particle level, captures the same thermodynamic melting picture drawn from a large ensemble of particles that rely on statistics to determine the fraction of bound particles. The single-particle melting curve is obtained from the raw scattering data (*e.g*. Fig 1b) that traces the particle position during the one measurement and is shown in Fig. 3c (blue circles). The model prediction (line), shows excellent agreement with experiments. Note that *p*_unbound_ does not reach 100% at high temperatures due to gravity. Remarkably, the kinetic and thermodynamic melting curves transition at the same temperature *T*_*m*_ = 60.5 °C. This sheds light on the melting dynamics: DNA pairs unbind, and a particle lifts-off with an escape rate of the order of 1 min (Supplementary Discussion Section 3.2.2).

### Control of macroscopic melting

Our model indicates that the key to controlling the melting point is to adjust the balance between hybridization and steric repulsion. Hybridization is determined by the type and number of the sticky bases, while steric repulsion is determined by the length and density of the underlying PEO polymer layer. To test our model, we experimentally measured the potential well depth of colloids with different numbers of complementary DNA sticky bases (4, 5, or 6) and PEO molecular weights (*M*_*w*_ = 6.5, 11, and 34 kg/mol). For each configuration, multiple particles are measured to ensure the reproducibility and homogeneity of our measurements. The experimental, as well as the predicted well depth results, are shown in Fig. 3.

First, we find that our theoretical predictions are in close agreement with the well depths measured experimentally with TIRM for a wide variety of colloid coatings. The robust agreement, spanning a broad range of temperatures, indicates that our model is able to successfully capture the detailed molecular balance that determines macroscopic melting. Moreover, as shown in Supplementary Discussion Section 5.2, the agreement is robust within the uncertainties of the experimental parameters.

Second, we find that changing the binding attraction by reducing the number of sticky bases from 6 (yellow) to 5 (blue) to 4 (red) in Fig. 3 consistently depresses the melting point by 10 to 30 °C for each PEO length. Importantly, while the bare free energies of the 4, 5, and 6 sticky bases predict melting temperatures separated, respectively, by 10 °C and 12 °C^55^, our model reproduces the nonlinear features observed where the relative spacing of the melting curves is not preserved due to the different brush lengths.

Third, we observe that for a given number of sticky bases, decreasing the chain length from *M*_*w*_ = 34 kg/mol to 6.5 kg/mol consistently increases the melting point by up to 15 °C. Separate model inquiries (Supplementary Discussion Section 5.2.3) show that this originates equally from two contributions. Decreasing chain length decreases the number of polymer strands whose degrees of freedom are reduced by compression of the brush. This reduces the entropic penalty of compressing the brush, which decreases steric repulsion and promotes binding. Moreover, experimentally we find that shorter chain lengths are associated with higher areal densities. Our model shows that increased areal density increases binding attraction more than it increases steric repulsion, which is a useful and interesting insight (Supplementary Discussion Section 5.2.3).

The faithful reproduction of experimental data support its use as a predictive tool for the rapid exploration of different material designs (To enhance accessibility, our model is available through GitHub: https://github.com/smarbach/DNACoatedColloidsInteractions). In fact, we also find close agreement of melting temperature predictions with previously reported experimental data of different material systems by other researchers^31,49^, including at lower coating densities (Supplementary Discussion Section 7). Additionally, our mechanistic knowledge suggests new design avenues. For example, two different DNA sticky sequences could melt at the same temperature (as is nearly obtained here for 5 bases, *M*_*w*_ = 6.5 kg/mol and 6 bases, *M*_*w*_ = 34 kg/mol) or even at inverted temperatures, by tuning the length of the polymer chain, thus permitting a change in the range of interaction without necessarily changing its strength.

### Melting and binding

Our TIRM measurements uncover a detailed microscopic picture of DNA-mediated melting and binding below the melting temperature. By monitoring the separation between binding partners at the nanometer scale, TIRM provides detailed information about binding beyond the 2-degree melting transition window probed by macroscopic melting curves as it also measures how the polymer brush is compressed when the temperature is lowered far below the melting temperature.

**Fig. 4:**
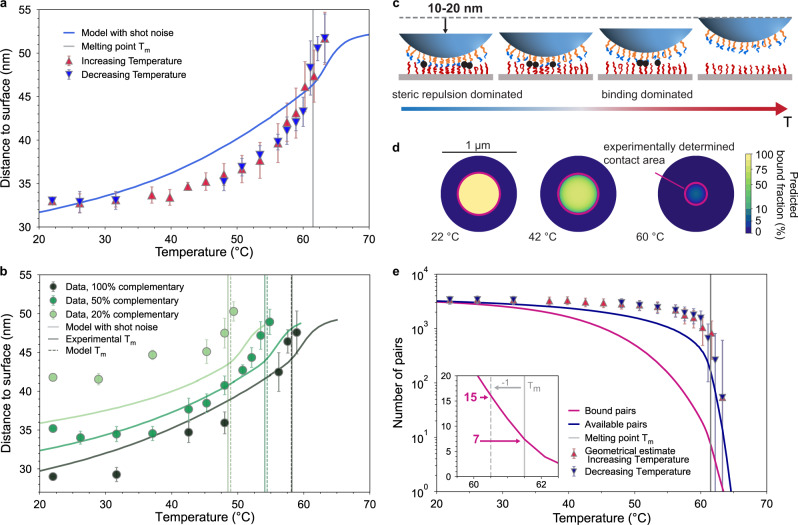
Microscopic insight into the melting transition. **a** Height *h* of potential minimum for a 34 k PEO + A^6^/B^6^ particle, measured with TIRM for increasing and decreasing temperatures and model predictions. For this system *T*_*m*_ = 61.5 °C. **b** Height of particles with varying complementary coverage fraction *x*, with 34 k PEO + A^6^/B^6^ (*x*%) + T/T${}^{\prime}$ (100 − *x*%), as measured with TIRM for decreasing temperatures. *T*_*m*_ = 59.2 °C for the 100% complementary. In the model, a particle density *σ*_*p*_ = 0.012 nm^−2^ (within the measured density variability) was used to yield the same *T*_*m*_ as measured for 100%. No additional fitting is done for the 20 or 50%. Error bars in **a**, **b** correspond to the standard deviation of five particles that are measured at each temperature. **c**, Sketch of microscopic melting from low to high temperatures. Bonds (which fluctuate in time) are highlighted with black dots. **d** Contact area with radius $r\simeq 2a({h}_{\max }-h)$ where *h* is the separation distance from **a** and ${h}_{\max }\simeq 52\,{{{{{{{\rm{nm}}}}}}}}$ the maximal interaction height. Here 2*a* = 5 μm. Overlapped model predictions show the proportion of bound pairs over the particle’s bottom surface. **e** Number of pairs within range (available) for binding calculated from experimental data of **a** as *πr*^2^*σ*_*g*_ with *r* from **d**. Error bars correspond to 6% uncertainty on ${h}_{\max }$. Model prediction for the average number of available and bound pairs and (inset) zoom around *T*_*m*_ (Supplementary Discussion Section 3.7). Model parameters for **d**, **e** correspond to those of **a**.

Figure 4a shows TIRM measurements of the height *h* of the potential minimum from 6 °C above to 40 °C below *T*_*m*_ for a 34 k PEO + A^6^/B^6^ particle-substrate interaction. The absolute values of separation distances are acquired using a calibration procedure described in Supplementary Discussion Section 1.3. The measurements reveal a two-stage binding process. Within a few degrees above and below *T*_*m*_, *h* decreases rapidly as the particle binds and the polymer brushes are compressed 10 nm from ~52 to 42 nm over a 4 °C temperature window. Within this window, the particle dynamically binds and unbinds, spending an increasing fraction of time-bound towards the lower end of this temperature range, until the potential depth reaches about 8*k*_*B*_*T*. Below this range, in the second stage of binding, the particle remains bound at all times and its vertical excursions are increasingly confined to a narrow sub-nanometer range around its mean position, as seen in Fig. 2c. However, in this second stage, the polymer brush continues to be compressed by nearly another 10 nm as the temperature is lowered until the height reaches a plateau value of about 33 nm at *T* ≃ 40 °C. This corresponds to a coefficient of linear thermal expansion on the order of 400 × 10^−6^ K^−1^, 10–100 times greater than metals, and several times larger than most rubbers. No hysteresis is apparent as the temperature is lowered and raised, suggesting thermal equilibrium.

Model predictions show a similar quantitative trend with a 20-nm compression of the brush, but the transition is more gradual with less of a distinction between the two stages of binding and compression. We attribute the discrepancy to the presence of an adsorbed short copolymer (Pluronic F127, Methods) within the brush, which makes the brush softer towards its edge (Supplementary Discussion Section 2.3).

We further investigate control of melting and binding by altering the relative strengths of attraction and repulsion. To do so, we vary the fraction of polymer strands that have DNA sticky ends from 100% to 50% and 20%, which decreases binding attraction without affecting steric repulsion. As shown in Fig. 4b, all particles exhibit a two-stage height change with temperature, as we observed previously, including a sharp compression near melting followed by a more gradual compression toward an ultimate plateau at low temperatures. Our model correctly predicts the melting temperature in all cases, but overestimates the compression of the brush for the case of 20% DNA sticky-end fraction. This may be due to a breakdown of the mean-field approximation as the fluctuations in the areal density of DNA sticky ends are significant in this case, meaning that some sticky ends may not be able to find a binding partner at low temperatures.

The observed compression of the polymer brush provides useful insights into the melting process. Far below the melting temperature where the particle height is ~33 nm (Fig. 4a), the brushes from the two surfaces are in contact over an area with a diameter of ~500 nm (Fig. 4d). Of the 3000 sticky-end pairs present in this area, all are hybridized at low temperatures (Fig. 4e), thus allowing new bonds only at the periphery. As the temperature is increased, the area and number of sticky ends available for binding decreases; the fraction of available bonds that actually bind also decreases, as shown in Fig. 4e. At *T*_*m*_, our model predicts seven bound pairs out of ~300 pairs that are available for binding, which stabilizes the interaction.

### Outlook

As particle designs and directed colloidal self-assembly targets become more ambitious, the need to understand and control the interaction range, strength, and cooperativity of DNA brushes tethered to particle surfaces becomes a pressing problem. Here we provide a comprehensive understanding of DNA-mediated particle binding, which is directly probed by TIRM experiments and analytically described by a detailed account of microscopic forces, wrapped in a mean-field, rapidly-computed polymer model. The results allow one to quantitatively predict how ligand length, density, and binding strength interact cooperatively to control melting and binding behaviors. The cooperativity of these interactions makes them highly nonlinear, as illustrated by how increasing the areal density of ligands increases both the strength of DNA binding and steric repulsion, but with binding attraction winning due to nonlinearities. The quantitative accuracy of our model means it can be used as a design tool to engineer colloidal interactions at the nanoscale. The physical picture also provides insights into the dynamics of binding, which are important for annealing and controlling the diffusion of DNA-bound colloids. Thus, the combined experimental and theoretical approach can serve as a platform for understanding and controlling the dynamics of DNA-mediated binding, an important frontier for the self-assembly of DNA-coated colloids^49,62,63^. For example, exotic designs, with multiple DNA sequences and chain lengths on a single particle to enable binding with different strengths to other particles^64^, are a natural, albeit challenging, the sequel. Finally, our experimental and theoretical assay could serve as a stepping stone to understanding the dynamics of other multivalent ligand-receptor systems, such as virus motion and stalling on mucus through adhesive proteins^65,66^.

## Methods

### Chemicals

All chemicals are purchased from Sigma-Aldrich unless otherwise specified and used as received.

### Preparation of DNA-coated PS colloids

We synthesize DNA-coated PS spheres using a previously reported polymer brush approach with minor modifications^44^. PS-b-poly(ethylene oxide) copolymer (PS-b-PEO) are first functionalized with azide at the end of the PEO chain (PS-b-PEO-N_3_)^67^. Polymers with three different molecular weights (*M*_*w*_) are studied:

PS_3.8*k*_PEO_6.5*k*_, *M*_*w*_ = 11, 124 g/mol, PS = 3800 g/mol, and PEO = 6,500 g/mol;

PS_3.2*k*_PEO_11*k*_, *M*_*w*_ = 15, 336 g/mol, PS = 3200 g/mol, and PEO = 11,000 g/mol;

PS_3.8*k*_PEO_34*k*_, *M*_*w*_ = 39, 690 g/mol, PS = 3800 g/mol, and PEO = 34,000 g/mol.

PS-b-PEO-N_3_ are then attached to PS particles using the swelling/deswelling method^44^. Typically, 250 μL aqueous solution containing 0.005 g/ml particles and 0.5 μM PS-b-PEO-N_3_ is mixed with 160 μL tetrahydrofuran (THF) at room temperature. The mixture is placed on a horizontal shaker (1000 rpm) for 1.5 hours to fully swell the PS particles and absorb the PS block of the PS-b-PEO-N_3_ molecules. Then THF is slowly removed from the solution via evaporation, leaving the hydrophobic PS blocks inserted into the particles and the hydrophilic PEO chains extending out into the solution. The particles are washed with de-ionized water three times to remove excess polymers.

ssDNA (20 bases, purchased from Integrated DNA Technologies with ${5}^{\prime}$ dibenzocyclooctyne (DBCO) end modification, is clicked onto PEO tips through strain-promoted alkyne-azide cycloaddition (SPAAC)^44^. Briefly, PS particles (0.0025 g/ml) previously coated with PS-b-PEO-N_3_ polymer brush are dispersed in 400 μL 500 mM PBS buffer, pH = 7.4. Then 10 μL of DBCO-DNA (0.1 mM) are added to the suspension. The mixture is left to react for 48 hours on a horizontal shaker (1000 rpm). The final product is washed in DI water three times and stored in 140 mM phosphate-buffered saline solution (PBS, pH = 7.4) at 4 °C. The DNA coverage density is measured using flow cytometry (Supplementary Discussion Section 2.2). During TIRM measurements, the colloids are further diluted in 140 mM PBS buffer with 0.3% F127 added as surfactants.

### DNA coating on glass substrate

Polished glass slides (25mm × 75mm, purchased from Delta Technologies) with indium tin oxide (ITO) coated on one side, are first cleaned by sonication (acetone and isopropanol, IPA) and oxygen plasma. The cleaned glass slides are then immersed in a toluene solution containing 11-azidoundecyltrimethoxysilane (N_3_-DTMOS, 2% v/v) and 0.03 M N,N-diisopropylethylamine for 48 hours. The silanization of the glass surface is carried out in an N_2_ filled glovebox to avoid moisture-induced uneven polymerization and multi-layer formation^45^. The substrates are then rinsed with toluene, acetone, and finally, IPA before annealing at 120 °C for 30 min in the glovebox.

We then define the DNA coverage area on an azide-functionalized glass substrate (the glass side) using a silicon reaction cell (22 mm × 22 mm × 0.6 mm). 2.5 μM DBCO modified ssDNA (60 bases) dissolved in 500 mM PBS (pH = 7.4) solution are injected into the cell to react with the N_3_-DTMOS on the glass surface. The reaction is carried out with gentle horizontal shaking (200 rpm) for 48 hours. Finally, the substrate is rinsed in DI water for 10 min to remove the excess DNA before drying with a stream of N_2_.

### TIRM design and data acquisition

We use a TIRM to measure the potential energy of a DNA-coated PS sphere near a glass surface. The microscope is designed and custom built in our lab. A detailed optical train is included in Supplementary Fig. S1. Light from a linearly polarized 632.8-nm laser (HeNe, 30 mW, Lumentum) is directed toward the glass-water interface at 70^∘^ incident angle to generate an evanescent field. The laser is coupled to the measuring cell using an asymmetric dove-shaped glass prism (63^∘^/70^∘^, H-K9L, Tower Optical Corporation), with the 70^∘^ side surface facing the incident beam and the top surface in contact with glass slide through immersion oil.

The scattered light is collected using a 50 × long working distance objective (Mitutoyo Apo Plan 50×, 0.55NA, 13 mm WD). A plate beamsplitter (50:50, Thorlabs) is put in the collection path to split the collected light into two directions, with one going into a photomultiplier tube (PMT) photon counter (Hamamatsu, H7421-40) and the other forming images on a CMOS camera (AMScope, MU1803). We use a National Instruments counter (USB-6341) to output the received photon frequencies. For frequency counting, we use the “target photon number” method in which the counter counts up to a certain number of photons to calculate a frequency. The target photon number is typically set to be 1000.

### Potential profiles from light intensity scattering with TIRM

When a particle is illuminated by an evanescent field, its scatting intensity *I* at a certain height *h* follows the exponential relationship:

3 $$I(h)={I}_{0}\exp (-\alpha h),$$

where *I*_0_ is the scattering intensity when *h* = 0 and *α*^−1^ is the penetration depth of the evanescent field. In this work, all measurements are done with *α*^−1^ = 99 nm.

During a TIRM measurement, a colloidal particle is settled close to the glass substrate and scatters light as it moves vertically via Brownian motion. At equilibrium, the probability that the particle is at a height *h* above the substrate is given by the Boltzmann distribution, Eq. (1). We typically set the lowest measured potential energy to be zero and the corresponding height to be *h*_*m*_. Then the potential energy can be written as $\phi (h)/{k}_{B}T=\ln [p(h)/p({h}_{m})]$. We infer *p*(*h*) from the histogram statistic of the time-dependent scattering. When the number of observation is large, which is typically more than 350,000 in our experiment, *p*(*h*)/*p*(*h*_*m*_) ≃ *n*(*h*)/*n*(*h*_*m*_) where *n*(*h*) is the number of times the particle stays in the vicinity of height *h*. The probability of the particle being at a height *h* is equal to the probability of measuring the *h*-corresponding intensity *I*: *p*(*I*) ∣*dI*∣ = *p*(*h*) ∣*dh*∣, where *p*(*I*) is the probability density of the light intensities observed. With Eq. (3), *p*(*h*) = *p*(*I*) *αI*.

Hence, the potential energy can be written as:

4 $$\frac{\phi (h)-\phi ({h}_{m})}{{k}_{B}T}=\ln \left[\frac{n({I}_{m})\,{I}_{m}}{n(I)\,I}\right].$$

Where *I* is the scattering intensity when the particle is at height *h*, *n*(*I*) is the number of observations of intensity in the range from *I* to *I* + *δI*. We refer the lowest potential energy *ϕ*(*h*_*m*_) to be 0 and the corresponding height and scattering intensity to be *h*_*m*_ and *I*_*m*_, respectively.

### Gravity removal and well depth determination

To remove the gravitational contribution *Gh* from the potential, we first acquire the gravity through linear regression of the potential curve in the large-separation, linearly increasing region (usually 80–200 nm above the potential minimum). The fitted slope Δ*ϕ*/Δ*h* = *G*. For potential curves originating from the same particle, we fit gravity and obtain a value of *G* for each potential profile. Then take the average value of *G* to remove gravity from all potential curves for this specific particle.

Potential well depths are calculated from potentials with gravity removed. We apply parabola fitting around the potential minimum within the potential well and compute the fitted minimum as the potential well depth. Parabola fitting range is usually done up to *h*_*m*_ ± 4 nm.

### Shot-noise-limited resolution from photon counting

In all our measurements, we set the target number of photons *N*_photons_ to be 1000. The shot-noise associated with counting incoming photons can therefore be accurately quantified. Briefly, the shot-noise distorted potential can be obtained analytically from a real potential (see Supplementary Discussion Section 4 for details)

5 $$\frac{{\phi }_{{{{{{{{\rm{distorted}}}}}}}}}(h)}{{k}_{B}T}=-\ln \left(\alpha {N}_{{{{{{{{\rm{photons}}}}}}}}}\int d{h}_{0}\,{e}^{-\beta {\phi }_{{{{{{{{\rm{real}}}}}}}}}\left({h}_{0}\right)}{p}_{n}({N}_{{{{{{{{\rm{photons}}}}}}}}},{N}_{{{{{{{{\rm{photons}}}}}}}}}{e}^{-\alpha ({h}_{0}-h)})\right)$$

where *α* = (99 nm)^−1^ is a known experimental parameter and *p*_*n*_(*N*_photons_, *n*) is a Poisson distribution with average *n*, inheriting from the shot noise, evaluated for *N*_photons_ = 1000. Taking an infinitely narrow “test” quadratic potential *ϕ*_real_(*h*) yields a noise-broadened *ϕ*_distorted_(*h*) for which the root-mean-squared height fluctuations $\delta h=\sqrt{{\langle {h}^{2}\rangle }_{\phi }} \sim 3\,{{{{{{{\rm{nm}}}}}}}}$. Hence *δh* = 3 nm is our experimental shot-noise-limited resolution.

The dominant source of noise limiting resolution in our experiments is shot noise^56^. Other sources such as Brownian motion distortion are minor. For our laser intensity, the average scattering intensity received is around 1 MHz for a target photon number *N*_photons_ = 1000 and this corresponds to a sampling time step ~1 ms. The vertical diffusion *D*_⊥_ is greatly slowed due to extra friction associated with the flow within the gap. For a spherical particle approaching a solid wall, *D*_⊥_ ≃ *D*_0_(*h*/*a*), where *D*_0_ is the Stokes-Einstein diffusivity for a free particle, *h* is the distance the particle surface is from the wall and *a* is the particle radius^59^. For a DNA-coated sphere approaching a DNA-coated flat surface, this expression ought to work well with *h* the distance between the two brushes, until the separation becomes comparable to the distance between DNA grafts, i.e., for separations ~ 3–10 nm and greater. For *h* ≳ 3–10 nm, the root-mean-square displacement of a particle in Δ*t* = 1 ms is ${{\Delta}}z={(2{D}_{\perp }{{\Delta }}t)}^{1/2}\approx 1$ nm. Therefore, for unbound particles, the dynamical blurring is small, on the order of a nanometer or perhaps a few nanometers for particles far from the substrate, but still very small compared with any relevant length scale, for example, the gravitational height (~100 nm). When the DNA-coated particle is bound to the substrate, the solvent must flow through the porous medium formed by the polymer brushes on the substrate and particle, reducing the vertical displacement of the particle even more. While detailed treatment is beyond the scope of this work, it is clear that when a particle is bound, Δ*z* ≪ 1 nm for Δ*t* ≃ 1 ms. For bound particles, the dynamical blurring is thus negligible compared to the photon counting shot noise.

### Multiscale model

The free energy of the DNA-coated colloid *ϕ*(*h*) is a sum of bulk (gravity) and surface contributions *ϕ*(*h*) = *ϕ*_grav_(*h*) + *ϕ*_surf_(*h*). We account for surface contributions with a Dejarguin approximation^68^ allowing us to relate *ϕ*_surf_(*h*) and the surface interaction energy *φ*(*h*) of two flat walls separated by a distance *h*

6 $${\phi }_{{{{{{{{\rm{surf}}}}}}}}}(h)=2\pi a\int\nolimits_{h}^{\infty }\varphi (h^{\prime} )\,dh^{\prime} \ ,$$

where *a* is the radius of the colloid under consideration. Framed as such, the model is applicable to other geometries, including colloid-colloid interactions where Eq. (6) is simply divided by a factor 2. The surface interaction energy includes binding attraction, steric repulsion and non-specific interactions attributed to van der Waals forces *φ*(*h*) = *φ*_binding_(*h*) + *φ*_steric_(*h*) + *φ*_vdw_(*h*).

To evaluate *φ*_binding_(*h*), we start by calculating the free energy of hybridization of the free chains Δ*G*^(0)^, using standard methods^55^. Entropic contributions strongly depend on the detailed characteristics of the polymer coating, and modify the free energy of binding. We evaluate them by approximating the bound configurations by the product of unbound concentrations, which is reasonable at the high densities involved here (see Supplementary Discussion Section 3.3) as

7 $${{\Delta }}{G}^{{{{{{{{\rm{eff}}}}}}}}}(h)={{\Delta }}{G}^{(0)}-{k}_{B}T\log \left(\frac{\sigma \int\nolimits_{0}^{h}{C}^{{{{{{{{\rm{top}}}}}}}}}(z){C}^{{{{{{{{\rm{bottom}}}}}}}}}(z)dz}{{\rho }_{0}}\right)$$

where *C*^top/bot^(*z*) are respectively the top and bottom normalized concentrations of sticky ends relative to the surface distance *z* and *ρ*_0_ is the elementary concentration. Here the lost degrees of freedom are essentially vertical as the brushes are tightly packed on the surface and well described by a brush (*e.g*. vertical) model. We use a modified version of the MWC theory to express *C*^top/bot^(*z*) (Supplementary Discussion Section 3.3). It contains the detailed measured experimental parameters, such as the brush density *σ*, its molecular weight *M*_*w*_, its persistence length, and its measured equilibrium length. We finally write the free energy of binding as

8 $${\varphi }_{{{{{{{{\rm{binding}}}}}}}}}(h,f)=\sigma f{{\Delta }}{G}^{{{{{{{{\rm{eff}}}}}}}}}(h)+\sigma \left[f\log f+2(1-f)\log (1-f)+f\right]$$

where *f* is the fraction of bound sticky ends. The added terms compared to Δ*G*^eff^(*h*) account for competition between binding partners^40^. In contrast with earlier theoretical work where the fraction *f* of bound sticky ends is determined self-consistently (for example, as the minimizer of *φ*_binding_(*h*))^21,23,39,40^, here we must take into account the fact that bound polymers modify the geometry of the brush, because of excluded volume interactions.

Steric repulsion between opposing polymer brushes is accounted for through a repulsive potential *φ*_steric_(*h*, *f*). Standard theories for steric brush repulsion such as MWC^34,51,52^ have to be carefully extended to account for the complexity of our brush. We account for the fraction *f* of bound brushes^53^ (that modifies the brush’s geometry), heterogeneous brush composition (DNA strands clicked onto PEO), and coating asymmetries (the particle’s brush and coating density are slightly different from the glass)^54^ (Supplementary Discussion Section 3.4). *φ*_steric_(*h*, *f*) therefore includes steric contributions for hybrid, asymmetric brushes with bound fraction *f*, and thus includes the details of the glass (*g*) and the particle (*p*) coatings through their density *σ*_*g*/*p*_, molecular weights ${M}_{w}^{{{{{{{{\rm{PEO}}}}}}}}}$, ${M}_{w,g/p}^{{{{{{{{\rm{DNA}}}}}}}}}$ and persistence length of the PEO and DNA parts. It also allows us to specify the concentration of sticky ends *C*^top/bot^(*z*). Lengthy expressions and detailed methods are reported in Supplementary Discussion Section 3.2–3.4. Note that also that Eq. (8) is extended to account for asymmetric coatings, as reported in Supplementary Discussion Section 3.2–3.4. The fraction of bound sticky ends *f* is then obtained by minimizing *φ*_binding_(*h*, *f*) + *φ*_steric_(*h*, *f*) with respect to *f*.

In Supplementary Discussion Section 3.3 and Section 5.3, we provide a detailed comparison of our approach with existing models to point out similarities and differences.

## Supplementary information

Supplementary information

## Data Availability

The data reported in this study, including all the parameters used to model the data, are available within the article and its Supplementary Information. Source data are provided in this paper.

## Source data

Source data files

## Code availability

The program we developed to calculate DNA-coated interactions is available through GitHub: https://github.com/smarbach/DNACoatedColloidsInteractions. To cite our code, refer to 10.5281/zenodo.6383258.

## References

[1] Mirkin CA, Letsinger RL, Mucic RC, Storhoff JJ (1996). A DNA-based method for rationally assembling nanoparticles into macroscopic materials. Nature.

[2] Alivisatos AP (1996). Organization of ‘nanocrystal molecules’ using DNA. Nature.

[3] Park SY, Lytton-Jean AK, Lee B, Weigand S, Schatz GC (2008). DNA-programmable nanoparticle crystallization. Nature.

[4] Nykypanchuk D, Maye MM, Van Der Lelie D, Gang O (2008). DNA-guided crystallization of colloidal nanoparticles. Nature.

[5] Macfarlane RJ (2011). Nanoparticle superlattice engineering with DNA. Science.

[6] Rogers WB, Shih WM, Manoharan VN (2016). Using DNA to program the self-assembly of colloidal nanoparticles and microparticles. Nat. Rev. Mater..

[7] Subramanian G, Manoharan VN, Thorne JD, Pine DJ (1999). Ordered macroporous materials by colloidal assembly: a possible route to photonic bandgap materials. Adv. Mater..

[8] Vlasov YA, Bo X-Z, Sturm JC, Norris DJ (2001). On-chip natural assembly of silicon photonic bandgap crystals. Nature.

[9] Vogel N (2015). Color from hierarchy: Diverse optical properties of micron-sized spherical colloidal assemblies. Proc. Natl. Acad. Sci..

[10] Yang S (2014). Feedback-driven self-assembly of symmetry-breaking optical metamaterials in solution. Nat. Nanotechnol..

[11] Probst, P. T., Mayer, M., Gupta, V., Steiner, A. M., Zhou, Z., Auernhammer, G. K., König, T. A. & Fery, A. Mechano-tunable chiral metasurfaces via colloidal assembly. *Nat. Mater.* **20**, 1–5 (2021). 10.1038/s41563-021-00991-8 33927391

[12] Qu A (2019). Quantitative zeptomolar imaging of mirna cancer markers with nanoparticle assemblies. Proc. Natl. Acad. Sci..

[13] Chou LY, Zagorovsky K, Chan WC (2014). DNA assembly of nanoparticle superstructures for controlled biological delivery and elimination. Nat. Nanotechnol..

[14] Di Michele L (2013). Multistep kinetic self-assembly of DNA-coated colloids. Nat. Commun..

[15] Joshi D (2016). Kinetic control of the coverage of oil droplets by DNA-functionalized colloids. Sci. Adv..

[16] Biancaniello PL, Kim AJ, Crocker JC (2005). Colloidal interactions and self-assembly using DNA hybridization. Phys. Rev. Lett..

[17] Rogers WB, Manoharan VN (2015). Programming colloidal phase transitions with DNA strand displacement. Science.

[18] Song M, Ding Y, Zerze H, Snyder MA, Mittal J (2018). Binary superlattice design by controlling DNA-mediated interactions. Langmuir.

[19] He M (2020). Colloidal diamond. Nature.

[20] Rogers WB, Sinno T, Crocker JC (2013). Kinetics and non-exponential binding of DNA-coated colloids. Soft Matter.

[21] Mognetti BM (2012). Predicting DNA-mediated colloidal pair interactions. Proc. Natl. Acad. Sci..

[22] Dreyfus R (2010). Aggregation-disaggregation transition of DNA-coated colloids: experiments and theory. Phys. Rev. E.

[23] Angioletti-Uberti S, Mognetti BM, Frenkel D (2016). Theory and simulation of DNA-coated colloids: a guide for rational design. Phys. Chem. Chem. Phys..

[24] Wu K-T (2013). Kinetics of DNA-coated sticky particles. Phys. Rev. E.

[25] Kim AJ, Manoharan VN, Crocker JC (2005). Swelling-based method for preparing stable, functionalized polymer colloids. J. Am. Chem. Soc..

[26] Wang Y (2015). Synthetic strategies toward DNA-coated colloids that crystallize. J. Am. Chem. Soc..

[27] Wang Y (2012). Colloids with valence and specific directional bonding. Nature.

[28] Gong Z, Hueckel T, Yi G-R, Sacanna S (2017). Patchy particles made by colloidal fusion. Nature.

[29] Ducrot E, He M, Yi G-R, Pine DJ (2017). Colloidal alloys with preassembled clusters and spheres. Nat. Mater..

[30] Lin H (2017). Clathrate colloidal crystals. Science.

[31] Rogers WB, Crocker JC (2011). Direct measurements of DNA-mediated colloidal interactions and their quantitative modeling. Proc. Natl. Acad. Sci..

[32] Angioletti-Uberti, S. Understanding the self-assembly of DNA-coated colloids via theory and simulations. *Front. Nanosci*. **13**, 87–123 (2019).

[33] Merminod, S., Edison, J. R., Fang, H., Hagan, M. F. & Rogers, W. B. Avidity and surface mobility in multivalent ligand-receptor binding. *Nanoscale* **13**, 12602–12612 (2021). 10.1039/d1nr02083h PMC8386892 34259699

[34] Milner ST (1988). Compressing polymer “brushes”: a quantitative comparison of theory and experiment. EPL (Europhysics Letters).

[35] Parsegian, V. A. Van der Waals forces: a Handbook for Biologists, Chemists, Engineers, and Physicists (Cambridge University Press, 2005).

[36] Nykypanchuk D, Maye MM, Van Der Lelie D, Gang O (2007). DNA-based approach for interparticle interaction control. Langmuir.

[37] Hueckel T, Hocky GM, Palacci J, Sacanna S (2020). Ionic solids from common colloids. Nature.

[38] Dreyfus R (2009). Simple quantitative model for the reversible association of DNA coated colloids. Phys. Rev. Lett..

[39] Varilly P, Angioletti-Uberti S, Mognetti BM, Frenkel D (2012). A general theory of DNA-mediated and other valence-limited colloidal interactions. J. Chem. Phys..

[40] Angioletti-Uberti S, Varilly P, Mognetti BM, Tkachenko AV, Frenkel D (2013). Communication: a simple analytical formula for the free energy of ligand–receptor-mediated interactions. J. Chem. Phys..

[41] Lowensohn J, Hensley A, Perlow-Zelman M, Rogers WB (2020). Self-assembly and crystallization of DNA-coated colloids via linker-encoded interactions. Langmuir.

[42] Fang H, Hagan MF, Rogers WB (2020). Two-step crystallization and solid–solid transitions in binary colloidal mixtures. Proc. Natl. Acad. Sci..

[43] Wang Y (2015). Crystallization of DNA-coated colloids. Nature Communications.

[44] Oh JS, Wang Y, Pine DJ, Yi G-R (2015). High-density PEO-b-DNA brushes on polymer particles for colloidal superstructures. Chem. Mater..

[45] Vos R (2018). Chemical vapor deposition of azidoalkylsilane monolayer films. Langmuir.

[46] Prieve DC, Frej NA (1990). Total internal reflection microscopy: a quantitative tool for the measurement of colloidal forces. Langmuir.

[47] Prieve DC (1999). Measurement of colloidal forces with TIRM. Adv. Colloid Interface Sci..

[48] Valignat M-P, Theodoly O, Crocker JC, Russel WB, Chaikin PM (2005). Reversible self-assembly and directed assembly of DNA-linked micrometer-sized colloids. Proc. Natl. Acad. Sci..

[49] Xu Q, Feng L, Sha R, Seeman N, Chaikin P (2011). Subdiffusion of a sticky particle on a surface. Phys. Rev. Lett..

[50] Youssef M, Morin A, Aubret A, Sacanna S, Palacci J (2020). Rapid characterization of neutral polymer brush with a conventional zetameter and a variable pinch of salt. Soft Matter.

[51] Milner ST, Witten T, Cates M (1988). Theory of the grafted polymer brush. Macromolecules.

[52] Drobek T, Spencer ND, Heuberger M (2005). Compressing PEG brushes. Macromolecules.

[53] Meng X-X, Russel WB (2003). Telechelic associative polymers: interactions between strongly stretched planar adsorbed layers. Macromolecules.

[54] Shim D, Cates M (1990). Forces between asymmetric polymer brushes. J. Phys..

[55] SantaLucia J (1998). A unified view of polymer, dumbbell, and oligonucleotide DNA nearest-neighbor thermodynamics. Proc. Natl Acad. Sci..

[56] Cui F, Pine DJ (2022). Effect of photon counting shot noise on total internal reflection microscopy. Soft Matter.

[57] Wang X, Ramírez-Hinestrosa S, Dobnikar J, Frenkel D (2020). The Lennard-Jones potential: when (not) to use it. Phys. Chem. Chem. Phys..

[58] Das A, Limmer DT (2021). Variational design principles for nonequilibrium colloidal assembly. J. Chem. Phys..

[59] Bevan MA, Prieve DC (2000). Hindered diffusion of colloidal particles very near to a wall: revisited. J. Chem. Phys..

[60] Gardiner, C. W. et al. *Handbook of Stochastic Methods*, vol. 3 (Springer Berlin, 1985).

[61] Brenner H (1961). The slow motion of a sphere through a viscous fluid towards a plane surface. Chem. Eng. Sci..

[62] Lee-Thorp JP, Holmes-Cerfon M (2018). Modeling the relative dynamics of DNA-coated colloids. Soft Matter.

[63] Licata NA, Tkachenko AV (2007). Colloids with key-lock interactions: nonexponential relaxation, aging, and anomalous diffusion. Phys. Rev. E.

[64] Wang Y, Jenkins IC, McGinley JT, Sinno T, Crocker JC (2017). Colloidal crystals with diamond symmetry at optical lengthscales. Nat. Commun..

[65] Sakai T, Nishimura SI, Naito T, Saito M (2017). Influenza a virus hemagglutinin and neuraminidase act as novel motile machinery. Sci. Rep..

[66] Müller M, Lauster D, Wildenauer HH, Herrmann A, Block S (2019). Mobility-based quantification of multivalent virus-receptor interactions: New insights into influenza a virus binding mode. Nano Lett..

[67] Hiki S, Kataoka K (2007). A facile synthesis of azido-terminated heterobifunctional poly (ethylene glycol) s for “click” conjugation. Bioconjugate Chem..

[68] Derjaguin BV (1934). Untersuchungen über die reibung und adhäsion, IV. Kolloid-Zeitschrift.

