# Supplementary material for: Comprehensive view of microscopic interactions between DNA-coated colloids
Source: Nat Commun. 2022 Apr 28;13:2304. doi: 10.1038/s41467-022-29853-w (PMC9051097; doi:10.1038/s41467-022-29853-w)
Supplement: Supplementary file 1 — Supplementary information [file 41467_2022_29853_MOESM1_ESM.pdf]

# Supplementary Information

F. Cui, S. Marbach, J. A. Zheng, M. Holmes-Cerfon, D. J. Pine

## Supplementary Discussion


**1 Total internal reflection microscope (TIRM) design and measurements 2**
1.1 TIRM optical train and sample system . . . 2
1.2 Single-particle potential energy measurement . . . 3
1.3 Absolute separation distance calibration . . . 3
1.4 Ensemble melting curve measurement using TIRM camera . . . 4

**2 Polymer brush characterization and modeling 5**
2.1 Characterization of PEO polymer lengths via electrophoretic mobility measurements . . 5
2.2 Characterization of coating densities . . . 7
2.3 PEO chains are well described by a brush model . . . 7
2.4 Modeling of heterogeneous (PEO + ssDNA) polymer chains . . . 9

**3 Model for free energy of interaction of DNA-coated particles 11**
3.1 Free energy of interaction . . . 11
3.2 From surface free energy to melting curves . . . 13
3.3 Surface interactions associated with covalent binding . . . 15
3.4 Unified theory with steric repulsion and binding . . . 23
3.5 van der Waals interactions . . . 29
3.6 Surface charge electrostatic interactions . . . 30
3.7 Inferring microscopic parameters . . . 32

**4 Accounting for shot noise on model curves 34**
4.1 Shot noise on model curves . . . 34
4.2 Shot noise does not affect melting curves . . . 36

**5 Model parameters and uncertainty evaluation 37**
5.1 Recapitulation of experimental parameters used in the model . . . 37
5.2 Sensitivity of the model on physical parameters . . . 37
5.3 Analysis of modeling choices . . . 40

**6 Correspondence of microscopic model potential profiles to standard interaction profiles 41**

**7 Agreement of model predictions with previous experimental measurements 43**
7.1 Experiments from Rogers & Crocker[1] . . . 43
7.2 Experiments from Xu *et al.*[2] . . . 46

# 1 Total internal reflection microscope (TIRM) design and measurements

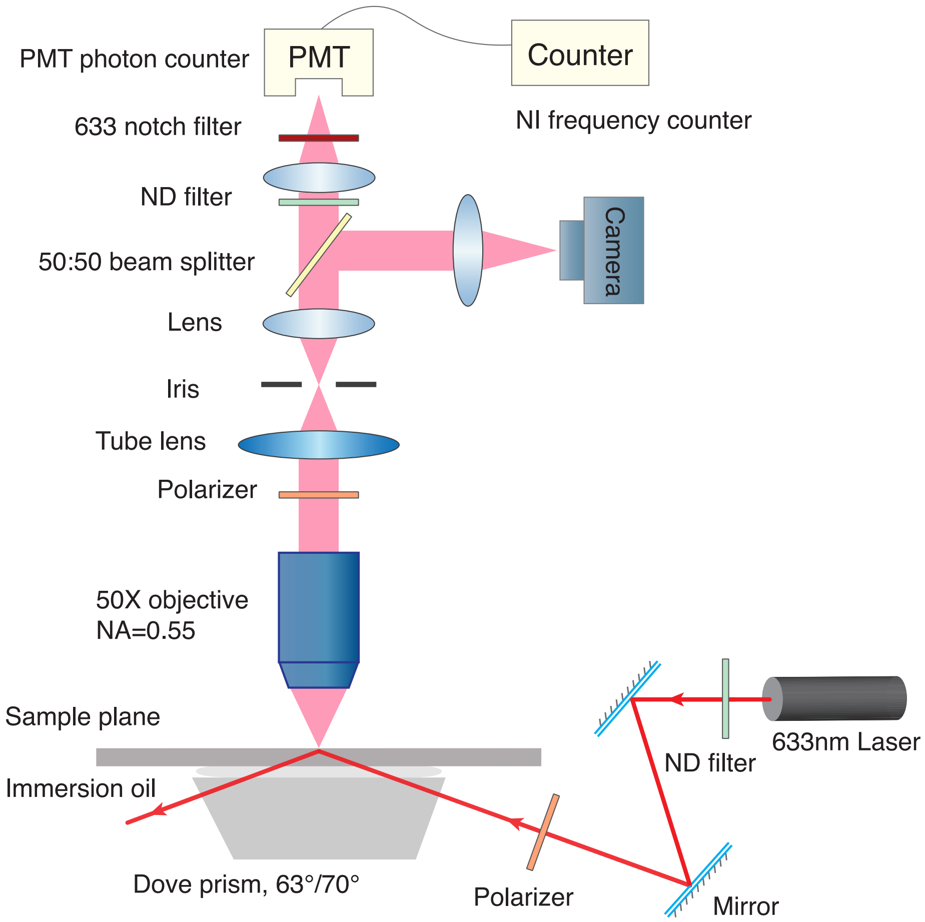


**Figure S1:** TIRM Optical train.

## 1.1 TIRM optical train and sample system

Figure S1 shows a schematic of the TIRM setup. The microscope is custom built in our lab using standard cage-system parts from Thorlabs. We use a linearly polarized HeNe laser (Lumentum) as our light source which has a maximum power output of 30 mW. The incident laser power is attenuated as needed with absorption ND filters. The laser is coupled into the measuring cell using a dove-shaped glass prism. The prism is designed to have asymmetric angles (63°/70°) to minimize background scattering from reflected beams. The laser incident angle is set to be 70° which produces an exponentially-decaying evanescent wave in the sample with a penetration depth of 100 nm. We align the incident beam with a pair or mirrors that has precision linear and angular adjustment.

The scattered light is collected using a 50× Mitutoyo air objective (Apo Plan 50X, 0.55NA) with a working distance of 13 mm. At the imaging plane after the tube lens, we place a removable aperture to define the field of view as well as to exclude scattered light from the outside of the focal plane. In a typical experiment, an aperture with a diameter of 1.8 mm is used, corresponding to a circular field of view with 40 $\mu$m in diameter. The collected light is further spit using a 50/50 beamsplitter, with one path going into a photomultiplier tube (PMT) photon counter (Hamamatsu, H7421-40) and the other forming and image on a CMOS camera (AMScope, MU1803). An ND (1.0) filter and a 633 nm notch filter are placed in front of the PMT. We use a National Instruments frequency counter (USB-6341) to measure

the received photon frequencies. The counter is programmed and controlled by Labview software. We adopted the "target photon number" method, also termed the "two counter large range" method[3], where the counter counts up to a certain number of photons to calculate a frequency.

Our TIRM is equipped with a motorized scanning stage (H101a, Prior scientific). We use HybriWel Sealing system (Grace bio-labs, 250 $\mu$m depth) as our measurement sample cell. A polished glass slide with indium-tin-oxide (ITO) coating is used as the sample substrate, with the glass side functionalized with DNA and facing the sample solution and the ITO side facing the glass prism. The ITO coating (30-60 ohms) is used as a resistance heater and connected to a programmable temperature controller (Thorlab TC200) with its temperature sensor placed near the sample cell. The actual temperature inside the sample cell is calibrated using a temperature monitor (Physitemp TH-8) with a needle probe inserted into the center of the sample cell solution. Calibration is carried out after each measurement.

## 1.2 Single-particle potential energy measurement

For a single-particle potential measurement, a dilute polystyrene (PS) particle solution ($\sim$ 0.002 w/v%) is injected in the sample cell which is then sealed off completely. Before the measurement, the sample is translated to move one particle to the center of the field of view and aligned until the maximum scattering intensity is reached. The laser power is adjusted using the ND filters until the maximum scattering intensity of the single particle is around 1.3 MHz. The incident laser light is set to p-polarization by a half-wave plate and a polarizer (p) is placed in the collection path to further exclude s-polarized light[4].

During the measurement, we use live video from the camera to track the particle. The particle of interest is kept in the center of the defined field of view by translating the sample stage. The measurement of a typical potential curve usually takes about 15 minutes to ensure we acquire enough data points ($> 350,000$) and to minimize statistical error.

## 1.3 Absolute separation distance calibration

The absolute separation distance $h$ of a colloid in an evanescent field can be written as:

$$h = -\frac{1}{\alpha} \ln \frac{I(h)}{I_0}, \tag{S1}$$

where $I_0$ is the scattering intensity when $h = 0$ and $\alpha^{-1} = 99$ nm is the penetration depth of the evanescent field. However, in the case of DNA coated colloids, the value of $I_0$ cannot be directly measured. The dense layer of polymer brush and DNA chains prohibits particles from getting into intimate contact with the wall[5]. Moreover, it has been demonstrated that the scattering decay of a particle in an evanescent field deviates from its exponential form when the particle comes too close to the surface ($<$ 20 nm)[4,6]. This makes the determination of absolute height from the intimate-contact method subject to considerable error. We instead calibrate the absolute distance with scattering intensity $I_{\mathrm{cal}}$ of particles with known separation distance $h_{\mathrm{cal}}$ from the surface. The separation distance $h$ can be calculated from calibration intensity:

$$h = -\frac{1}{\alpha} \ln \frac{I(h)}{I_{\mathrm{cal}}} + h_{\mathrm{cal}}. \tag{S2}$$

We choose PS spheres coated with F127 pluronic surfactant as our calibration particles. Bare 5-$\mu$m PS particles are dispersed in 140 mM PBS solution containing 0.3% F127. F127 is known to adsorb onto the PS surface and forms a PEO layer of height[7] $L_{\mathrm{PS}}^{\mathrm{F127}} \sim 10$ nm (see Sec. 2.1.3). The particles are injected into a sample cell with the bottom glass surface coated with 11-azidoundecyltrimethoxysilane. F127 also adsorbs onto the hydrophobic, silane-treated surface which has a contact angle of $\sim 80^{\circ}$[8]. The length of the adsorbed F127 layer on a surface with such hydrophobicities is roughly of height $L_{\mathrm{substrate}}^{\mathrm{F127}} \sim 6$ nm[9]. We then measure the averaged scattering intensities at the potential minimum over 20 different particles as $I_{\mathrm{cal}}$, which we measured to be $1.3579 \pm 0.0517$ MHz for one specific optical setting. The corresponding separation distance $h_{\mathrm{cal}} = L_{\mathrm{PS}}^{\mathrm{F127}} + L_{\mathrm{substrate}}^{\mathrm{F127}} = 16$ nm.

To get the absolute position of a DNA-coated particle as shown in Fig. 4a and b in the main text, we measure the averaged potential-minimum scattering intensities, $\bar{I}_{22^{\circ}C}$, from 20 particles coated with 100% DNA at 22°C and calculate the averaged relative distance, $\bar{h}_{22^{\circ}C}$. The absolute distance calibration is carried out with the same optical setting for all samples. For the sample tested in Fig. 4a, the average scattering intensity $\bar{I}_{22^{\circ}C} = 1.14 \pm 0.10$ MHz, and from Eq. (S2) and considering uncertainty propagation, we calculate $\bar{h}_{22^{\circ}C} = 33$ nm with a standard error of 1.9 nm. For the colloids with 100% sticky DNA coverage shown in Fig. 4b, the average scattering intensity $\bar{I}_{22^{\circ}C} = 1.19 \pm 0.11$ MHz, and hence $\bar{h}_{22^{\circ}C}$ = 29 nm with an error of 1.5 nm. The height of colloids at other temperatures or with other percentage of sticky ends (Fig. 4b) is calculated based on the calibrated $\bar{h}_{22^{\circ}C}$. Note that the particles in Fig. 4a and 4b equilibrate at different heights (4 nm difference) at the same temperature even with the same surface configurations. This can be attributed to the slight variation in the DNA/brush coverage density. The variation is also consistent with the small shift in the melting temperature ($T_m$ = 61.5 °C for Fig. 4a and $T_m$ = 58.2 °C for Fig. 4b).

## 1.4 Ensemble melting curve measurement using TIRM camera

When DNA-coated colloids aggregate, the melting curve is typically characterized by the percentage of unpaired particles (singlets) at different temperatures. The melting temperature $T_m$ corresponds to the temperature where 50% of the observed particles are unbound singlets.[10,11] Here we adopt a similar definition and plot the fraction of "melted" particles as a function of the temperature. In the particle-substrate geometry, a particle is regarded as "melted" when it has lifted off from the surface. Note that compared to other definitions of melting, we use a "lifting-off" criteria instead of a "lateral moving" criteria to better represent the actual separation of particles[2]. This is closer to the complete separation criterion required to be counted as a singlet when looking at DNA-coated colloids aggregates.

To measure the melting curve of our DNA-coated colloids, we use the camera on our TIRM with the same illumination and temperature control system as used in the potential measurements. The temporal and spatial resolution of a camera are less than when using single-particle PMT detection, but both are more than sufficient to detect liftoff . More concentrated PS dispersion (~ 0.2 w/v%) is used for melting curve measurements (~ 15 particles in the field of view). At each temperature, we take a video of ~ 200 DNA-coated particles using the camera on TIRM and count the percentage of the particles that lift off at least once during a one-minute observation window. A particle is considered "lifted off" if its brightness weakens in the video, which corresponds to at least 20 nm beyond the potential minimum. Videos are recorded at 14 frames/s. Fig. S2 shows the selected snapshots of 13 particles during the one-minute observation window at the melting temperature. 7 of the 13 particles are considered "melted".

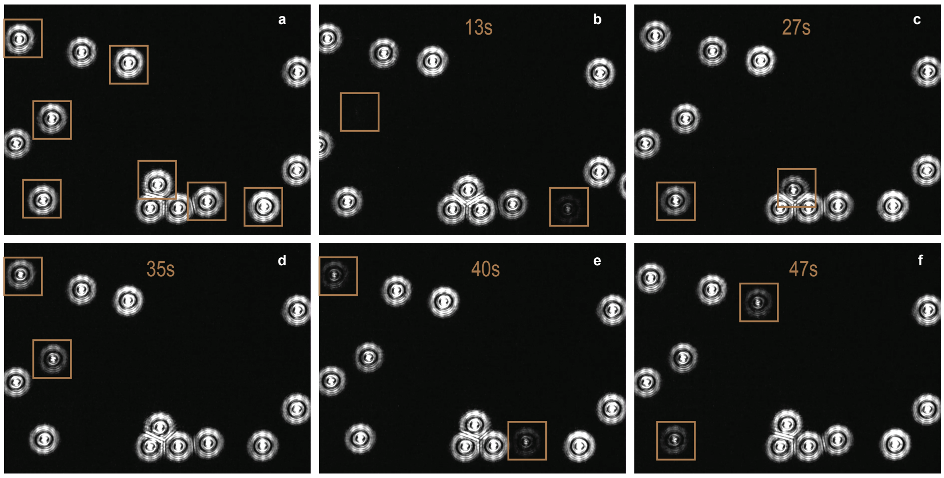


**Figure S2:** Selected TIRM images of DNA-coated PS particles (with 34k PEO nad $A^6/B^6$) when temperature is 60.5 °C during the measurement of the melting curve, where a, summarizes the melted particles within the observation window of this field of view, and b-f are snapshot images at different time during the observation.

# 2 Polymer brush characterization and modeling

## 2.1 Characterization of PEO polymer lengths via electrophoretic mobility measurements

In this section, we describe the PEO polymer brushes and the measurements of the length of PEO brushes using an electrophoretic mobility method[12].

### 2.1.1 Materials

Polystyrene-b-poly(ethylene oxide) copolymer (PS-b-PEO) with an azide ($N_3$) as the PEO end group is used to create a PEO brush on the polystyrene (PS) particle surface. The incorporation of the polymer brush is described in the Methods section. Briefly, as-received PS-b-PEO copolymers (Polymer Source) with various molecular weights $M_w$ are first functionalized with $N_3$ and then physically grafted to the surface of 1-$\mu$m-diameter PS particles (purchased from Thermo Scientific) using a swelling-deswelling approach that embeds the PS block within the PS particle leaving the PEO water-soluble block extending out from the particle to form a dense PEO polymer brush[13,14]. We prepare particles with the following PS-b-PEO brushes:

| PS-b-PEO, $M_w$ [g/mol] | PS, $M_w$ [g/mol] | PEO, $M_w$ [g/mol] |
|---|---|---|
| 11,124 | 3,800 | 6,500 |
| 15,336 | 3,200 | 11,000 |
| 39,690 | 3,800 | 34,000 |

**Table S1:** Molecular weights of polymers used to form brushes

### 2.1.2 Measuring PEO lengths

To determine the PEO brush length, the electrophoretic mobility $\mu$ of bare particles and particles with different lengths of grafted PEO brushes is measured using a Malvern Zetasizer for salt solutions with various concentrations. We prepare solutions with NaCl concentrations ranging from 500 mM to 0.01 mM, which corresponds to Debye lengths $\lambda_D$ ranging from $\sim$ 0.43 nm to 96.1 nm.

The length of the polymer brush $L$ and the Brinkman length $\xi$ characterizing the porosity of the brush are then found by fitting our experimental mobility data $\hat{\mu}_i = \mu_i/\mu_{\text{bare}}$ with respect to the Debye length $\lambda_D$ using a modified Henry function[12,15], where $i$ = 1, 2, or 3, denotes the PS-b-PEO types and $\mu_{\text{bare}}$ represents the electrophoretic mobility of the bare particle without any brush. The modified Henry function, which is the basis of both equations, is valid for low electrophoretic mobility; thus we only retain mobility values under 2.5 $\mu\text{m} \cdot \text{cm/V} \cdot \text{s}$ for each sample. The data and corresponding fits are reported in Fig. S3. The fitting method is a least-squares minimization and the uncertainty range reported corresponds to the min/max values obtained over repeated experiments). We find:

| PEO $M_w$ [g/mol] | PEO brush thickness, $L$ [nm] | Brinkman length, $\xi$ [nm] |
|---|---|---|
| 6,500 | 12.8 ± 0.2 | 3.3 ± 0.1 |
| 11,000 | 16.2 ± 0.5 | 4.2 ± 0.2 |
| 34,000 | 29.6 ± 2 | 7.9 ± 0.7 |

**Table S2:** PEO brush thickness and Brinkman length

The values obtained show a consistent length and porosity increase with increasing number of PEO units in the chain.

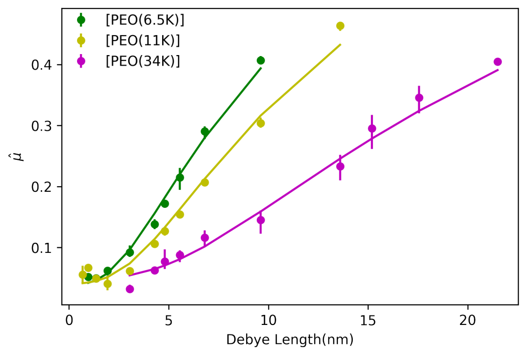


**Figure S3:** Variations of $\hat{\mu} = \mu/\mu_{\text{bare}}$ with $\lambda_D$ for different PEO brushes and fits according to Eq. (3) of Ref. 12 with parameters reported in the main text. Each data point is an average over 3 independent measurements. $\mu$ is the electrophoretic mobility of coated particles and $\mu_{\text{bare}}$ that of bare particles.

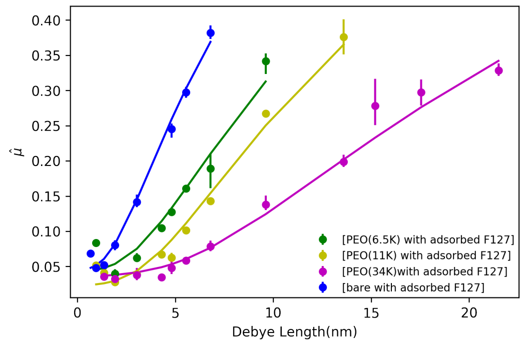


**Figure S4:** Variations of $\hat{\mu} = \mu/\mu_{\text{bare}}$ with $\lambda_D$ for different PEO brushes, pre-soaked in 0.3%wt F127 and fits according to Eq. (3) of Ref. 12 with parameters reported in the main text. Each data point is an average over 3 independent measurements.

### 2.1.3 Measuring PEO lengths with adsorbed F127

In the total internal reflection microscopy (TIRM) experiments, Pluronic F127 (0.3%) is added in the measurement solution to mimic the DNA-coated colloids assembly environment where Pluronic compounds are often added as surfactants [11,14,16]. Since Pluronic F127 strongly adsorbs on bare colloids, we seek its effect on our brush-coated colloids . We first test the adsorption of F127 on bare PS particles. Bare particles (diameter 1$\mu$m) are incubated at 0.3%wt F127 solution for 24 hours while gentle shaking (600 rpm), and subsequently washed with de-ionized water 4 times. We note that 0.3%wt is enough to reach adsorption saturation which is consistent with literature reports [17]. Electrophoretic measurements similar to Sec. 2.1.2 yield that adsorbed F127 on the bare particle has a length $L_{\text{PS}}^{\text{F127}} = 9.9 \pm 0.1 nm$.

We also adsorb F127 onto the colloids that have different terminally anchored PEO, as described in Sec. 2.1.1, using the same procedure used with the bare particles. The mobility data and corresponding fits for all cases of F127 adsorption are reported in Fig. S4. We find:

| PEO $M_w$ [g/mol] | Brush thickness, $L$ [nm] | Brinkman length, $\xi$ [nm] |
|---|---|---|
| 6,500 | 16.6 ± 1.4 | 4.3 ± 0.4 |
| 11,000 | 18.6 ± 1.0 | 4.2 ± 0.3 |
| 34,000 | 33.0 ± 2.7 | 8.2 ± 0.7 |

**Table S3:** PEO brush thickness and Brinkman length with adsorbed F127

We observe that after adsorption of F127, the brush-coated particles brush lengths increase by about 2–4 nm. The increase in height is notably smaller than the intrinsic height of adsorbed F127 (~10 nm) on a bare particle. Further, we do not observe a clear trend of the increased length with the PEO brush $M_w$; the increase in length is independent of $M_w$ to within experimental uncertainty suggesting that the increased height is more a characteristic of the F127 molecule than that of the PEO brush.

## 2.2 Characterization of coating densities

**Particle coating densities** After coating the particles with a PEO brush, single-stranded DNA (ssDNA, 20 bases) containing internal Cy5 fluorophores and dibenzocyclooctyne end modification is clicked on PEO tips through strain promoted alkyne-azide cycloaddition (SPAAC)[14]. During the clicking procedure, we add excess amounts of ssDNA (typically 1 nmol of DNA oligomers for 200 $\mu$L of 0.3 w/v% PS particles) and allow the reaction to occur over a sufficient period of time (~48 hours) in order to maximize the DNA coverage. Subsequently, the particles are dispersed in PBS buffer at pH 7.4. The suspension is then loaded to a BD LSR II flow cytometer (BD Biosciences) to measure the fluorescence intensity of the fluorescent DNA.

A calibration curve is obtained by measuring the five standards (Quantum™ Cy5) purchased from Bangs Laboratories. The number of DNA strands per particle is then determined by comparing the fluorescence intensity of each sample to the calibration curve. The average total number of DNA is measured to be $2.94 \times 10^5$ DNA per 1.0 $\mu$m particle on 6.5k PEO coated particles ($\sigma_{6.5k}^{-1}$ = (3.27 nm)$^2$), and $2.43 \times 10^5$ DNA per 1 $\mu$m particle on 11k PEO coated particles ($\sigma_{11k}^{-1}$ = (3.6 nm)$^2$), and $6.61 \times 10^4$ DNA per 1 $\mu$m particle on 34k PEO coated particles ($\sigma_{34k}^{-1}$ = (6.9 nm)$^2$).

Notably, we assume the fluorescent DNA molecule has a roughly one-to-one ratio with the PEO chain. This takes into account of the high conversion of both the azide functionalization[13] and the SPAAC reaction[18].

**Glass surface coating densities** It is not possible to measure coating densities in a similar way for the glass coated surface, yet it is possible to assess the relative coating densities comparing the two fabrication methods (Method A: using 11-azidoundecyltrimethoxysilane as DNA linker on glass or Method B: using polymer-brush mediated approach as used on PS particles). We perform fluorescent imaging of DNA-functionalized glass slides with the two techniques – see Fig. S5. We find that coating densities with Method A are consistently lower compared to Method B by 30%. Coating densities on the glass surface are thus lower than that on particles because of the differences in the coating process. Crowding on the flat glass surface due to absence of curvature present on the particles may also decrease further the glass coating densities compared to particles.

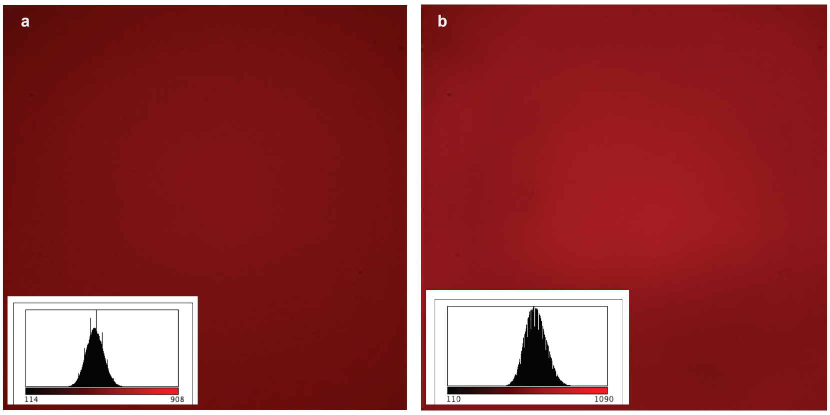


**Figure S5:** Fluorescent imaging of two DNA-functionalized glass slides. **a**, using 11-azidoundecyltrimethoxysilane as DNA linker and **b**, using polymer-brush (6.5k PEO) mediated approach similar to the PS particle surface. Insets show the histogram of fluorescence intensities, in normalized arbitrary units, of each image. **a** has a mean of 474 while **b** has a mean of 650.

## 2.3 PEO chains are well described by a brush model

A preliminary important step is to find the best model to describe the heterogeneous polymer chains made of PEO and ssDNA. To do so we focus on the PEO chain first. Importantly, here we show that length and density measurements reported in Sec. 2.1 and 2.2 allow us to confirm that the polymer chain

is best described by a brush model, notably that of Milner, Witten, and Cates, and not a mushroom model.

In the expected range of coating densities, the chains may either adopt a mushroom or brush configuration on the surfaces[14]. We review briefly models for these configurations with a focus on PEO.

**Mushrooms** If the PEO chain is not too densely coated, we expect PEO to to be described by a mushroom model near the surface.

For PEO one can use an ideal chain model extended to account for typical favorable angle configurations between bonds[19]. In that case the end-to-end distance of PEO is

$$R^2_{\mathrm{ideal}} = C_\infty \times N_{\mathrm{PEO}}(3\ell_{\mathrm{PEO}})^2 , \tag{S3}$$

where $N_{\mathrm{PEO}}$ is the number of monomers, $\ell_{\mathrm{PEO}} = 0.151\,\mathrm{nm}$ is the average of the squares of the bond lengths and $C_\infty = 4.1$ is a constant specific to PEO[20].

To account in more detail for the chain's specifics and its immersion in a good solvent one can also use a Flory type model

$$R^2_{\mathrm{Flory}} = b_{\mathrm{PEO}} N^{3/5}_{\mathrm{PEO}} . \tag{S4}$$

The segment length $b_{\mathrm{PEO}} = 0.368$ nm for PEO is calculated from its chemical structure[21] and is in close agreement with other reported values[19,20].

Values of the end-to-end radius using either Eq. (S3) or Eq. (S4) for the different PEO chain lengths explored in this work are reported in Table S4.

**Brushes – Milner, Witten, Cates (MWC) model** If the PEO chain is densely coated, we expect PEO to form a brush near the surface.

Using a Milner-Witten-Cates model for the brush we can calculate the height of the chain within the brush as[22–24]

$$h_{\mathrm{eq}} = \left( \frac{12}{\pi^2}\, \sigma\, b^2_{\mathrm{PEO}}\, \omega \right)^{1/3} N_{\mathrm{PEO}} , \tag{S5}$$

where $N$ is the number of segments composing the polymer, with $b_{\mathrm{PEO}}$ the segment length and $\omega$ the excluded volume parameter (which here is taken in units of volume).

The excluded volume $\omega$ can be calculated from osmotic pressure of the polymer solutions with similar concentration[22]:

$$\Pi(\Phi) = \tfrac{1}{2}\, \omega\, \Phi^2 , \tag{S6}$$

where $\Pi$ is the osmotic pressure, $\Phi$ is the concentration of monomers per unit volume, and $\omega$ is the excluded volume. Osmotic measurements[25] for PEO ($M_w \sim 6000$ g/mol) solution with mass density $\Phi = 8$ wt% (corresponding to the polymer concentration near the grafting surface) give $\Pi = 65000$ Pa. Using Eq. (S6) we obtain the effective excluded volume $\omega = 0.027$ nm$^3$. It is worth noting that $\omega$ tends to be smaller for PEO with larger $M_w$ in dilute PEO solutions[26,27]. Here we assume the same value for all PEO brush types as the simplest model. The calculated height from the brush model Eq. (S5) (using $\sigma$ measured as described in Sec. 2.2) is reported in Table S4 as $h^{\mathrm{ideal}}_{\mathrm{eq}}$.

**Comparison with experiments** In Table S4, we compare the experimental measurements as reported in §2.1 to the calculated chain lengths for both the mushroom and the brush models. We observe that the measured polymer lengths $L$ clearly exceed the lengths predicted with mushroom models while they agree remarkably well with the brush model. The overestimation of the height of PEO with $M_w$ = 34000 g/mol is expected due to the overestimation of $\omega$ as discussed above[26,27].

This suggests that samples are indeed in a densely coated regime. In fact the end-to-end radius calculated $R$ shows that the typical space required for a mushroom $R^2 \sim 100$ nm$^2$ clearly exceeds the typical space per polymer on the surface $\sigma^{-1} \sim 10$ nm$^2$. According to Ref. 28, this criteria alone allows one to deduce that polymers are arranged in a brush conformation.

[1]counting tetrahedral angles, $L_{\mathrm{tot}} = N b_{\mathrm{PEO}}$

| $M_w$ (g/mol) | PEO units $N_{PEO}$ | Contour length[1] $L_{tot}$ [nm] | $R_{ideal}$ [nm] | $R_{Flory}$ [nm] | $h_{eq}^{ideal}$ [nm] | $L$ [nm] exp. data |
|---|---|---|---|---|---|---|
| 6,500 | 148 | 54.3 | 6.4 | 7.4 | 11.0 | 12.8 |
| 11,000 | 250 | 91.9 | 8.4 | 10.1 | 17.4 | 16.2 |
| 34,000 | 772 | 284.1 | 14.7 | 19.9 | 35.0 | 29.6 |

**Table S4:** End to end radii or height of typical polymers used here as calculated using (1) the worm-like ideal chain model corrected for pair-wise interactions, (2) with the Flory model, and (3) with the Milner-Witten-Cates theory.

## 2.4 Modeling of heterogeneous (PEO + ssDNA) polymer chains

We explain further choices for modeling the heterogeneous polymer brush consistently with experimental findings.

### 2.4.1 Modeling the effect of adsorbed F127 on polymer (PEO) brushes

In all experiments, coated particles are studied in an aqueous suspension with 0.3% wt F127, which adsorbs onto the PS surface, which causes the grafted PEO strands to extend further into the solution. To account for this effect, we seek a simple yet representative *ansatz* as a detailed account of such a bimodal brush is beyond the scope of this work.

As shown in §2.1, F127 adsorbs on all brush-coated particles, contributing to about 2–4 nm of increased brush height. F127 is a PEO-PPO-PEO block copolymer. When mixed with PS particles, the hydrophobic PPO block adsorbs onto the PS surface while PEO units extend out into the solution. Here, we assume that the PEO units from F127 occupy vacant sites within the brush layer of PEO-coated colloids, which extends the polymer brush as a whole, as suggested by the experimental data presented in Tables S2 and S4. In the model, the resultant brush layer is treated as a homogeneous PEO brush layer with modified height and density. This assumption is reasonable because both F127 and PS-PEO contribute similar chemical units (PEO) to the brush layer. PEO has high solubility in water. In the relatively low mass density region ($<$ 10 wt% of PEO), the PEO units from F127 and PS-PEO have enough space to rearrange and thus form an evenly distributed PEO solution. This assumption could be less effective when the brush is highly compressed and local mass density increases, as explored in Fig. 4 of the main text.

In the model, we use a uniform effective brush, described by Milner-Witten-Cates theory[24], to model PEO polymer brushes entangled with F127. Importantly, to calculate the free energy of interaction, within the Milner-Witten-Cates theory, the brush model only requires 4 parameters, either $(\omega, N, b, \sigma)$ or $(h_{eq}, N, b, \sigma)$ where we recall that $h_{eq}$ is the brush height, $\omega$ the excluded volume parameter, $N$ the number of units, $b$ the segment length and $\sigma$ the density of the brush. Here, as we have a measure of $h_{eq}$, we use the $(h_{eq}, N, \ell, \sigma)$ description.

For the effective brush with entangled F127 we make the following choices

- the number of units is given by the number of PEO units in the chain $N = N_{PEO}$
- the segment length is naturally kept to be that of PEO $b = b_{PEO}$
- the heights $h_{eq}$ are chosen to be within the range of electrophoresis-based length measurements (including adsorbed F127). Note that the actual coating properties of PS-PEO may vary slightly from sample to sample causing $h_{eq}$ to vary slightly as well. To better represent the polymer brush used in specific TIRM measurements, in modeling, we make a slight correction to the heights based on van der Waals potential measurements at high temperature for the same batch of particles. The chosen heights are reported in Table S5 (see $h_{eq}^{PEO+F127}$) and are largely within the uncertainty range of electrophoresis-based measurements.

- the density is increased by a constant amount to mimic F127 adsorption on the surface: $\sigma_i^{\mathrm{PEO+F127}} = \sigma_i + \sigma_{+\mathrm{F127}}$ where $i = 1, 2$ and 3 represent the 3 PEO polymers investigated. We choose $\sigma_{+\mathrm{F127}}^{-1}$ based on 6.5 k PEO height measurements, as 6.5 k PEO most resembles F127: $\sigma_{+\mathrm{F127}}^{-1} = (4\ \mathrm{nm})^2$ gives $h_{\mathrm{eq}}^{\mathrm{PEO}} = 12.7$ nm and $h_{\mathrm{eq}}^{\mathrm{PEO+F127}} = 15.1$ nm. The height increase with F127 is therefore within the measured 2–4 nm range and the bare height $h_{\mathrm{eq}}^{\mathrm{PEO}}$ is consistent with electrophoresis-based measurements.

The effective brush heights are summarized in Table S5. We have also verified that small changes to these effective brush parameters do not alter the modeling results of this work significantly.

| Molar mass [g/mol] | PEO units $N_{\mathrm{PEO}}$ | $L^{\mathrm{PEO+F127}}$ [nm] exp. data | $h_{\mathrm{eq}}^{\mathrm{PEO+F127}}$ [nm] used in the model |
|---|---|---|---|
| 6,500 | 148 | 16.6 ± 1.4 | 15.1 |
| 11,000 | 250 | 18.6 ± 1.0 | 24.0 |
| 34,000 | 772 | 33.0 ± 2.7 | 31.4 |

**Table S5:** Retained values for the height of each brush, $h_{\mathrm{eq}}^{\mathrm{PEO+F127}}$, used in the model, and consistency with measured brush heights from electrophoresis experiments, $L^{\mathrm{PEO+F127}}$.

Note that for the DNA coated glass surface, we find that water on DNA-coated glass has a contact angle of $\lesssim 20°$. The thickness of the adsorbed PPO layer of the (PEO-PPO-PEO) F127 on a surface that is so hydrophilic[9] is $\lesssim 1$ nm and thus we can neglect F127 adsorption on glass.

### 2.4.2 Modeling heterogeneous brushes (PEO + ssDNA)

**Modeling ssDNA brushes** For ssDNA, we employ the persistence length $\ell_{\mathrm{DNA}}$ for single stranded DNA calculated following the fits reported in Ref. 29 (and based on experimental data). This includes corrections to the persistence length at different salt concentrations. Most of our experiments are performed in PBS buffer at 140 mM, for which $\ell_{\mathrm{DNA}} = 1.49$ nm.

We now infer the number of DNA segments $N_{\mathrm{DNA}}$ corresponding to the polymer walk and that are used to calculate the brush length. We write $N_{\mathrm{DNA}} = N_0 b_0 / \ell_{DNA}$ where $N_0$ is the number of nucleotides and $b_0 = 0.56$ nm corresponds to the distance between two nucleotides in single-stranded DNA. The value $b_0 = 0.56$ nm was obtained from Ref. 29 and is consistent with the estimate of Ref. 30.

Within the brush model the height of an ssDNA brush is therefore similar to Eq. (S5),

$$h_{\mathrm{eq}}^{\mathrm{DNA}} = \left(\frac{12}{\pi^2}\,\sigma\,\ell_{\mathrm{DNA}}^2\omega_{\mathrm{DNA}}\right)^{1/3} N_{\mathrm{DNA}}. \tag{S7}$$

We make the commonly used simplification $\omega_{\mathrm{DNA}} = \ell_{\mathrm{DNA}}^3$, as reported in Ref. 31.

**ssDNA brush on glass substrate** The DNA density on the glass substrate was calibrated (from an initial model experiment fit – see Main text) to be $\sigma_{\mathrm{glass}}^{-1} = (9.4\ \mathrm{nm})^2$. This value was used throughout all modeling efforts and is in reasonable agreement with an expected slightly lower grafting density on the glass substrate (see Sec. 2.2). Using this value, we find $h_{\mathrm{eq}}^{\mathrm{DNA}} = 10.5$ nm on the bottom glass surface for $N_0 = 60$ base pairs.

**Modeling heterogeneous brushes (PEO + ssDNA) on PS particles** Diblock polymer brushes (here corresponding to PEO + ssDNA) feature very similar properties as single block polymer brushes, provided the mixing energy of both parts is rather small[32]. Since the ssDNA part of our diblock polymers is rather short (number of segments $N_{\mathrm{DNA}} \sim 10$) compared to the PEO part (number of segments $N_{\mathrm{PEO}} > 100$), we model the heterogeneous brush as a homogeneous brush with effective parameters.

The effective brush parameters are $(N_{\text{eff}}, \sigma_{\text{eff}}, \ell_{\text{eff}}, h_{\text{eq}}^{\text{eff}})$ where we take $h_{\text{eq}}^{\text{eff}} = \left(\frac{12}{\pi^2}\sigma_{\text{eff}}\ell_{\text{eff}}^5\right)^{1/3} N_{\text{eff}}$. We use the same density on the grafting surface $\sigma_{\text{eff}} = \sigma$. To ensure conservation of the energy, since the steric energy grows linearly with the number of segments $N$[24], we take the total number of segments to be the sum of the two parts: $N_{\text{eff}} = N_{\text{PEO}} + N_{\text{DNA}}$. Furthermore, we require the height of the effective brush to be the sum of the separate brushes ($h_{\text{eq}}^{\text{eff}} = h_{\text{eq}}^{\text{PEO+F127}} + h_{\text{eq}}^{\text{DNA}}$). This is a reasonable assumption especially considering that the ssDNA part is quite short compared to the PEO part. These choices fully specify the parameters of the effective brush model for the heterogeneous brush.

Calculated heights for the different brushes used in the experiments are presented in Tab. S6 and typically add 8 nm to the brush height. Note that brush heights with ssDNA may not be measured through the method used in Sec. 2.1 as ssDNA would modify the effective charge of the particle, and therefore its mobility, in a very non-trivial way.

| $M_w$ [g/mol] | PEO units $N_{\text{PEO}}$ | DNA bases | $h_{\text{eq}}^{\text{PEO+F127}}$ [nm] theory | $h_{\text{eq}}^{\text{eff}}$ [nm] theory with DNA |
|---|---|---|---|---|
| 6,500 | 148 | 20 | 15.1 | 23.5 |
| 11,000 | 250 | 20 | 24.0 | 32.1 |
| 34,000 | 772 | 20 | 31.4 | 38.2 |

**Table S6:** Brush heights used in the model considering adsorbed F127, without ($h_{\text{eq}}^{\text{PEO+F127}}$) and with ($h_{\text{eq}}^{\text{eff}}$) the DNA strand.

# 3 Model for free energy of interaction of DNA-coated particles

## 3.1 Free energy of interaction

The free energy of interaction of a colloidal particle with a wall (see Fig. S6) decomposes into bulk (gravity) and surface terms as $\phi(h) = \phi_{\text{surf}}(h) + \phi_{\text{grav}}(h)$.

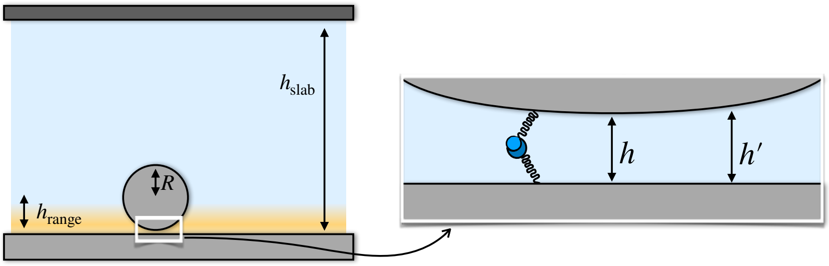


**Figure S6:** Sketch of a typical experimental setup and relevant length-scales

### 3.1.1 Bulk terms: buoyancy

The only bulk term in the free energy is the gravitational buoyancy, which we write as

$$\phi_{\text{grav}}(h) = Gh = mgh \equiv \frac{4}{3}\pi a^3(T)\,\left[\rho_{PS}(T) - \rho_W(T)\right] gh\,, \tag{S8}$$

where $h$ if the distance of closest approach between the sphere and the substrate, as illustrated in Fig. S6. The density of polystyrene $\rho_{PS} = 1.055$ g/cm$^3$ was obtained from the manufacturer. Polystyrene bead sizes range from 1–6 $\mu$m in diameter according to the different experiments, with polydispersity between 1~4%. The thermal expansion of polystyrene was taken into account by using a linear expansion of $-0.000210$ g/cm$^3$/K[33]. Accordingly the radius of the colloid extends to conserve mass as

$a(T) = [a^3(T_0)\rho_{PS}(T_0)/\rho_{PS}(T)]^{1/3}$. The temperature dependence of the radius is taken into account in modeling subsequently, but to simplify notation, the explicit dependence on temperature is dropped.

As the density of water also changes with the temperature, we performed a calibration experiment for the range of temperatures used and with a typical solution and calibrate $\rho_W(T)$ as explained in Table S7 and Fig. S7. A spline fit of degree 1 is then used to calculate the value of the density at any temperature.

| **Temperature** (°$C$) | **Density** g/cm$^3$ |
|---|---|
| 22 | 1.00531 |
| 30 | 1.00306 |
| 40 | 0.99940 |
| 45 | 0.99736 |
| 50 | 0.99511 |
| 55 | 0.99270 |
| 60 | 0.99013 |
| 65 | 0.98676 |
| 70 | 0.98345 |

**Table S7:** Density of water with PBS buffer at 140 mM in 0.3% F127, measured using DMA 45000M density meter.

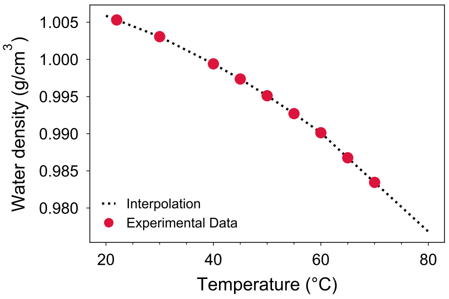


**Figure S7:** Interpolation of the water density in the range of temperatures of interest.

### 3.1.2 Surface terms

Since the interactions are usually very short range (about 10–60 nm) compared to the size of the colloid (typically > 500 nm), we use a Derjaguin approximation to calculate surface contributions to the potential[34]. In brief, consider the surface interaction energy $\varphi(h)$ to only depend on the vertical local distance $h'$ from the substrate to the sphere. Then,

$$\phi_{\text{surf}}(h) = \int_{r(h)}^{\infty} 2\pi\, r(h')\, \varphi(h')\, dr\,, \tag{S9}$$

where $r(h')$ is the radius of a circle (parallel to the planar wall) at height $h'$ from the plane. From simple geometry, one finds $r(h') = a\sqrt{1-(1-h'/a)^2} \simeq \sqrt{2ah'}$ for $h' \ll a$ with $dr = \sqrt{a/2h'}\, dh'$, such that

$$\phi_{\text{surf}}(h) = 2\pi a \int_{h}^{\infty} \varphi(h')\, dh'\,, \tag{S10}$$

which is the usual Derjaguin expression. The Derjaguin approximation is well suited for our system. Polymer brush interactions are modified from their planar form when the size of the brush becomes comparable to the radius of curvature[35]. In our systems, this is never true.

Note that in the case of two interacting spheres of similar radius $a$ one would simply obtain $\phi_{\text{surf}}(h) = \pi a \int_h^\infty \varphi(h')\, dh'$, such that to convert the surface potentials measured here to an interaction between two spheres, one would simply have to divide all surface interactions by a factor 2.

The details of the surface contributions are given in the next section.

Collecting all terms yields

$$\boxed{\phi(h) = \phi_{\text{surf}}(h) + \phi_{\text{grav}}(h) = 2\pi a \int_{h}^{\infty} \varphi(h')\, dh' + \frac{4}{3}\pi a^3 (\rho_{PS} - \rho_W)\, gh.} \tag{S11}$$

## 3.2 From surface free energy to melting curves

A central task of any model of binding of DNA-coated particles is to relate the attractive interaction between DNA-coated surfaces to the temperature, or equivalently, the thermal energy $k_B T$. The binding-unbinding transition between DNA-coated surfaces occurs over a narrow range of temperatures, typically on the order of 1°C, and is generally referred to as "melting". For a generic attractive potential well, one might expect the probability of unbinding to be related to both the width and depth of the potential well. In this section, we develop a quantitative description for these intuitive ideas.

### 3.2.1 Equilibrium considerations and "thermodynamic" melting curve

We consider a particle equilibrated in an aqueous region located between two glass slides, top and bottom, as illustrated in Fig. S6. The particle and bottom glass slide (the substrate) are usually functionalized with complementary ssDNA. The total height of water accessible to the particle is given by $h_{\text{slab}}$ and is typically a few hundred micrometers in an experiment; we will see that its exact value does not play a significant role. One reason is that in addition to the short-range attraction due to DNA binding, the particle, which is about 5% denser than water, is gravitationally weakly bound to the lower substrate, having a gravitational height that is on the order of 120 nm.

**Range of motion at equilibrium.** It is useful to develop a robust quantitative measure of the range that a particle will access within the potential well, one that is readily applied to potentials that may be asymmetric and highly dependent on temperature. To some extent—and we will give more insight as to why—the range of motion is also an indication of the absolute width of the potential.

We start by noting that when the particle sits at height $h$ from the bottom surface, it has free energy $\phi(h)$. The probability that the particle is sitting between $h$ and $h + dh$ is proportional to $e^{-\phi(h)/k_B T} dh$. The Boltzmann factor in this expression is largest near a height $h_{\text{min}}$, where $\phi(h_{\text{min}})$ is at its minimum value, and rapidly decreases as $h$ deviates from $h_{\text{min}}$. We can thus get a measure of the range of motion of the particle simply by integrating $h - h_{\text{min}}$ or $(h - h_{\text{min}})^2$ multiplied by the Boltzmann factor over the range of accessible heights around the potential minimum. In a narrow potential well $h - h_{\text{min}}$ would average out to 0, as the particle is not inclined to move preferentially in one direction, therefore $(h - h_{\text{min}})^2$ is better suited to our purpose here. We therefore propose the range of motion $\delta h$, defined by the integral

$$\boxed{\delta h = \left( \frac{1}{Z} \int_0^{h_{\text{slab}}} (h - h_{\text{min}})^2 e^{-(\phi(h) - \phi_{\text{min}})/k_B T} dh \right)^{1/2}} \,, \tag{S12}$$

where $\phi_{\text{min}} = \phi(h_{\text{min}})$ and $Z = \int_0^{h_{\text{slab}}} e^{-(\phi(h) - \phi_{\text{min}})/k_B T} dh$ is a normalization constant. We note that $\delta h$ has the dimensions of length.

Finally, we explore the value of $\delta h$ at high temperatures, when the binding well is no longer at play. In that case, the potential is mostly given by the linear gravitational contribution $\phi(h) \simeq \phi_{\text{grav}}(h) = Gh$. We write $h_{\text{grav}} = k_B T/G = 124$ nm for our particles at $T = 60.5$°C. Here since $h_{\text{slab}} = 250\ \mu$m $\gg h_{\text{grav}}$, it is easy to calculate $\delta h$ from Eq. (S12) and one obtains $\delta h = \sqrt{2} h_{\text{grav}} \simeq 175$ nm. In practice, we do not obtain $\delta h \simeq 175$ nm at high temperatures but a slightly lower value $\delta h \simeq 140$ nm that is reminiscent of van der Waals interactions (see Sec. 3.5 for a discussion of van der Waals interactions). In Fig. S8 we show that removal of van der Waals interactions from the simulated potential yields indeed $\delta h = \sqrt{2} h_{\text{grav}}$. Next, we show how other statistical measures give information on the probability to be bound.

**Bound and unbound probabilities.** Let's define the thermodynamic probability of the particle to be bound $p_{\text{bound}}^{\text{thermo}}$ as the probability of the particle to be found between the bottom flat surface and the critical height range $h_{\text{range}} = 20\ \text{nm} + h_{\text{min}}$ above which it is considered unbound. In that case

$$p_{\text{bound}}^{\text{thermo}} = \frac{1}{Z} \int_0^{h_{\text{range}}} e^{-\beta\phi(h)} dh \text{ , where } Z = \int_0^{h_{\text{slab}}} e^{-\beta\phi(h)} dh \tag{S13}$$

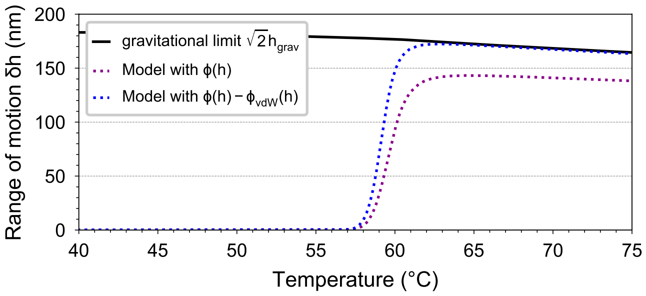


**Figure S8:** Range of motion $\delta h = \langle (h - h_{\min})^2 \rangle_\phi^{1/2}$ around the potential minimum with temperature, calculated with (dashed purple) the full potential $\phi(h)$, (dashed blue) the full potential not including van der Waals attraction $\phi(h) - \phi_{vdW}(h)$ and compared (full black) to the value $\delta h = \sqrt{2}h_{\rm grav}$ that would be obtained for a gravity only potential. Parameters used are the same as those in Fig. 2c of the main paper.

and $p^{\rm thermo}_{\rm unbound} = 1 - p^{\rm thermo}_{\rm bound}$, where $\beta = 1/k_B T$. This definition was used to obtain thermodynamic melting curve in Fig. 2d of the main paper.

While it is quite natural that all particles be bound $p^{\rm thermo}_{\rm bound}(T \ll T_m) = 1$ (respectively $p^{\rm thermo}_{\rm unbound}(T \ll T_m) = 0$) at low temperatures, the value of $p^{\rm thermo}_{\rm bound}(T \gg T_m)$ at high temperatures is not necessarily intuitive as it does not vanish. As shown in Fig. 2d we have obtained $p^{\rm thermo}_{\rm bound}(T \gg T_m) = 1 - p^{\rm thermo}_{\rm bound}(T \gg T_m) \simeq 1 - 0.6 = 0.4$. In fact this remaining effective bound probability is due to gravity, that keeps particles close to the surface with a non-zero probability. If we take at high temperatures $\phi(h) \simeq \phi_{\rm grav}(h) = Gh$ we obtain indeed (again using $h_{\rm grav} \ll h_{\rm slab}$)

$$p^{\rm thermo}_{\rm bound}(T \gg T_m) \simeq 1 - \exp\left(-\frac{h_{\rm range}}{h_{\rm grav}}\right) \simeq 0.4 \tag{S14}$$

since $h_{\min} \simeq 45$ nm.

### 3.2.2 Kinetic considerations and "kinetic" melting curve

**Kinetic unbound probability.** Another way to define the probability of a particle to be bound is a dynamic definition and considers that a particle is bound if within a time frame $\tau$ the particle remained consistently close to the surface. In other words, if at any time during a time frame $\tau$ the particle rose higher than $h_{\rm range}$ then the particle is considered as unbound. We write the unbound probability in that case $p^{\rm kinetic}_{\rm unbound}$. Importantly this definition of melting is dependent on the chosen time frame $\tau$.

This kinetic definition can be mapped to a standard mathematical problem in the following way. In the potential field $\phi(x)$ consider the particle's motion $X(t)$ obeying the overdamped Langevin equation

$$\frac{dX}{dt} = -\frac{D}{k_B T}\partial_x \phi(x) + \sqrt{2D}\,\eta(t) \tag{S15}$$

where $D$ is the diffusion coefficient of the particle and $\eta(t)$ is a white gaussian noise. To map to the experimental kinetic definition of bound, let's consider that the particle is absorbed at $X(t) = h_{\rm range}$. We then have to find the average survival probability for the particle after a time $\tau$. For the sake of simplicity we consider that the particle's trajectory started at the potential minimum. Importantly we notice here that to find theoretically the unbound probability $p^{\rm kinetic}_{\rm unbound}$, one requires the knowledge of the diffusion coefficient $D$.

**Diffusion coefficient.** To compute the kinetic melting curve we use the hindered diffusion of the free particle (parallel to the surface) at height $h \simeq 0.1$ nm above the hydrodynamic floor, $D_\parallel \simeq D_0 h/a$[36,37] where $D_0 = 0.208 \times 10^{-12}$ m$^2$/s is the measured bare diffusion coefficient and $2a = 5$ $\mu$m is the diameter of the particle.

**Numerical implementation.** The probability that at time $t$ the particle is still within the bound region $h \leq h_{\text{range}}$, knowing that the particle started in $x$ is

$$\int_0^{h_{\text{range}}} dh p(h, t|x, 0) \equiv \mathcal{G}(x, t). \tag{S16}$$

The probability we are seeking, *e.g.* to be unbound after time $\tau$ will simply be $p^{\text{kinetic}}_{\text{unbound}} = 1 - G(h_{\text{min}}, \tau)$. It is quite standard to show that $\mathcal{G}(x, t)$ obeys the backward Fokker-Planck equation[38]

$$\partial_t \mathcal{G} = -\frac{D_{||}}{k_B T} \partial_x \phi \, \partial_x \mathcal{G} + D_{||} \, \partial_{xx} \mathcal{G} \tag{S17}$$

with initial conditions $\mathcal{G}(x, 0) = 1$ and boundary conditions $\mathcal{G}(h_{\text{range}}, t) = 0$ characterizing absorption—the particle lifts-off. Finally we use $\partial_x \mathcal{G}(0, t) = 0$ to represent no flux of probability at the bottom wall.

The equations for $\mathcal{G}$ are solved using a fully implicit Euler numerical integration method, respecting upstream advection (according to the sign of the $\partial_x \phi$). Subsequently, $p^{\text{kinetic}}_{\text{unbound}} = 1 - \mathcal{G}(h_{\text{min}}, \tau)$ is calculated and plotted in Fig. 2-d of the main paper.

**Insight from the kinetic melting curve.** The above investigation allows us to reproduce experimental macroscopic melting curves acquired via the ensemble averaged, lift-off criteria. This sheds light on the melting dynamics: DNA pairs unbind and a particle lifts-off with an escape rate of the order of 1 min. The simulation-experiment agreement is somewhat unexpected since the simulated kinetic melting curve shifts with the observation time: specifically, for an observation time twice as long, the melting temperature is slightly lower (by $\sim 1°$C). Though our kinetic model relies on hindered hydrodynamic diffusion, other factors could come into play in such a kinetic description. For example DNA strands could also affect vertical motion,[39–41] and cause the vertical diffusion coefficient of the particle to depend on temperature. These phenomena have yet to be better understood and are beyond the scope of this study.

## 3.3 Surface interactions associated with covalent binding

### 3.3.1 Summary of what this model contains in comparison with previous works

A careful account of the polymer brush, especially of entropic costs due to binding, is central for quantitative description of the interactions, and numerous discussions have arisen concerning modeling assumptions[1,42–48]. To facilitate navigation through our model and comparison with existing theories we recapitulate, similarly as in Ref. 44, the main features of our model compared to existing models on DNA mediated multivalent interactions, see Tables S8 and S9.

| Assumption | Equation | Consistent works | Other works |
|---|---|---|---|
| *Features included in analytical theories* | | | |
| **1. Strand-strand interactions** besides those contained within $\Delta G_{ij}$ (volume exclusion, *etc.*) | Sec. 3.4 | Our work (Unified theory) | All existing models[1,42–48] |
| **2. Bare hybridization energy of DNA** in solution; often using the nearest neighbor model, Sec. 3.3.2. | $\Delta G^0$ | All works | - |
| **3. Effects of strand configurational entropy**, *i.e.* strand structure, especially loss of degrees of freedom upon binding. Often this is done using "Local chemical equilibrium" of interacting strands, implying the strands are dense enough, which is consistent for our very dense brushes. The "Local chemical equilibrium" approximation breaks down at low coating densities, see Appendix B in Ref. 44. | Eq. (S32) and Eq. (S31) | Our work and Ref. 42,48 (analytic expressions, no fitting), Ref. 42,44 (Monte Carlo simulations), Ref. 47 (approximate with fitting) | Ignored by Ref. 1,45. |
| **4. Presence of multiple bond formation**, in essence multivalence | - | all works | |
| **5. Competition for binding partners** or "valence constraint". This allows one to go beyond the so-called "weak-binding" or "Poisson approximation" approximation. In the "Poisson approximation", the strands are indistinguishable and the probability for the particle to be unbound $P_{\text{unbound}} \sim (1-p)^N$ where $p$ is the individual strand probability to be unbound and $N$ is the number of interacting strands. This condition is incorrect in general, as can be seen in our samples where strands are not equivalent, especially not in space, see Fig. 4-D of the main manuscript. We thus obtain a more accurate estimate of both the number of bonds and the free energy of interaction. | Eq. (S22) | Our work and Ref. 42–44; Ref.[45] evaluates the partition function with Monte Carlo data, but still using "local mass conservation", see 6. | Ref. 1,47–49, done with the "Poisson approximation". |
| **6. Global mass conservation** of sticky ends in bound and unbound strands, implying the system is at equilibrium. In contrast, "local mass conservation" implies $C_a(\boldsymbol{r}) \simeq C_a^0(\boldsymbol{r}) - C_{ab}(\boldsymbol{r})$ where $C_a^0(\boldsymbol{r})$ is the initial concentration of active type $a$ sites when no binding occurs (and similarly for type $b$). This is not justified in general and introduces an "effective out-of-equilibrium" condition which is not expected as in equilibrium bound and unbound sticky ends can relax over the separation distance. | Eq. (S20) | Our work and Ref. 42–44 | Ref. 1,45 uses "local mass conservation" |

**Table S8:** Main assumptions made in the model and comparison with existing works

| Assumption | Equation | Consistent works | Other works |
|---|---|---|---|
| *Detailed features included in analytical theories* | | | |
| **1.** Detailed brush structure accounting for heterogeneous structure (linker+DNA) | Sec. 2.3 | Our work | All existing models [1,42–47] |
| **2.** Acknowledgment of coating design differences (or asymmetry) between binding surfaces (particle and surface or particle and particle) | Eq. (S27) and Sec. 3.4.2 | Our work and Ref. 1,42,43 | Many other existing models [1,47] |
| **3.** Coating geometry in the form of a brush (not a mushroom coil) | Eq. (S33) for brushes Sec. 3.4.2 | Our work | All other works explore mushroom coils |
| *Main implicit approximations in analytical theories* | | | |
| **1.** No further interactions between colloids and strand besides excluded volume interactions. | none | all existing works including ours | none. |
| **2.** DNA strands constitute ideal solutes. This underlines that the chemical equilibrium may be written for concentrations or that the chemical potential of each species may be expressed as $\mu_i \simeq \mu_i^0 + k_B T \ln C_i$ where $\mu_i^0$ is the chemical potential at infinite dilution and $C_i$ is the concentration of species $i$, | Eq. (S31) | all existing works including ours | none. |

**Table S9:** Detailed assumptions made in the model and comparison with existing works

### 3.3.2 Free energy of hydrogen binding in solution

**Free energy calculations** The value of the enthalpy $\Delta H^0$ and entropy $\Delta S^0$ of hydrogen binding for the strands used in this study is derived from the unified nearest neighbor model of SantaLucia[50], giving a value for the free energy of hydrogen binding

$$\Delta G^0 = \Delta H^0 - T\Delta S^0. \tag{S18}$$

The values are reported in Table S10. The values we obtained are exactly equal to that predicted from Ref. 51, using the DinaMelt software from Unafold. This is coherent as DinaMelt relies on the unified nearest neighbor model of Ref. 50. We discuss the effect of possible uncertainties on the value of these experimental parameters in Sec. 5.2.1.

| **DNA sequence (from 5′ to 3′)** | $\Delta H^0$ **in** kJ/mol **and** (kcal/mol) | $\Delta S^0$ **in** J/mol/K **and** (cal/mol/K) | $\Delta G^0$ **at** 22°C **in** $k_BT$ | $T_m^{ab} = \frac{\Delta H^0}{\Delta S^0}$ **in** °C |
|---|---|---|---|---|
| ($A^6/B^6$) ACCGCA | −170.3 (−40.6) | −468.7 (−112) | −6.0 | 90.2 |
| ($A^5/B^5$) GACGC | −154.0 (−36.8) | −438.1 (−104.7) | −5.9 | 78.4 |
| ($A^4/B^4$) GCAG | −108.4 (−25.9) | −317.4 (−76) | −1.5 | 68.3 |

**Table S10:** Hydrogen binding for the DNA strands used in this study, as calculated using the model of Ref. 50, at a salt concentration $c_0$ = 140 mM.

### 3.3.3 Entropic effects due to multivalent interactions

DNA strands may bind to a number of other nearby strands. When binding, the strands lose a number of degrees a freedom, thereby resulting in an entropic cost to the binding energy. Furthermore, enumerating the number of possible bound configurations has to be done carefully, so as to account for competition between binding partners. To account for all of these effects, our approach closely follows that of Refs. 42 and 43, with a number of important additions for our system, including that of a polymer brush model in a mean-field theory.

In this section we consider in general the interaction between two coated surfaces, one referred to as the "top" surface, and the other as the "bottom" one. For applications to our experimental setup, the "top" surface corresponds to the particle's surface and the "bottom" one to the glass substrate.

**Competition for binding partners.** The goal of this section is to find a mean-field description to account for competition for binding partners in the binding potential. We start by considering the binding partners in a discrete way. Consider a typical area $\mathcal{A}$ where there are $N_\mathrm{t} = \mathcal{A}\sigma_\mathrm{t}^\mathrm{sticky}$ sticky DNA on the top surface that can potentially bind to $N_\mathrm{b} = \mathcal{A}\sigma_\mathrm{b}^\mathrm{sticky}$ sticky DNA on the bottom surface. Note that on the top surface where F127 adsorption is considered, $\sigma_\mathrm{t}^\mathrm{sticky}$ corresponds to the density $\sigma$ of grafted PEO (with sticky ends) and not to the corrected density for F127 adsorption $\sigma^{+\mathrm{F127}}$. On the bottom surface, all end points are sticky and therefore $\sigma_\mathrm{b}^\mathrm{sticky} = \sigma_\mathrm{glass}$.

**Assumption 1: Localized interactions** Consider all possible DNA tips indexed by $i$ in this $N_\mathrm{t}+N_\mathrm{b}$ ensemble. Consider that another tip $j$ is complementary with $i$. Then we consider that $i$ can bind to $j$ with a free energy change $\Delta G_{ij}$ (we will be more specific as to what goes into $\Delta G_{ij}$ in the following paragraph on lost degrees of freedom upon binding). On the whole, this explicitly assumes that when the ligands are unbound, they do not interact with each other. If $i$ and $j$ are not complementary $\Delta G_{ij} = \infty$. To some extent, this setting may be thought of as an "ideal" approximation where the bonds only interact with $\Delta G_{ij}$ if they are localized on top of each other. In reality, the bonds interact over a characteristic lengthscale corresponding to some interaction potential that is not infinitesimally localized in space. However, here it is a reasonable coarse-graining approximation since the sticky sequence is generally much smaller ($\sim$ 1 nm) than the strand ($\sim$ 10 nm) and hence the sticky end is in "low density" compared to the rest of the strand – see also Ref. 42.

**Working conditions: Global mass conservation.** We now seek to express the probability $p_{ij}$ that the bond $i$-$j$ is formed, relative to the probabilities $p_i$ and $p_j$ that each sticky end $i$ and $j$ is unbound. It is clear that, considering a tip $i$, $i$ is either unbound or in a bond with any tip $j$ in the complementary ensemble of tips for $i$, $\Omega_i$ such that

$$1 = p_i + \sum_{j\in\Omega_i} p_{ij}. \tag{S19}$$

Eq. (S19) represents a global conservation of mass relation, that bears no approximations. We can write its mean-field version, in our case, for 2 complementary strand types, $a$ and $b$, in space. Let $P_a(\boldsymbol{r})$ be the probability of finding a strand of type $a$ unbound at location $\boldsymbol{r}$ in space, and $P_{ab}(\boldsymbol{r})$ that it is bound with a strand of type $b$. Then simply

$$1 = \int d\boldsymbol{r}\,(P_a(\boldsymbol{r}) + P_{ab}(\boldsymbol{r})) \tag{S20}$$

and similarly for $b$. Both conservation equations Eqns. (S19-S19) are foundational in Ref. 42,43. In contrast, in Ref. 1, a local mass conservation equation is used (assuming essentially that $P_a(\boldsymbol{r})+P_{ab}(\boldsymbol{r}) \equiv P_a^0(\boldsymbol{r})$, where $P_a^0(\boldsymbol{r})$ is a set quantity), which induces an effective out-of-equilibrium constraint as strands are not allowed to relax within the gap (see Appendix B of Ref. 42 and Table S8).

**Assumption 2: Many possible binding partners** If $p_{-i,-j}$ is the probability that both $i$ and $j$ are unbound, then we simply have the Boltzmann law $p_{ij} = p_{-i,-j}e^{-\Delta G_{ij}}$. Furthermore, if $i$ and $j$ both have many possible binding partners, which is the case at reasonably intermediate to high coating densities, we can assume that the binding of $i$ and $j$ are uncorrelated events and hence $p_{-i,-j} \simeq p_i p_j$. Finally we obtain

$$p_{ij} = p_i p_j e^{-\Delta G_{ij}}. \tag{S21}$$

Note, that this approximation is inaccurate if only 1 strand interacts with another, see Appendix A in Ref. 44. Therefore, this approximation should not be used for very low density samples. This is not the case in our samples that are very dense, and that have always more than one binding partner. In general one might argue that in such surface/surface settings (colloid on a surface or colloid on another colloid), the particles diffuse slightly relative to one another, therefore always seeing an effective landscape of complementary strands. Hence, it is possible that any colloid strand sees many possible binding partners in time, if not in space, even down to somewhat low densities.

**Main result: Binding including competition for binding partners.** Using a typical replica method (we do not report the steps leading to this equation here but they are carefully laid out in Ref. 43), one can show that the free energy for binding can be written as

$$\beta\varphi_{\text{bind,discrete}} = \sum_{i<j} p_{ij}\beta\Delta G_{ij} + \sum_i \left(1 - \sum_j p_{ij}\right) \ln\left(1 - \sum_k p_{ik}\right) + \sum_{i<j} p_{ij}\ln p_{ij} + \sum_{i<j} p_{ij}. \tag{S22}$$

Note that this equation is essential to account for competition between binding partners and allows one to accurately estimate the number of bonds in the system. In comparison, approaches based on the so-called "Poisson approximation"[1,47,48] overestimate the number of bonds and the free energy[43] – see also Table S8 and following paragraph.

We now apply Eq. (S22) to our system. If $i$ and $j$ are on opposite surfaces, they are complementary strands and we expect $p_{ij} > 0$. If $i$ and $j$ are on the same surface, they are not complementary and thus $p_{ij} = 0$. Furthermore, since our system is composed of polymer brushes, we expect that bonds occur mostly along the vertical direction (a polymer can not bend much horizontally to find many other partners). The relevant area available for binding is thus typically $\mathcal{A} \sim 1/\sigma$; in this small area, it is therefore natural to make the mean-field approximation that functions do not depend much on the relative location of $i$ and $j$ and we can assume $p_{ij} = p$ is uniform and $\Delta G_{ij} = \Delta G_{ab}$, when $i$ and $j$ are on opposite surfaces. Under these conditions, we obtain

$$\beta\varphi_{\text{bind,discrete}} = N_{\text{t}}N_{\text{b}}p\left(\beta\Delta G_{ab} + 1\right) + N_{\text{t}}\left(1 - N_{\text{b}}p\right)\ln\left(1 - N_{\text{b}}p\right) + N_{\text{b}}\left(1 - N_{\text{t}}p\right)\ln\left(1 - N_{\text{t}}p\right) + N_{\text{t}}N_{\text{b}}p\ln p. \tag{S23}$$

**Comparison with the "Poisson approximation"** In contrast, if one completely ignores competition for binding partners, then all tethers can be considered as equivalent and the partition function simply writes $Z = (1 + e^{-\beta\Delta G_{ab}})^{N_t N_b}$. Hence the free energy for binding is

$$\beta\varphi_{\text{bind,Poisson}} = -\ln Z = -N_t N_b \ln(1 + e^{-\beta\Delta G_{ab}}). \tag{S24}$$

Performing a Taylor expansion around the "weak binding regime" where $e^{-\beta\Delta G_{ab}} \ll 1$ (each individual reaction is therefore not so likely, $\beta\Delta G_{ab} \gtrsim 1$), we find

$$\beta\varphi_{\text{bind,Poisson}} \simeq -\ln Z = -N_t N_b \left(e^{-\beta\Delta G_{ab}} - \frac{e^{-2\beta\Delta G_{ab}}}{2} + ...\right). \tag{S25}$$

Using Eq. (S19) and Eq. (S21), we obtain $p = \frac{\sqrt{1+4e^{-\beta\Delta G_{ab}}}-1}{2e^{-\beta\Delta G_{ab}}}$. Such that the expression including competition for binding partners Eq. (S23) may also be expanded[43]

$$\beta\varphi_{\text{bind,discrete}} = N_{\text{t}}N_{\text{b}}\left(e^{-\beta\Delta G_{ab}} - e^{-2\beta\Delta G_{ab}} + ...\right). \tag{S26}$$

We observe, similarly as in Ref. 43, that both expressions are quite distinct in the weak binding regime. We will discuss these discrepancies in more detail in Sec. 5.3.2.

**Mean-field approximation** In general the bottom and the top surfaces do not have an equal density of sticky ends. For the purpose of the derivation we suppose here that the bottom surface is the denser one (but the reasoning is similar if the top surface is the denser one).

We thus now consider an elementary area say $\mathcal{A} = 1/\sigma_{\mathrm{b}}^{\mathrm{sticky}}$. Let us write $N_t p \equiv f$ the "fraction of bound pairs" on the top surface. $f$ is a real number anywhere between 0 and $\alpha = \sigma_{\mathrm{t}}^{\mathrm{sticky}}/\sigma_{\mathrm{b}}^{\mathrm{sticky}} = N_t/N_b < 1$, and thus $p = f/\alpha$. We can then write the free energy per unit area (multiplying by $\sigma_{\mathrm{b}}^{\mathrm{sticky}}$)

$$\beta\varphi_{\mathrm{bind}} = \sigma_{\mathrm{b}}^{\mathrm{sticky}}\left[f\left(\beta\Delta G_{ab}+1\right)+\alpha\left(1-\frac{f}{\alpha}\right)\ln\left(1-\frac{f}{\alpha}\right)+(1-f)\ln(1-f)+f\ln f-f\ln\alpha\right], \quad \text{(S27)}$$

and similarly if the top surface is the denser surface. Note that although this expression was obtained requiring that binding happened typically binding happens in a form of one on one, it is still a mean-field result, averaging over the pairing possibilities.

In the case of symmetric top and bottom coverage ($\alpha = 1$) we obtain a simpler expression

$$\beta\varphi_{\mathrm{bind}} = f\left(\beta\Delta G_{ab}+1\right)+2\left(1-f\right)\ln\left(1-f\right)+f\ln f. \quad \text{(S28)}$$

**"No brush modification" model** In systems where one can assume *a priori* that the fraction of bound tethers is independent of other physical mechanisms (for example if the fraction of bound tethers does not change significantly the brush's elastic properties and hence steric repulsion – which is *a priori* not the case in our dense systems with important strand-strand interactions) then one can obtain $f$ self-consistently. The actual value of $f$ is the one that minimizes $\varphi_{\mathrm{bind}}$ such that $\partial_f\varphi_{\mathrm{bind}}(f) = 0$. For symmetric layers one would obtain

$$f = \frac{1}{J}\left[(1+J)-\sqrt{1+2J}\right] \quad \text{(S29)}$$

where $J = 2e^{-\Delta G_{ab}}$ is representative of the coupling constant. Eq. (S29) is exactly what is obtained in Ref. 42 using another mean-field approach. This justifies further the final expression obtained to account for competition between binding partners, Eq. (S27). Note that here, we will not rely on Eq. (S29), since we expect the fraction of bound tethers to affect other components of the free energy (in particular the elastic properties and detailed structure of the brush, and hence steric repulsion) as well.

**Lost degrees of freedom upon binding.** Our follow-up goal is to identify what enters in the effective hybridization energy $\Delta G_{ab}$ and obtain the binding reaction constant $K_{ab} = e^{-\Delta G_{ab}}$ for hybridization, considering that the DNA strands are not just freely diffusing in a bath. $\Delta G_{ab}$ is thus the free energy associated with the binding of an individual pair of DNA strands that are attached to opposite surfaces. It must thus account both for bare hybridization energy and entropic (or configurational) costs associated with binding: upon binding the strands lose degrees of freedom.

In the following models we assume that the strands still interact "ideally", with the interaction energy $\Delta G_{ab}^{0}$ (the "bare" hybridization energy, defined in Sec. 3.3.2) when their sticky ends coincide and none otherwise, that is to say that configurations of strands have the same statistical weight whether they are bound or unbound. This is a consequence of the "Main Assumption 1: Localized interactions" above.

In a mean-field approximation (*i.e.* integrating over all possible relative locations $\boldsymbol{r}_{ab}$ of strands of type $a$ and $b$), the binding reaction constant is simply expressed by counting the accessible configurations $q_i$ in each of the states (unbound $a$ and $b$ or bound $ab$)

$$K_{ab} = e^{-\beta\Delta G_{ab}^{0}}\int_S \sigma_{\mathrm{sticky}} d^2 r_{ab} e^{-\beta\Delta G_{ab}^{(\mathrm{conf})}} = e^{-\beta\Delta G_{ab}^{0}}\int_S \sigma_{\mathrm{sticky}} d^2 r_{ab}\frac{\int_{\Omega_S} d\boldsymbol{r}\, q_{ab}(\boldsymbol{r})}{\int_{\Omega_S} d\boldsymbol{r} q_a(\boldsymbol{r})\int_{\Omega_S} d\boldsymbol{r} q_b(\boldsymbol{r})} \quad \text{(S30)}$$

where the integrals are done over the surfaces in contact, $\mathcal{S}$, and the separation volume between the plates, $\Omega_{\mathcal{S}}$.

We will now use an assumption to simplify further Eq. (S30).

**Assumption 1: "Ideal solute".** If the DNA sticky ends are assumed to be ideal solutes, we can approximate the accessible configurations by the probability densities $q_a(\boldsymbol{r}) = P_a^{(0)}(\boldsymbol{r})$ (and similarly for $b$) where $P_a^{(0)}(\boldsymbol{r})$ is the probability before hybridization happens. Note the subtle difference between $P_a^{(0)}(\boldsymbol{r})$ and $P_a(\boldsymbol{r})$, where the latter one is the unbound probability distribution considering that hybridization can happen.

**Assumption 2: "local chemical equilibrium".** The difficult task is now to express the bound configurations $q_{ab}(\boldsymbol{r})$. We can write it according to a "local chemical equilibrium" of making the bond at location $\boldsymbol{r}$ within the gap

$$q_{ab}(\boldsymbol{r}) = \frac{q_a(\boldsymbol{r}) q_b(\boldsymbol{r}_{ab} - \boldsymbol{r})}{\rho_0} \tag{S31}$$

Here, Eq. (S31), also used in a number of other works [1,42,48] makes the assumption that locally, one can write a classical equilibrium condition on the reaction as if the DNA strands were behaving as ideal solutes. Importantly, this is only true if the strands are in a sufficiently dense regime, which is expected in our systems [44] (see also Table S8 and S9).

**Result: Expression for the effective reaction constant.** Wrapping up these assumptions we obtain the binding reaction constant between two strands with respect to the probability distributions (or concentrations) of the unbound strands considering they do not interact

$$K_{ab} = \frac{\sigma^{\text{sticky}} e^{-\beta \Delta G^0_{ab}}}{\rho_0} \frac{\iint d^2\boldsymbol{r}_{ab} \int_0^{h_0} \iint dx\, dy\, dz\, P_a^{(0)}(\boldsymbol{r})\, P_b^{(0)}(\boldsymbol{r}_{ab} - \boldsymbol{r})}{\int_0^{h_0} \iint dx\, dy\, dz\, P_a^{(0)}(\boldsymbol{r}) \int_0^{h_0} \iint dx\, dy\, dz\, P_b^{(0)}(\boldsymbol{r})} \tag{S32}$$

where $\sigma^{\text{sticky}}$ is the areal density of sticky ends ($\sigma^{\text{sticky}} = \sigma$ if the percentage of complementary DNA inserted during the clicking phase is 100%, we will come back to what the appropriate value of $\sigma^{\text{sticky}}$ is for coatings that are not of equal density on both surfaces – termed here asymmetric coatings).

**Application to polymer brushes (Milner-Witten-Cates).** Finally, we now detail how to apply the above expressions in the case of polymer brushes, as is relevant for our system. For polymer brushes, it is consistent to consider that the brush properties are uniform along the surfaces ($x, y$ directions) and that the brushes bind mainly along the vertical direction (within the mean field approximation). We thus obtain from Eq. (S32) the simplified binding reaction constant

$$K_{ab} = \frac{\sigma_{\text{sticky}} e^{-\beta \Delta G^0_{ab}}}{\rho_0} \int_0^{h_0} dz P_a^{(0)}(z) P_b^{(0)}(h_0 - z) \tag{S33}$$

where the integral represents the probability that the strand tips are located at the same position. Note that for asymmetric polymer brush layers, $\sigma_{\text{sticky}}$ in Eq. (S33) should be set to the maximal sticky density between $\sigma^t_{\text{sticky}}$ and $\sigma^b_{\text{sticky}}$. This choice together with Eq. (S27) guarantees that we recover the self-consistent results that can be obtained with Ref. 42 using another approach (if the "no brush modification" assumption is made, which is something we will not do in the following).

In the standard Milner-Witten-Cates theory, when brought into close contact the brushes do not interdigitate and do not overlap. For the purposes of binding, interdigitation is crucial to guarantee that some sticky ends bind. In realistic situations, interdigitation is obviously happening especially since the brush is softer near the tip, facilitating interdigitation at the tip. For the purpose of evaluating Eq. (S33),

we will thus assume that the brushes do partially interdigitate. The typical interdigitation length is (Eq. (111) of Ref. 52)

$$\ell_{\rm int} \simeq \ell N^{0.51} (\sigma \ell^2)^{-0.15} (h/\ell)^{-0.18}. \tag{S34}$$

To evaluate $\ell_{\rm int}$ for facing (asymmetric) brushes we take average brush parameters ($\ell = (\ell_t + \ell_b)/2$ and similarly for $N$ and $\sigma$). For 34k PEO, we find $\ell_{\rm int} = 3 - 15$ nm according to the degree of compression of the brushes due to the separation distance $h$ decreasing (15 nm corresponds to the maximum compression considered with the brush being compressed at 30% it's original height).

When the brush is compressed ($h \leq h_{t,\rm eq} + h_{b,\rm eq}$), the brush heights $h_t$ and $h_b$ are smaller than the equilibrium heights $h_{t,\rm eq}$ and $h_{b,\rm eq}$, (for a symmetric brush, $h_t = h_b$), and they overlap by a maximum of $\ell_{\max} = (h_{t,\rm eq} + h_{b,\rm eq}) - h$. We thus take as the actual interdigitation length the minimum value between $\ell_{\max}$, $\ell_{\rm int}$, $h_t$ and $h_b$. The effective interdigitation length can thus be written as

$$h_{\rm bond} = \min\left(\ell_{\max}, \ell_{\rm int}, h_t, h_b\right) \tag{S35}$$

and is typically $h_{\rm bond} \simeq 0 - 10$ nm. The effective height of each layer is thus $h_{i,\rm eff} = h_i + h_{\rm bond}$, with $h_t + h_b = h$.

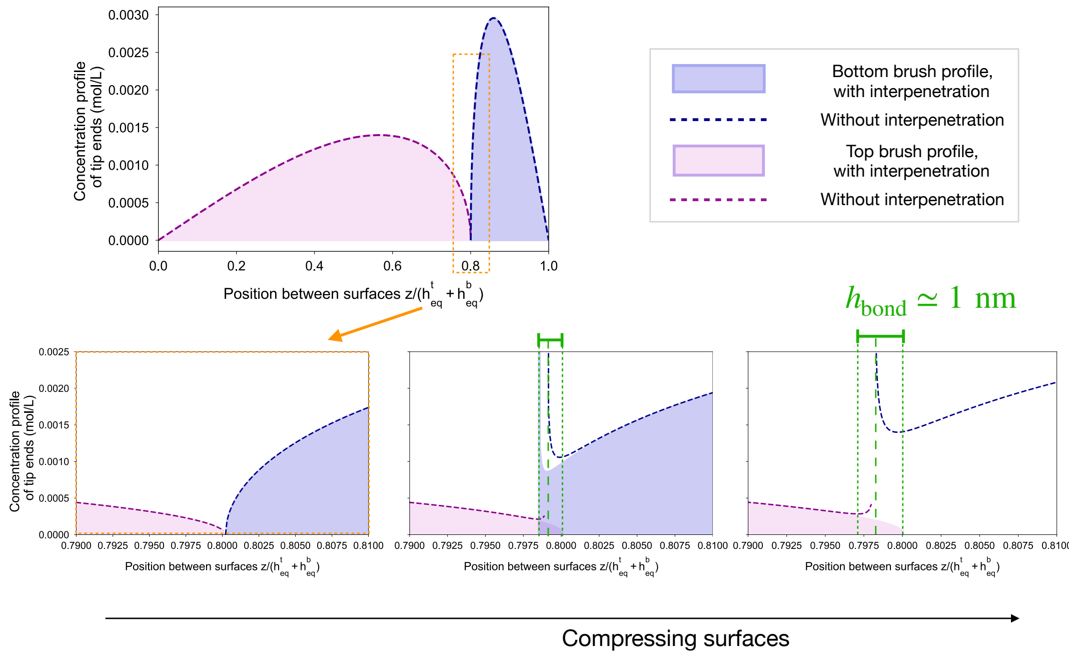


**Figure S9:** Concentration Profile of sticky ends according to Eq. (S36) for 2 facing brush layers. Here model parameters are that of the experimental system of Fig. 1 and 2 of the main paper, namely a top colloid with 34k PEO and 20 DNA nucleotides including 6 sticky ends, and density $\sigma = 1/(6.9\ \mathrm{nm}^2)$; while the bottom layer is 60 DNA nucleotides including the 6 complementary ends, and density $\sigma = 1/(9.4\ \mathrm{nm}^2)$. Graphs represent concentration profiles on planar surfaces. The bottom graphs represent zoomed portions of the concentration profiles at increasing spacing compression, from the rest distance $h^t_{\rm eq} + h^b_{\rm eq} \simeq 48$ nm. The interdigitation $h_{\rm bond}$ length is highlighted on each graph. As the bottom layer is quite thin ($h^b_{\rm eq}$) even small compressions of the layer results in tip accumulation at the interface. This feature was shown consistently in Ref. 24.

To quantify further brush interdigitation, we must specify the distribution of sticky ends for each brush $P_i^{(0)}(z)$. Consistently with the calculation of steric repulsion, we take an expression of $P_i^{(0)}(z)$ close to its equilibrium distribution[24] as

$$P_i^{(0)}(z) = \frac{3}{2h_{i,\rm eq}^3}\left[2z\sqrt{h_{i,\rm eff}^2 - z^2} + \left(\left[\frac{2}{3}\frac{h_{i,\rm eq}^3}{h_{\max}} + \frac{h_{i,\rm eff}^2}{3}\right] - h_{i,\rm eff}^2\right)\frac{z}{\sqrt{h_{i,\rm eff}^2 - z^2}}\right] \tag{S36}$$

for a height $z < h_{i,\rm eq}$, where $h_{i,\rm eq}$ is the equilibrium height of the brush, and $P_i(z \geq h_{i,\rm eq}) = 0$. This means that $K_{ab}$ is essentially 0 when the brushes are far from each other, because they do not intersect, so their

reaction probability is 0. For intermediate values of the distance, $K_{ab}$ is now relevant. In particular, the entropic interaction penalty is quite strong. The effective free energy of interaction $\Delta G_{\mathrm{ab}} = -k_B T \ln(K_{ab})$ is typically 6–8 $k_B T$ higher than $\Delta G^0_{\mathrm{ab}}$.

We have checked that our results only marginally depend on the choice of this interdigitation framework. For example a constant interdigitation length of 2–5 nm yielded very similar results, both quantitatively and qualitatively.

To illustrate interdigitation as modeled here, we show typical tip end concentration profiles in Fig. S9, using characteristic experimental parameters from the main paper.

## 3.4 Unified theory with steric repulsion and binding

Treating steric repulsion and binding energies separately is expected to only be valid in a "weak binding" regime where the fact that a few bounds are formed between opposing brushes does not affect significantly the elasticity of the brush. This is due to the fact that the configuration of the brush is optimized to minimize steric repulsion energy but before binding is considered.

Moreover, as we have seen earlier, writing down the binding part of the energy requires one to make several assumptions on how the strands are going to bind. Here we develop a theory to directly express both steric repulsion and binding, which resolves both issues.

### 3.4.1 Symmetric brushes

**Set up of the problem** We consider first the case of symmetric opposing grafted brushes – see Fig. S10.. The density of polymers is $\sigma$ on each side, and the brushes are made of $N$ chain segments of length $\ell$. At a given separation $h$ of the plates, the brushes may bind by their end tips. Since the system is explored at equilibrium, we expect only the lowest energy configuration of the brushes to significantly contribute to the overall energy. Without loss of generality, because the problem is symmetric, we may assume that the lowest energy configuration corresponds to binding occurring only at the center plane in $h/2$. Small fluctuations around equilibrium are only considered to calculate the effective binding energy in Eq. (S33) and do not play a large role as seen in the previous section.

We further assume that the bound polymers extend slightly beyond the brush layer of height $L$ (see Fig. S10). This is expected since the added stretching energy should be compensated by binding with an opposing partner. We will check *a posteriori* that the theory we derive does predict a positive extension beyond the brush layer. Therefore the bound polymers extend until $H = h/2$. We assume that a fraction $f$ of polymers are bound and $(1 - f)$ are unbound. Also, the number of segments beyond the layer (in the gap) for a bound polymer is $P$ and only $N - P$ segments are within the layer.

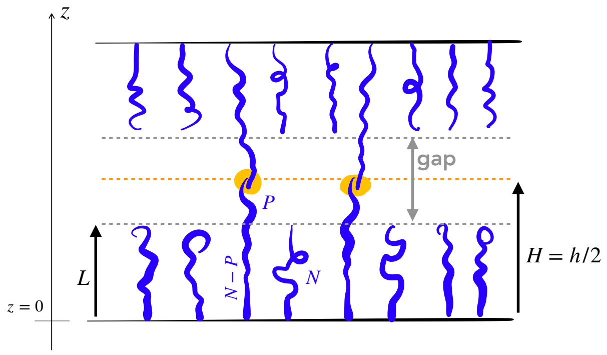


**Figure S10:** Combined theory setup to account both for steric repulsion and binding of polymer brush layers simultaneously

In accordance with Milner, Witten, and Cates, and also with Meng and Russel[53], we employ an analogy between the position $z(n)$ of a polymer brush (bound or unbound) with respect to the segment index $n$ and position $x(t)$ in an oscillator problem. Therefore $n$ is analogous to time in the oscillator problem, and the stretching of the polymer segments $dz/dn$ is analogous to velocity.

We will further write $\varphi(z)$ the distribution of monomers in the layer. Note that this distribution can be decomposed between the bound and unbound polymers as $\varphi(z) = \varphi_b(z)+\varphi_u(z)$. The unbound distribution monomers only contributes for $0 < z < L$ while the bound distribution contributes for $0 < z < H$.

When the layers are separated by a large distance, polymers do not bind and they assume their equilibrium height $H = L = h_{\text{eq}} = \left(\frac{12}{\pi^2}\sigma\ell^2\omega\right)^{1/3} N$ as seen above in the standard Milner, Witten Cates formalism.

**Free energy** There are three components in the free energy $f_{\text{tot}}$ (per unit surface) of **one** brush (we here focus on the free energy of one side and not of both):

1. A configurational entropy term due to stretching of the chains. Each segment is stretched by $dz/dn$ and the stretching energy is analogous to a kinetic energy $\frac{1}{2}(dz/dn)^2$. All segments of the chain contribute this amount of kinetic energy and thus the surface stretching energy of a chain is

$$\varphi_{\text{stretch, single chain}} = \frac{1}{\sigma}\int_0^N \frac{1}{2}\left(\frac{dz}{dn}\right)^2 dn \tag{S37}$$

While in the standard MWC theory the stretching energy does not depend on the tip end position $z_0$ of the chain within the brush, it is not the case for the binding brush, which has a more complex geometry. We thus expand the stretching energy to differentiate between bound and unbound brushes, and to integrate over the different tip end positions $z_0$. Explicitly, this is written as

$$\begin{aligned}\varphi_{\text{stretch}} =& \frac{1}{\sigma}\int_0^L \epsilon_u(z_0)\int_0^N \frac{1}{2}\left(\frac{dz_u}{dn}\right)^2 dn\,dz_0\\ &+\frac{1}{\sigma}\int_0^H \epsilon_b(z_0)\int_0^N \frac{1}{2}\left(\frac{dz_b}{dn}\right)^2 dn\,dz_0\end{aligned} \tag{S38}$$

where $\epsilon_i(z_0)$ are the distributions of tip end positions of the unbound and bound polymers, such that $\int \epsilon_b(z_0)dz_0 = \sigma f$ and $\int \epsilon_u(z_0)dz_0 = \sigma(1-f)$. Those expressions will simplify further on.

2. An excluded volume term due to the fact that segments must avoid each other. In the mean-field approximation the excluded volume interaction is proportional to the square of the segment density, with a proportionality constant determined by the segmental excluded volume $\omega$:

$$\varphi_{\text{excluded}} = \frac{\omega}{2\sigma}\int_0^H \varphi^2(z)\,dz = \frac{-1}{2\sigma}\int_0^H \varphi(z)\,U(z)\,dz \tag{S39}$$

where we stress that the segment density has contributions from both bound and unbound polymers.

3. A free energy contribution associated with binding polymers. The contributions to this free energy are, as highlighted above, numerous and subtle. We report the expression of the binding energy here

$$\beta\varphi_{\text{bind}} = f\,(\beta\Delta G_{ab}+1) + \alpha\left(1-\frac{f}{\alpha}\right)\ln\left(1-\frac{f}{\alpha}\right) + (1-f)\ln(1-f) + f\ln f - f\ln\alpha\,, \tag{S40}$$

and refer to the text above for notational details. Here notice that we have omitted multiplication by density as for simplicity we consider for now the energy of a unit area.

**Trajectories of the polymer brushes** We need to minimize the free energy with respect to the parameters of the problems. The free parameters to be determined are $P$ (the number of units of each brush in the gap), $f$ (the fraction of bound brushes) and $L$ the length of the unbound brush. We are missing a few constraints on the parameters to obtain these values. To find the constraints, it is necessary to write a few

more equations on the expected shapes of the different strands. By analogy to the harmonic oscillator, the potential must be parabolic for all free (unbound) chains to reach the wall in an equal number of segments:

$$U(z) = -(A - Bz^2) = -\omega\varphi(z) \tag{S41}$$

Within the parabolic profile, the equation of motion for the nth segment on a chain is

$$\frac{d^2z}{dn^2} = -\frac{dU}{dz} = -2Bz^2 \tag{S42}$$

regardless of whether the chain is bound or unbound. The potential is the same for bound and unbound chains within the layer. The boundary conditions for the free chains are that:

- the velocity/tension of the tip of the free chain vanishes: $\frac{dz_u}{dn}(n = N) = 0$
- the free chain starts at the wall $z_u(n = 0) = 0$
- the free chain achieves its final position far from the wall in exactly $N$ segments $z_0 = z_u(n = N)$

solving the equation of motion yields

$$z_u(n) = A_u \cos(\sqrt{2B}n) + B_u \sin(\sqrt{2B}n) \tag{S43}$$

and $z_u(n = 0) = 0$ sets $A_u = 0$ and the requirement of vanishing velocity imposes $B = \frac{\pi^2}{8N^2}$. Finally we have

$$z_u(n) = z_0 \sin\left(\frac{\pi n}{2N}\right) \tag{S44}$$

Now for the bound chains the boundary conditions are that

- the bound chain starts at the wall $z_u(n = 0) = 0$
- After exactly $N - P$ segments the bound polymer enters the gap, $z_b(n = N - P) = L$
- To ensure stability of the gap, the tension in the gap is constant equal to $\frac{H-L}{P}$ and it is also continuous to that at the edge of the layer, such that $\frac{dz_b}{dn}(n = N - P) = \frac{H - L}{P}$

such that we obtain first that

$$z_b(n) = \frac{L}{\cos\left(\frac{\pi P}{2N}\right)} \sin\left(\frac{\pi n}{2N}\right) \tag{S45}$$

and then the stability condition

$$\frac{H - L}{L} = \frac{\pi P}{2N} \tan\left(\frac{\pi P}{2N}\right) \tag{S46}$$

yielding

$$\boxed{L(P) = \frac{H}{1 + \frac{\pi P}{2N} \tan\left(\frac{\pi P}{2N}\right)}} \tag{S47}$$

that determines for example $L$ as a function of the number of segments in the gap $P$.

**Density of the brushes** Having the trajectories of all brushes we now turn to computing the density of brushes. The segment density at position $z$ has contributions from all chains that end at a distance $z_0 > z$ and started at $z$ with stretching $dz/dn$ such that

$$\varphi(z) = \int_z^H dz_0 \epsilon(z_0) \left| \frac{dz}{dn}(z_0, z) \right|^{-1}. \tag{S48}$$

For the density of segments within the gap, it is only determined by bound brushes that all started at the midpoint, namely $\epsilon(z_0) = \sigma f \delta(H - z_0)$ such that

$$\varphi(z > L) = \varphi_b(z) = \sigma f \frac{P}{H - L} \tag{S49}$$

and is constant within the gap. Since the potential is continuous between the gap and the layer, from $U(L)$ we obtain

$$A = \frac{\pi^2}{8N^2} L^2 + \frac{\omega \sigma f P}{H - L}. \tag{S50}$$

We can now use the fact that the total number of segments in the parabolic region has to sum up to the contributions of the bound and unbound brushes as

$$\int_0^L \varphi(z) dz = \sigma \left( (1 - f) N + f(N - P) \right) \tag{S51}$$

such that

$$\left[ \frac{\pi^2}{8N^2} L^2 + \frac{\omega \sigma f P}{H - L} \right] L - \frac{\pi^2}{8N^2} \frac{L^3}{3} = \omega \sigma \left( (1 - f) N + f(N - P) \right) \tag{S52}$$

Simplifying to

$$\frac{\pi^2}{12 N^3 \omega \sigma} L^3 = 1 - f \frac{P}{N} \left( \frac{H}{H - L} \right) \tag{S53}$$

and recognizing $h_{eq}^3 = \frac{12 N^3 \omega \sigma}{\pi^2}$ we finally obtain

$$\boxed{f(P) = \frac{N}{P} \frac{H - L}{H} \frac{h_{\text{eq}}^3 - L^3}{h_{\text{eq}}^3}} \tag{S54}$$

such that all parameters are now expressed with respect to a single parameter $P$ or $P/N$.

**Energy minimization** To minimize energy we still need to be able to compute the bound and unbound contributions to $\varphi(z)$ and $\epsilon(z_0)$ to be able to calculate the contributions to the energy.

Integrating formally the equation of motion we have in general that

$$\frac{dz}{dn} = \sqrt{2[-U(z) + U(z_0)] + \left( \frac{dz}{dn}(z = z_0) \right)^2} \tag{S55}$$

such that for the bound tethers we have for $z < L$

$$\frac{dz_b}{dn} = \sqrt{\frac{\pi^2}{4N^2} (L^2 - z^2) + \left( \frac{H - L}{P} \right)^2} \tag{S56}$$

and the contribution of the bound tethers to the segment density simply integrates as

$$\varphi_b(z) = \sigma f \left( \frac{\pi^2}{4N^2} \left( L^2 - z^2 \right) + \left( \frac{H - L}{P} \right)^2 \right)^{-1/2} \tag{S57}$$

From the integral expression giving $\varphi(z)$ we can invert the result to obtain the distribution of unbound ends

$$\epsilon_u(z_0) = \sigma\frac{3}{2h_{\text{eq}}^3}\left[2z_0\sqrt{L^2 - z_0^2} + \frac{2z_0\sqrt{L^2 - z_0^2}\sigma f\omega}{\frac{H-L}{P}\left(\frac{\pi^2}{4N^2}\left(L^2 - z_0^2\right) + \left(\frac{H-L}{P}\right)^2\right)}\right] \tag{S58}$$

and one may check that this expression verifies indeed

$$\int_0^L \epsilon_u(z_0)\,dz_0 = (1-f)\sigma \tag{S59}$$

where $f$ is expressed as above.

Now we can integrate the different contributions to the energy, namely

$$\varphi_{\text{excluded}} = k_BT\frac{\omega}{2\sigma}\int_0^H \varphi^2(z)\,dz = k_BT\frac{1}{2\sigma\omega}\left(\int_0^L (A - Bz^2)^2\,dz + \int_L^H \omega^2\varphi_b(z > L)^2\,dz\right) \tag{S60}$$

such that we find

$$\varphi_{\text{excluded}} = k_BT\frac{1}{2\sigma\omega}\left(A^2L - \frac{2}{3}ABL^3 + \frac{B^2L^5}{5} + \sigma^2\omega^2f^2\frac{P^2}{H-L}\right) \tag{S61}$$

where all parameters are known. We can rewrite this expression in a simpler form, and back in "real" units, as

$$\boxed{\frac{\varphi_{\text{excluded}}}{k_BT} = \frac{h_{\text{eq}}^2}{N\ell^2}\frac{\pi^2}{12}\left(\frac{h_{\text{eq}}}{H}\left(1 - \frac{L^3}{h_{\text{eq}}^3}\right)\frac{L^3}{h_{\text{eq}}^3} + \frac{3}{5}\frac{L^5}{h_{\text{eq}}^5} + \frac{1}{2}f^2\frac{P^2}{N^2}\frac{H}{h_{\text{eq}}}\frac{h_{\text{eq}}^2}{(H-L)^2}\right).} \tag{S62}$$

Importantly we notice that $\varphi_{\text{excluded}}$ may be entirely expressed with only $h_{\text{eq}}$, $\ell$ and $N$ representing brush parameters.

A slightly more cumbersome contribution to evaluate is the stretching part

$$\begin{aligned}\varphi_{\text{stretch}} =& \frac{1}{\sigma}\int_0^L \epsilon_u(z_0)\int_0^N \frac{1}{2}\left(\frac{dz_u}{dn}\right)^2 dn\,dz_0 \\ &+ \frac{1}{\sigma}\int_0^H \epsilon_b(z_0)\int_0^N \frac{1}{2}\left(\frac{dz_b}{dn}\right)^2 dn\,dz_0 \\ =& \varphi_{\text{stretch,u}} + \varphi_{\text{stretch,b}}.\end{aligned} \tag{S63}$$

Stretching due to the bound tethers is simple enough since $\epsilon_b(z_0) = f\sigma\delta(z_0 - H)$

$$\begin{aligned}\frac{\varphi_{\text{stretch,b}}}{k_BTf} &= \int_0^{N-P}\frac{L^2}{2\cos^2\left(\frac{\pi P}{2N}\right)}\left(\frac{\pi}{2N}\right)^2\cos^2\left(\frac{\pi n}{2N}\right)dn + \int_{N-P}^N \frac{1}{2}\left(\frac{H-L}{P}\right)^2 dn \\ &= \frac{L^2}{\cos^2\left(\frac{\pi P}{2N}\right)}B\left(\frac{N-P}{2} + \frac{N}{2\pi}\sin\frac{P\pi}{N}\right) + \frac{1}{2}\frac{(H-L)^2}{P}\end{aligned} \tag{S64}$$

and for the unbound tethers we have

$$\int_0^N \frac{1}{2}\left(\frac{dz_u}{dn}\right)^2 dn = \int_0^N \frac{z_0^2}{2}\left(\frac{\pi}{2N}\right)^2\cos^2\left(\frac{\pi n}{2N}\right)dn = \frac{Nz_0^2}{2}B \tag{S65}$$

and thus we can integrate simply and find

$$\varphi_{\text{stretch,u}} = k_BTB\frac{3NL^5}{4h_{\text{eq}}^3}\left[\frac{4}{15} + \frac{2fh_{\text{eq}}^3\left(4/\tan(\pi P/2N) + 3(1 + \tan(\pi P/2N)^2)\left(\pi(P/N - 1) + \sin(\pi P/2N)\right)\right)}{9L^3\pi}\right]. \tag{S66}$$

The stretch terms may also be simplified to write, with $p = \frac{\pi P}{2N}$, and back in "real" units

$$\boxed{\begin{aligned}\frac{\varphi_{\text{stretch}}}{k_BT} &= \frac{h_{\text{eq}}^2}{N\ell^2}\frac{L^2}{h_{\text{eq}}^2}\frac{\pi^2}{120}\left(3\frac{L^3}{h_{\text{eq}}^3} - \frac{5}{\pi}\frac{f}{\sin(2p)}\left[-5+\cos(2p)+3\pi\tan(p)\frac{N-P}{N}\right]\right)\\ &+ \frac{h_{\text{eq}}^2}{N\ell^2}\frac{f}{2}\left(\frac{\pi^2}{4}\frac{L^2}{h_{\text{eq}}^2}\frac{1}{\cos^2(p)}\left[\frac{N-P}{2N}+\frac{\sin(2p)}{2\pi}\right]+\left(\frac{H-L}{h_{\text{eq}}}\right)^2\frac{N}{P}\right).\end{aligned}} \tag{S67}$$

Again here we find that the stretching energy is only dependent on $h_{\text{eq}}$, $N$ and $\ell$ brush parameters.

The total energy of the system is the sum of all contributions

$$\varphi_{\text{unified}} = \varphi_{\text{steric}} + \varphi_{\text{bind}} = \varphi_{\text{stretch}} + \varphi_{\text{excluded}} + \varphi_{\text{bind}}. \tag{S68}$$

Now we can set the value of $P$ such that

$$\boxed{\frac{d\varphi_{\text{unified}}}{dP} = 0} \tag{S69}$$

allowing to fully close the system and find all unknowns. All in all, our derivation follows very closely that of Meng and Russel[53] that was performed for telechelic polymers, but for a careful adaptation of the binding energy term. We now turn to asymmetric coatings.

### 3.4.2 Asymmetric brushes

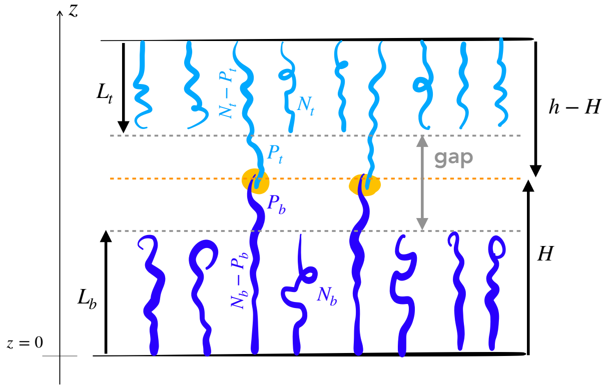


**Figure S11:** Combined theory setup to account both for steric repulsion and binding of polymer brush layers simultaneously, in an asymmetric setting where the top and bottom brush layers do not have the same properties.

For asymmetric brushes we employ a methodology exploited for non interacting asymmetric polymers and shown to yield satisfying results experimentally[54] and that was proven to be exact in the classical limit[55]. The interface between the brushes is now at an unknown height $H$ (see Fig. S11). One brush therefore extends by $H$ while the other one by $h - H$ such that $h = H + (h - H)$ is the spacing between the plates. As the brushes are asymmetric, we do not necessarily have $H = h/2$. Our procedure is to evaluate the total energy of the system and minimize that energy with respect to $H$. In contrast with Ref. 54,55 that do not investigate binding forces, here the total energy of the system is written as

$$\varphi_{\text{unified}} = \frac{\varphi_{\text{steric}}^{\text{bottom}}(2H) + \varphi_{\text{steric}}^{\text{top}}(2(h-H))}{2} + \varphi_{\text{bind}}. \tag{S70}$$

$\varphi_{\text{bind}}$ designates here the binding energy of the asymmetric system Eq. (S27). We now have 7 unknown parameters: the number of units in the gap on each side $P_t$ and $P_b$, the length of the unbound brush on each side, $L_t$ and $L_b$, the fraction of bound brushes on each side $f_t$ and $f_b$ and the height $H$. As above, $L_t$, $L_b$, $f_t$ and $f_b$ may all be expressed with respect to $P_t$ and $P_b$. Additionally, we have to require that the number of bound polymers on the top matches that of the bottom $f_t\sigma_t^{\text{sticky}} = f_b\sigma_b^{\text{sticky}}$ which reduces the unknown parameters to 2, say $P_t$ and $H$. We first minimize the energy at $H$ fixed with respect to $P_t$ and then iterate the process to minimize energy with respect to $H$.

### 3.4.3 Numerical implementation

**Code implementation** The computation of the free energy of interaction is implemented in a custom made Python routine. Numerical minimization of energy with respect to $P$ following Eq. (S69) is performed using a standard Brent quadratic algorithm (*scipy.optimize.brentq*). Minimization of $\varphi_{\text{unified}}$ with respect to $H$ in the asymmetric case with the same method led to inaccurate results. Instead a custom made minimization algorithm (spanning the entire interval of possibilities for $H$ and refining the search iteratively around the minimum) was shown to yield more accurate results. The accuracy of the minimum position $H$ was found to be satisfactory after 4 iterations with 10 discretization steps in space.

**Numerical tests** The asymmetric algorithm was tested on symmetric configurations and found to yield the same results (that $H = h/2$, and same energy values). The symmetric configuration was tested to reproduce the results of Ref. 53 in the case where $\Delta G^{ab} = 0$. Finally, at low binding fractions, the binding fraction $f$ is equal to the self consistent result of Ref. 43.

## 3.5 van der Waals interactions

To describe van der Waals interactions, we use the linear superposition formula that gives the van der Waals potential interaction between a sphere of radius $a$ and a surface at distance $h$ of the sphere[56–58]

$$\phi_{\text{vdW}}(h) = -\frac{A(h)}{6}\left\{\frac{2a}{h}\frac{h+a}{h+2a} - \ln\left(\frac{h+2a}{h}\right)\right\} \tag{S71}$$

where $A(h)$ is the retarded and screened Hamaker constant. This formula was successfully used by a number of authors to account for van der Waals interactions between colloids and surfaces[59,60].

### 3.5.1 Hamaker constant

We calculate the screened and retarded Hamaker constant using the formalism detailed both in Ref. 58 and based on Lifschitz theory Ref. 61 and 61 (Note that in Ref. 59 it seems that a $2\pi$ factor is missing from their definition of $\xi_n$, that is not missing in any of the other references we explored.). In our experiments we use two different configurations, one of glass slides and polystyrene particles immersed in water; and one where the glass slides are coated by a polystyrene layer . We ignore the screening and charge effects due to the possible brushes on each surface. We therefore have one Hamaker constant to calculate (for the Glass - Water - Polystyrene system):

$$A_{GWPs}(h) = \frac{k_B T}{8\pi h^2}\sum_{n=0}^{\infty,\prime}\int_{r_n}^{\infty} x \ln\left[(1-\overline{\Delta}_{GW}\overline{\Delta}_{PW}e^{-x})(1-\Delta_{GW}\Delta_{PW}e^{-x})\right]dx \tag{S72}$$

where

$$\overline{\Delta}_{km} = \frac{x_m\epsilon_k - x_k\epsilon_m}{x_m\epsilon_k + x_k\epsilon_m} \text{ and } \Delta_{km} = \frac{x_m - x_k}{x_m + x_k} \tag{S73}$$

(Note that in this step we ignored the differences between the magnetic susceptibilities in the different media, since our material are all non metallic.). And furthermore we have

$$x_k(x)^2 = x^2 + \left(\frac{2h\xi_n}{c}\right)^2(\epsilon_k - \epsilon_W) \text{ with } \xi_n = n\frac{2\pi k_B T}{\hbar} \tag{S74}$$

and finally on the bounds for summation factors

$$r_n = \frac{2h\sqrt{\epsilon_W}}{c}\xi_n \tag{S75}$$

and the $\sum'$ in Eq. (S72) indicates that the first term ($n = 0$) was multiplied by $\frac{1}{2}\left(1 + 2\frac{h}{\lambda_D}\right)e^{-2h/\lambda_D}$ to account for screening of the zero mode frequency by the salt solution (and the factor 1/2 in front avoids

a double counting)[58,59]. In all of these calculations, $\epsilon_k = \epsilon_k(i\xi_n)$ is the relative dielectric relative permittivity evaluated at the imaginary frequency $i\xi_n$. In full generality, the relative dielectric permittivity can be modeled by

$$\epsilon(i\xi_n) = 1 + \sum_j \frac{d_j}{1 + \xi_n \tau_j} + \sum_j \frac{f_j}{\omega_j^2 + g_j \xi_n + \xi_n^2} \tag{S76}$$

where the parameters $d_j, \tau_j, f_j, g_j, \omega_j$ all correspond to either Debye dipolar relaxation parameters or damped-oscillator parameters of wave modes of the material considered. For consistency we recapitulate here the parameters used in our numerical model. The data is based on data given in[58] unless mentioned otherwise. Note that to convert from the $eV$ units to rad.s$^{-1}$, the conversion factor $e/\hbar$ was used where $e$ is the elementary charge.

For glass we use the data measured in Ref. 59 which allows one to write

$$\epsilon_G(i\xi_n) = n_G^2(i\xi_n) = 1 + \frac{C_{UV}}{1 + \xi_n^2/\omega_{UV}^2} \tag{S77}$$

where $n_G$ is the refractive index of glass and $C_{UV} = 1.282$ and $\omega_{UV} = 1.911 \times 10^{16}$ rad.s$^{-1}$ are the fitted parameters taken from Ref. 59. We have checked that using slightly different parameters for glass modeling does not significantly alter the results.

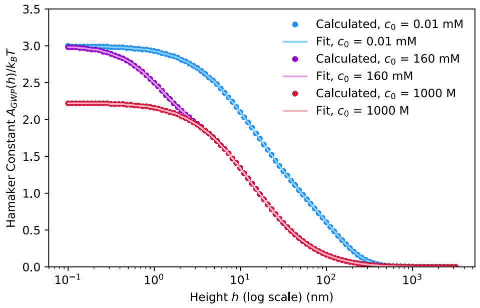


**Figure S12:** Hamaker constant for the glass-water-PS system, for different salt concentrations $c_0$, comparing the fully calculated data with the fitted data, at 20°C

### 3.5.2 Computing the Hamaker constant

To increase computational speeds, Hamaker constants are pretabulated in the range of relevant distances, temperatures, and salt concentrations (100 values of $h$ going from 1 Å to 300 nm, 20 values of temperature from 20 to 100°C, and 20 values of (monovalent) salt concentration from $10^{-5}$ to $10^3$ M.). These pre-tabulated values are fitted using a basic grid fitting algorithm (Python's, RegularGridInterpolator) which was tested against other methods (like standard artificial intelligence routines[62]) and yielded the best results. The fit is done once and the model fit is subsequently efficiently used to evaluate the Hamaker constant at parameters of interest.

We show in Fig. S12 the Hamaker constant. Our results agree with the original results by Bevan and Prieve[59]. In the concentration regimes that we are interested in ($c_0 = 100 - 200$ mM) and in the range of temperatures relevant to us, we expect $A(h \sim 50\text{ nm}) \sim 0.1 k_BT$. Note that this may seem small, but in fact Eq. (S71) shows that $\phi_{vdw} \sim A(h)R/3h$. With $R = 2.5\,\mu$m as in most our samples and $h \sim 50$ nm then $\phi_{vdw} \sim k_BT$ and therefore we have to expect van der Waals forces to be relevant.

## 3.6 Surface charge electrostatic interactions

**Relative dielectric permitivity of water** The relative dielectric permittivity of water at different temperature and different densities is accounted for by the fit model of Ref. 63. In every instance where it is required it is calculated using this fit.

**In the absence of coating on either plate or colloid** We first write the electrostatic interaction problem in the absence of coating, within the Derjaguin approximation. The surfaces are both negatively charged resulting in a repulsion force. The repulsive potential between the two charged plates within the

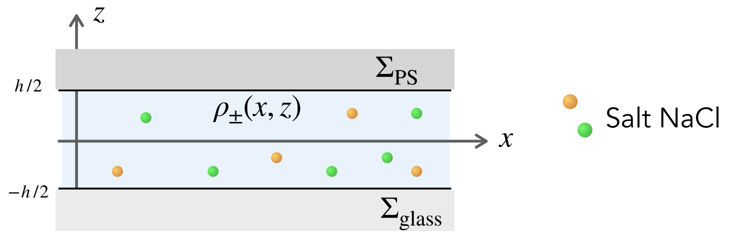


**Figure S13:** Sketch of the electrostatic potential (close up) with no coatings.

Gouy-Chapman approximation simply writes[64–66]

$$\frac{\varphi_{\mathrm{DL}}}{k_B T} = 64\lambda_D c_0 \gamma_{\mathrm{PS}} \gamma_{\mathrm{glass}} e^{-h/\lambda_D}. \tag{S78}$$

where ”DL” stands for double layer,

$$\lambda_D^2 = \frac{\epsilon_r \epsilon_0 k_B T}{2e^2 c_0} \tag{S79}$$

is the Debye length with $c_0$ the salt concentration in the bulk, and the parameters $\gamma_i$ are given by the Grahame equation

$$\gamma_i = \tanh\left(\frac{1}{2}\sinh^{-1}\left(\frac{\Sigma_i \lambda_D e}{2\epsilon_0 \epsilon_r k_B T}\right)\right) \tag{S80}$$

Using Derjaguin's approximation we can obtain the full electrostatic repulsion potential for the sphere

$$\frac{\phi_{DL}(h)}{k_B T} = 128\pi R \lambda_D^2 c_0 \gamma_{\mathrm{PS}} \gamma_{\mathrm{glass}} e^{-h/\lambda_D} \tag{S81}$$

**Inferring surface charge from calibration experiments** Let's rewrite the full electrostatic and gravity potential for a sphere of radius a, made of polystyrene and facing a glass wall at distance $h$, we get

$$\frac{\phi(h)}{k_B T} = 64\pi a \frac{\epsilon_0 \epsilon_r k_B T}{e^2} \gamma_{\mathrm{glass}} \gamma_{\mathrm{PS}} e^{-h/\lambda_D} + \frac{4}{3}\pi a^3 \left(\rho_{PS} - \rho_W(T)\right) \frac{gh}{k_B T} \tag{S82}$$

The surface charge of polystyrene is given by the manufacturer as $\Sigma_{\mathrm{PS}} = 19\ \mathrm{mC/m^2}$ and therefore the only unknown parameter is $\Sigma_{\mathrm{Glass}}$. Calibration TIRM experiments on bare colloids indicate that for salt concentrations above $c_0 = 2mM$ particles crash on the glass surface due to van der Waals attraction – see Fig. S14. This allows to infer a charge density for glass of about $\Sigma_{\mathrm{Glass}} = 0.5\ \mathrm{mC/m^2}$ – for which the potential depth significantly drops around and above $c_0 = 2mM$. Below that value, in simulations the electrostatic repulsion is too weak and already at $c_0 = 1mM$ a significant potential drop is seen; while above no significant drop is seen. The uncertainty region is about $\Sigma_{\mathrm{Glass}} = 0.5 \pm 0.25\ \mathrm{mC/m^2}$ and we have checked that our further results do not depend on this uncertainty range. This value is consistent with other reported measurements[67].

With all parameters set we compare simulation results to experimental results on bare particles and find excellent agreement – see Fig. S15. Note that a slight better agreement between simulation and experiments is found when using a radius value 3.1 $\mu$m slightly above the mean radius of the PS particles (of 3 $\mu$m). 3.1 $\mu$m is within the range of polydispersity as provided by the manufacturer. Note that for these systems noise on experimental measurements is not relevant since the potential curves are broader than the limiting value.

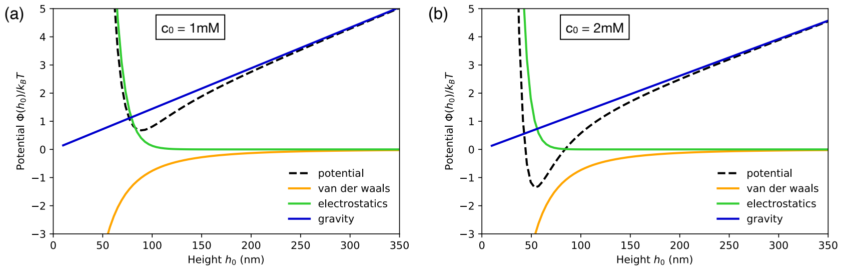


**Figure S14:** Bare colloid potential simulations for a bare particle of radius 3 $\mu$m at room temperature $T = 22°C$ in a salt concentration (a) $c_0 = 1mM$ and (b) $c_0 = 2mM$ for a glass surface charge $\Sigma_{\text{Glass}} = 0.5$ mC/m$^2$. Simulations use the potential profile described in Eq. (S82) (gravity and electrostatics) but include also van der Waals forces via Eq. (S71).

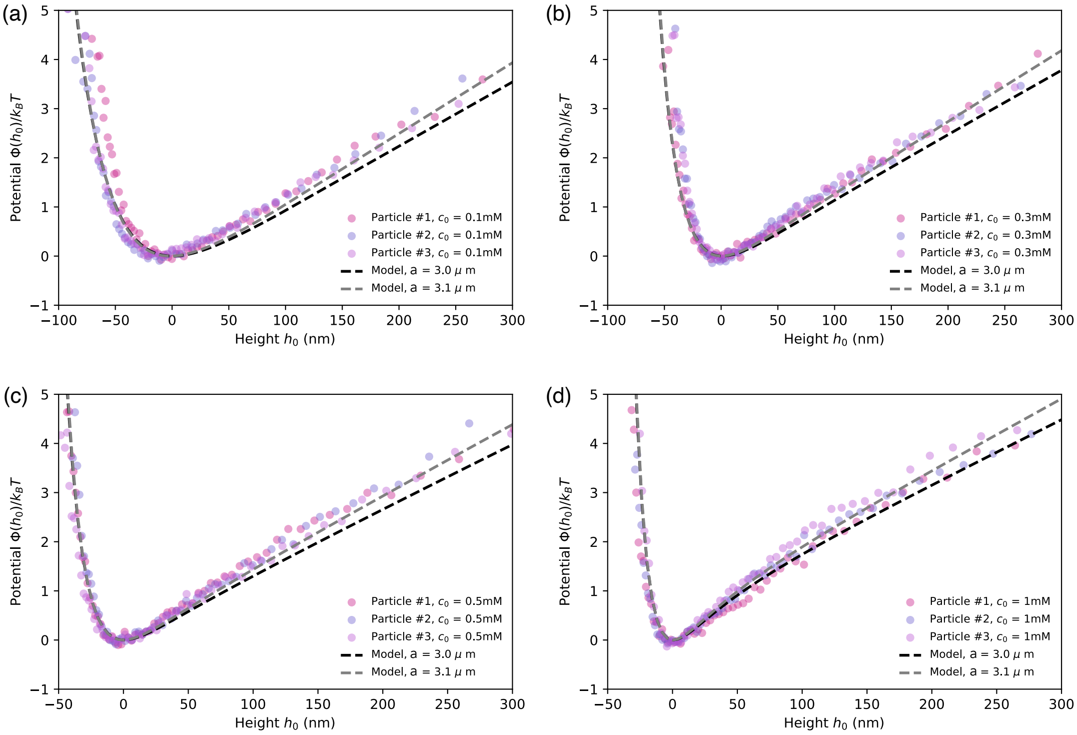


**Figure S15:** Bare colloid potential measurements and simulations for bare particles of radius 3 $\mu$m at room temperature $T = 22°C$ in a salt concentration (a) $c_0 = 0.1mM$, (b) $c_0 = 0.3mM$, (c) $c_0 = 0.5mM$, (d) $c_0 = 1mM$ using a glass surface charge $\Sigma_{\text{Glass}} = 0.5$ mC/m$^2$. Simulations use the potential profile described in Eq. (S82) (gravity and electrostatics) but include also van der Waals forces via Eq. (S71).

## 3.7 Inferring microscopic parameters

**Number of bound DNA pairs.** Energy minimization (of steric repulsion and binding forces) allows to directly the fraction of bound pairs $f(T, h)$ at a given plate-plate separation $h$ and temperature $T$. Using Dejarguin's approximation allows to infer the number of bound pairs over the whole particle at a given distance $h$ from the surface

$$N_{\text{bound}}(h) = 2\pi a \sigma_{\text{sticky}} \int_h^{\infty} f(T, h')dh' \tag{S83}$$

where $a$ is the particle radius (we neglect dilatation effects here). The average number of bound pairs at a given temperature is then given by the statistical average

$$\langle N_{\text{bound}} \rangle(T) = \frac{\int_0^{\infty} N_{\text{bound}}(h) e^{-\beta\phi(h,T)}dh}{\int_0^{\infty} e^{-\beta\phi(h,T)}dh} \tag{S84}$$

where $\phi(h,T)$ is of course the full free energy of interaction of the particle with the surface. For asymmetric particles, similarly $\sigma_{\text{sticky}}$ corresponds with the maximal number of sticky bonds that may be formed between top and bottom (thus corresponding to the smallest (sticky) coating density between top and bottom).

**Radius of interaction and number of DNA pairs within reach.** To count the number of pairs within reach, we search for the typical radius of interaction of the particle with the surface. Let $h$ be the (minimum) height of the particle to the surface. We define $h_{\text{max}}(h)$ the maximal distance at which the particle is still significantly interacting with the surface. We take it such that at that distance to the surface, the probability that the bonds are attached is less than 1%, $f(h_{\text{max}}) = 1\%$. The radius of interaction is therefore, using a simple geometrical argument

$$R_{\text{int}} = \sqrt{a^2 - (a - h_{\text{max}} + h)^2}. \tag{S85}$$

In turn the number of DNA pairs within reach is the number of strands in that circle. We define it as

$$N_{\text{contact}}(h) = \pi R_{\text{int}}^2 \min(\sigma_t^{\text{sticky}}, \sigma_b^{\text{sticky}}). \tag{S86}$$

This corresponds to the number of pairs within reach at height $h$. The average value of potential pairs in contact is therefore the statistical average

$$\langle N_{\text{contact}}\rangle(T) = \frac{\int_0^\infty N_{\text{contact}}(h) e^{-\beta\phi(h,T)} dh}{\int_0^\infty e^{-\beta\phi(h,T)} dh}. \tag{S87}$$

$\langle N_{\text{contact}}\rangle(T)$ (available pairs) and $\langle N_{\text{bound}}\rangle(T)$ (bound pairs) are plotted in Fig. 4e (respectively in blue and pink) of the main paper.

**Bound pairs for the diversity of systems explored in the main paper** We report in Table S11 the number of bound pairs at typical temperatures for the variety of systems investigated in this work.

| **System** | **Bound pairs at melting** $\langle N_{\text{bound}}\rangle(T_m)$ | **Available pairs at melting** $\langle N_{\text{contact}}(T_m)\rangle$ |
|---|---|---|
| *Systems explored in Fig. 3a of main paper* | | |
| $A^6/B^6$, 34 k PEO, 100% coverage | 7-8 | 250 – 380 |
| $A^5/B^5$, 34 k PEO, 100% coverage | 3-7 | 190 – 300 |
| $A^5/B^5$, 11 k PEO, 100% coverage | 4-7 | 120 – 220 |
| $A^5/B^5$, 6.5 k PEO, 100% coverage | 5-9 | 130 – 220 |
| $A^4/B^4$, 34 k PEO, 100% coverage | 3-7 | 130 – 250 |
| $A^4/B^4$, 11 k PEO, 100% coverage | 5-7 | 150 – 230 |
| $A^4/B^4$, 6.5 k PEO, 100% coverage | 3-4 | 110 – 180 |
| *Systems explored in Fig. 4b of main paper* | | |
| $A^6/B^6$, 34 k PEO, 100% coverage | 4-8 | 400 |
| $A^6/B^6$, 34 k PEO, 50% coverage | 3-7 | 250 – 280 |
| $A^6/B^6$, 34 k PEO, 20% coverage | 2-5 | 150 – 190 |

**Table S11:** Number of bound pairs at the respective melting temperature of the system investigated and at room temperature for the variety of systems investigated in this work. Error bars correspond to the typical variation of $\sim 0.2$°C around the melting temperature.

# 4 Accounting for shot noise on model curves

In a separate paper[68], we have shown that shot noise is the dominant source of noise for experimental TIRM measurements of potential curves.

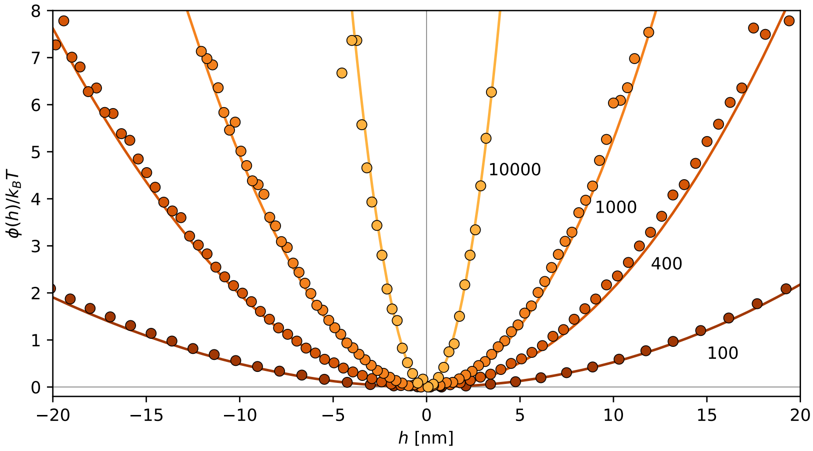


**Figure S16:** TIRM measurements on an immobilized particle for different target count values $N_{\text{photons}}$. Solid lines show expected broadening for each value of $N_{\text{photons}}$ using Eq. (S105). Note that the data and model are not symmetric about the $y$-axis, especially for the smaller values of $N_{\text{photons}}$.

## 4.1 Shot noise on model curves

In a TIRM experiment, the intensity of light scattered by a particle at a height $h_0$ above the substrate is given by

$$I(h_0) = I_0 e^{-\alpha h_0} \tag{S88}$$

where $\alpha$ is the inverse penetration depth of the evanescent light, which depends on the angle of incidence with the substrate. For our experiments, $\alpha^{-1} = 99$ nm. For a given intensity $I$ of light incident on a photon counting detector, the probability of observing $N$ photons in a time interval $\Delta t$ is governed by a Poisson distribution[2]

$$p_n(N, \bar{N}) = \frac{(\bar{N})^N}{N!} e^{-\bar{N}} , \tag{S89}$$

where $\bar{N} = I\Delta t$ is the mean number of photons detected in a time $\Delta t$, and $I$ has units of counts per unit time.

To begin, let's suppose that the height $h_0$ of the particle is fixed. Even though the particle's position is fixed, the number of photons $N$ observed in a time interval $\Delta t$ will be distributed around the mean value $\bar{N}$ according to Eq. (S89). Thus, our first task is to determine the distribution $p(h|h_0)$ of apparent heights $h$ resulting from this distribution. Once this is done, our second task is to determine how this broadened distribution of apparent heights affects TIRM measurement of the potential. Because the particle moves rather slowly, we simply assume $p(h) = \int p(h|h_0)\, p(h_0)\, dh_0$. Because the height probabilities are Boltzmann laws,

$$\boxed{\frac{\phi_{\text{distorted}}(h)}{k_BT} = -\ln\left(\int dh_0\, e^{-\beta\phi_{\text{real}}(h_0)} p(h|h_0)\right)} . \tag{S90}$$

[2]Note that the Poisson distribution counting the number of photons has no connection with the so-called "Poisson approximation" to model binding interactions.

**Set integration time.** We first consider the case where the time interval for photon detection is fixed to some value $\tau_0$, so $\Delta t = \tau_0$. We now want to find an expression for $p(h|h_0)$. We can rewrite the distribution of detected photons given by Eq. (S89) as

$$p_n(N, I_0 e^{-\alpha h_0}\tau_0) = \frac{(I_0 e^{-\alpha h_0}\tau_0)^N}{N!} e^{-I_0 e^{-\alpha h_0}\tau_0}. \tag{S91}$$

We relate the distribution of photons to the distribution of heights using

$$p(h|h_0)|dh| = p_n(N, I_0 e^{-\alpha h_0}\tau_0)|dN| \;. \tag{S92}$$

Since we have set $\tau_0$ as the time interval for counts, each time we detect $N$ photons we (naively) relate the measurement of $N$ to a height $h$ such that

$$N = \tau_0 I_0 e^{-\alpha h} \equiv N_0 e^{-\alpha h} \;. \tag{S93}$$

Solving for $p(h|h_0)$ gives

$$p(h|h_0) = \left|\frac{dN}{dh}\right| p_n(N, I_0 e^{-\alpha h_0}\tau_0) = \alpha \frac{(N_0 e^{-\alpha h_0})^{N_0 e^{-\alpha h}}}{(N_0 e^{-\alpha h})!} e^{-N_0 e^{-\alpha h_0}\tau_0} N_0 e^{-\alpha h}. \tag{S94}$$

Putting this all together, we obtain

$$\boxed{\frac{\phi_{\text{distorted}}(h)}{k_B T} = -\ln\left(\int dh_0\, e^{-\beta\phi_{\text{real}}(h_0)} \alpha N_0 e^{-\alpha h} p_n(N_0 e^{-\alpha h}, N_0 e^{-\alpha h_0})\right)}\;, \tag{S95}$$

where we recall that $p_n$ is the Poisson distribution. The only parameter in this expression $N_0 = I_0\tau_0$ can be determined directly from the TIRM measurements. For example, at the minimum in the potential the average number of photons detected per integration time is accurately measured and given by $N_{\text{photons}} = N_0 e^{-\alpha h_{\text{min}}}$. Using this relation, the expression for the shot noise distorted potential becomes

$$\boxed{\frac{\phi_{\text{distorted}}(h)}{k_B T} = -\ln\left(\int dh_0\, e^{-\beta\phi_{\text{real}}(h_0)} \alpha N_{\text{photons}} e^{-\alpha(h-h_{\text{min}})} p_n(N_{\text{photons}} e^{-\alpha(h-h_{\text{min}})}, N_{\text{photons}} e^{-\alpha(h_0-h_{\text{min}})})\right)}. \tag{S96}$$

In a typical measurement, $N_{\text{photons}} \approx 1000$.

**Set target number of photons.** Alternatively, as was done in our experiments, the number of photons can be set to a target value; here $N_{\text{photons}} = 1000$. Therefore, the time interval for each measurement will have some distribution say $p_t(\Delta t, N = N_{\text{photons}})$, due to the fact that to acquire the target number of photons $N_{\text{photons}}$ takes a random amount of time. For each measurement we thus get a random $\Delta t$ that corresponds to some intensity $I = N_{\text{photons}}/\Delta t$, that we can connect to a height as $I = N_{\text{photons}}/\Delta t = I_0 e^{-\alpha h}$.

We thus need to express $p_t(\Delta t, N)$, which is the conditional probability that the time interval has some value $\Delta t$, given that the number of photons $N$ is fixed to be $N_{\text{photons}}$. According to Bayes theorem,

$$p_t(\Delta t, N)\, \delta(N - N_{\text{photons}}) = p_n(N, I_0 e^{-\alpha h_0}\Delta t)\, \delta(\Delta t - \tau_0)\;, \tag{S97}$$

such that $\tau_0 I_0 e^{-\alpha h_0} = N_{\text{photons}}$. In general, the number of counted photons and the integration time are related as $N = \Delta t\, I_0 e^{-\alpha h}$. As the delta functions are in essence distributions, we can relate them as follows,

$$\delta(N - N_{\text{photons}})\, |dN| = \delta(\Delta t - \tau_0)\, |d\Delta t|\;, \tag{S98}$$

so that

$$\delta(N - N_{\text{photons}})\, I_0 e^{-\alpha h} = \delta(\Delta t - \tau_0)\;, \tag{S99}$$

and finally,

$$p_t(\Delta t, N)\,\delta(N - N_{\text{photons}}) = p_n(N, I_0 e^{-\alpha h_0}\Delta t)\,\delta(N - N_{\text{photons}})\,I_0 e^{-\alpha h}\;. \tag{S100}$$

Replacing in the above expressions $N$ by $N_{\text{photons}}$ we obtain the distribution of integration times

$$p_t(\Delta t, N_{\text{photons}}) = p_n(N_{\text{photons}}, I_0 e^{-\alpha h_0}\Delta t)\,I_0 e^{-\alpha h}. \tag{S101}$$

Finally, we relate the distribution of measured heights $h$ to the distribution of integration times as

$$p(h|h_0)\,|dh| = p_t(\Delta t)\,|d\Delta t|\;. \tag{S102}$$

Since $N_{\text{photons}} = \Delta t\, I_0 e^{-\alpha h}$, we have $\Delta t = N_{\text{photons}}\, e^{\alpha h}/I_0$ and hence

$$p(h|h_0) = \alpha p_t(\Delta t)\frac{N_{\text{photons}}}{I_0}e^{\alpha h}. \tag{S103}$$

Gathering all things yields

$$p(h|h_0) = \alpha p_n(N_{\text{photons}}, N_{\text{photons}} e^{-\alpha(h_0-h)})\,N_{\text{photons}}. \tag{S104}$$

Notice that $\Delta t$ and $I_0$ disappear in this expression.

From Eq. (S90), we now obtain

$$\boxed{\frac{\phi_{\text{distorted}}(h)}{k_B T} = -\ln\left(\alpha N_{\text{photons}}\int dh_0\, e^{-\beta\phi_{\text{real}}(h_0)} p_n(N_{\text{photons}}, N_{\text{photons}} e^{-\alpha(h_0-h)})\right)} \tag{S105}$$

To interpret this expression in terms of sum running over a "number of photon counts", we can make the change of variables $n = N_{\text{photons}} e^{-\alpha(h_0-h)}$, which gives

$$\boxed{\frac{\phi_{\text{distorted}}(h)}{k_B T} \simeq -\ln\left(\sum_{n=1}^{\infty} e^{-\beta\phi_{\text{real}}\left(h-\frac{1}{\alpha}\ln\left(\frac{n}{N_{\text{photons}}}\right)\right)} p_n(N_{\text{photons}}, n)\frac{N_{\text{photons}}}{n}\right)} \tag{S106}$$

In Fig. S16, we show TIRM data obtained for an immobilized 5-$\mu$m particle for different values of $N_{\text{photons}}$. As expected, the measured width of potential becomes narrower as $N_{\text{photons}}$ increases from 400 to 10,000. The data are well described by the shot-noise kernel given by Eq. (S105) for all values of $N_{\text{photons}}$.

**Other sources of noise.** There are other sources of noise, including the PMT dark count and stray light. These can be subtracted off the raw TIRM signal, which we routinely do, but doing so makes a negligible difference in the potentials extracted. Laser intensity fluctuations, which are less than 1%, play no noticeable role as the shot noise measurements for an immobilized particle are well described by Eq. (S105). With the exception of shot noise, other sources of noise similarly have negligible effect on our measurements of the potential.

## 4.2 Shot noise does not affect melting curves

Numerically we find little difference between melting curves obtained through Eq. (S13) using either model potentials or shot noise distorted model potentials. We explain this observation below.

Eq. (S13) shows that to calculate the melting curve one needs to calculate typical integrals of the Boltzmann factor (here for the distorted potential)

$$\begin{aligned}\int_0^{h_{\text{range}}} e^{-\beta\phi_{\text{distorted}}(h)}dh &\simeq \int_0^{h_{\text{range}}} \sum_{n=1}^{\infty} p_n(N_{\text{photons}}, n)\,\frac{N_{\text{photons}}}{n} e^{-\beta\phi_{\text{real}}\left(h-\frac{1}{\alpha}\ln\left(\frac{n}{N_{\text{photons}}}\right)\right)}dh \\ &= \sum_{n=1}^{\infty} p_n(N_{\text{photons}}, n)\,\frac{N_{\text{photons}}}{n}\int_{h_n}^{h_{\text{range}}+h_n} e^{-\beta\phi_{\text{real}}(h)}dh\end{aligned} \tag{S107}$$

where we wrote $h_n = -\frac{1}{\alpha}\ln\left(\frac{n}{N_{\text{photons}}}\right)$. Typically, considering the shape of the potential we know that the value of the integral does not change much so long as $h_n$ is not too large, say $h_n \lesssim h_{\text{range}}/2$. This corresponds to values of $n$ ranging in $0.9N_{\text{photons}} \leq N \leq 1.1N_{\text{photons}}$. Typically, with $N_{\text{photons}} = 1000$ as is the case in our experiments, the Poisson distribution $p_n(N_{\text{photons}}, n)$ is still centered around $N_{\text{photons}}$ with standard deviation $\sigma_n \sim 30$. The interval where significant values of $p_n(N_{\text{photons}}, n)$ occur ranges typically over $3\sigma_n$, which is within the range where the integral doesn't change much. Therefore,

$$\int_0^{h_{\text{range}}} e^{-\beta\phi_{\text{distorted}}(h)}dh \simeq \sum_{n=N_{\text{photons}}-3\sigma_n}^{N_{\text{photons}}+3\sigma_n} p_n(N_{\text{photons}}, n)\frac{N_{\text{photons}}}{n}\int_0^{h_{\text{range}}} e^{-\beta\phi_{\text{real}}(h)}dh \simeq 1 \times \int_0^{h_{\text{range}}} e^{-\beta\phi_{\text{real}}(h)}dh \tag{S108}$$

since $n \simeq N_{\text{photons}}$ over this range of values of $n$ and $\sum_{n=N_{\text{photons}}-3\sigma_n}^{n=N_{\text{photons}}+3\sigma_n} p_n(N_{\text{photons}}, n) \simeq 1$. Thus the shot noise distorted potential yields a melting curve that is approximately independent of shot noise.

# 5 Model parameters and uncertainty evaluation

## 5.1 Recapitulation of experimental parameters used in the model

We recapitulate in the table below all the experimental parameters used in the model, their method of acquisition or calibration and how they are used in the model.

## 5.2 Sensitivity of the model on physical parameters

### 5.2.1 Uncertainties on the bare free energy calculations

Several authors have raised the question of how uncertainties on the thermodynamic quantities $\Delta H^0$ and $\Delta S^0$ as computed from the nearest neighbor (NN) model of SantaLucia[50], may affect the accuracy of melting point predictions[1,69]. In a follow-up paper, SantaLucia and Turner[70] described how uncertainties on measured values of $\Delta H^0$ and $\Delta S^0$, that were used to calibrate the NN model, could result in uncertainties on the prediction of the melting temperature $T_m^{ab}$ of individual sequences. They report, however, that measurement uncertainties typically compensate (between $\Delta H^0$ and $\Delta S^0$), yielding an uncertainty on $T_m^{ab}$ only of about 1°C. Additionally they provide a typical uncertainty range of $\sim 0.02$ kcal/mol per (sticky) nucleotide. In our case, this results in approximately a 1°C uncertainty on the melting temperature $T_m^{ab}$ of our bare sequences. Note that this uncertainty can be much larger, up to $\sim 0.2$ kcal/mol per (sticky) nucleotide on the free energy, depending on the context and of course depending on the temperature of the investigation (see *e.g.* Table 6 in Ref. 71).

We probe the effect of uncertainty on the bare free energy by including a $-0.02$ kcal/mol error per sticky nucleotide on $\Delta H^0$ for our $A^4/B^4$ sequence and report the obtained well depths in Fig. S17 (dashed lines), for the variety of PEO molecular weights used. In Fig. S17 we compare these to the reported experimental and theoretical predictions (full lines) reported in Fig. 3 of the main paper. We find a systematic decrease of 1°C of the curves. In fact although the model is non-linear in $\Delta G^0$ (as we will see in the next section), such a change in hybridization energy is small enough that it results in a linear effect: all curves are shifted along the temperature axis by the same amount, of about 1°C. The typical range obtained is clearly within experimental error.

### 5.2.2 Uncertainties on the grafting density of the glass slide

In Fig. S18 we assess the impact of a 10% error on the calibration of the grafting density of the glass slide. We calculate the interaction potential, with shot noise, for the lowest ($\sigma_g$ = (9.9 nm)$^{-1}$) and highest ($\sigma_g$ = (8.9 nm)$^{-1}$) density corresponding to a 10% error on the calibrated value $\sigma_g$ = (9.4 nm)$^{-1}$. We find in Fig. S18 that such an error corresponds to about a 1 °$C$ uncertainty on the melting temperature, which is well within the experimental uncertainty.

| Parameter | Notation and Value | Calibration method |
|---|---|---|
| *Measurement cell* | | |
| Temperature | $T = 22 - 70\,^{\circ}C$ | temperature control, known |
| Buffer salt concentration | $c_0 = 140$ mM | known |
| Solution density | $\rho_W(T) \simeq 1$ g/cm$^3$ + salt + thermal expansion | measured (see Sec. 3.1.1) |
| Water permittivity | $\epsilon_r(T)$ | from Ref. 63 |
| Height of the fluidic cell | $h_{\text{slab}} = 250\,\mu$m | known |
| Gravity field in New York City | $g = 9.802\,\text{m/s}^2$ | tabulated (Wolfram Alpha) |
| *DNA-coated colloid* | | |
| Colloid radius | $0.5 - 3\,\mu$m $\pm$ 10% | manufacturer |
| Colloid mass density | $\rho_{\text{PS}}(T) = 1.055$ g/cm$^3$ + thermal expansion | manufacturer + Ref. 33 for thermal expansion coefficient (see Sec. 3.1.1) |
| Brush density | $\sigma_p = (6.9\text{ nm})^{-2} - (2.7\text{ nm})^{-2}$ | measured separately (see Supplementary §2.2) |
| PEO molecular weight | $M_w = 6.5 - 34$ kg/mol | manufacturer |
| PEO persistence length | $\ell_{\text{PEO}} = 0.368$ nm | from molecular configuration, as $\ell_{\text{PEO}} = (2\ell_{C-C} + \ell_{C-O})\cos(68/2)$ [21] – in agreement with 0.7 nm in Ref. 20 |
| PEO brush length | 15 – 35 nm | measured separately (see Sec. 2.3) |
| DNA sticky sequence | refer to Table 1 of the main manuscript | known |
| DNA bare hybridization energy | $\Delta G^0_{ab}$ | see Table S10, from nearest neighbor model of Ref. 50. Effect of uncertainty explored in Sec. 5.2.1 |
| DNA persistence length | $\ell_{\text{DNA}} = 1.49$ nm | at 140 mM salt concentration [29]. Total resulting brush length is carefully calibrated in Sec. 2.3. |
| *DNA-coated glass surface* | | |
| Brush density | $\sigma_g = (9.4\text{ nm}^2)^{-1}$ | adjusted at 1 temperature value for 1 system design and kept constant (see Fig. 2-a) of the main paper. Effect of uncertainty explored in Sec. 5.2.2 |
| DNA sticky sequence | refer to Table 1 of the main manuscript | known |
| Surface charge density of glass | $\Sigma_{Glass} = 0.5$ mC/m$^2$ | calibrated (see Sec. 3.6) |
| *Apparatus* | | |
| Shot noise kernel | $\phi_{\text{distorted}}(h) = f_{\alpha,N_{\text{photons}}}(\phi_{\text{real}}(h))$ | see Eq. (S105) |
| Target photon count | $N_{\text{photons}} = 1000$ | known, as set by the experiment |
| Shot noise depth | $\alpha^{-1} = 99$ nm | calibrated, depends on the incidence angle |

**Table S12:** System parameters used in modeling calculations and method of acquisition

### 5.2.3 Influence of different PEO chains on the force balance

To understand in detail how the different PEO polymer chains used in this study affect the melting temperature, we conduct specific runs of the interaction model. The different PEO chains have different molecular weights $M_w$ but also different coating densities $\sigma$. To rationalize the effect of either change, we calculate the free energy of interaction for (a) varying coating densities but keeping $M_w$ constant (see Fig. S19-a) and (b) for varying $M_w$ but constant coating densities (see Fig. S19-b).

We find that increasing the density (albeit at constant $M_w$), from $\sigma_{34\text{k}} = 1/(6.9\text{ nm})^2$ to $\sigma_{11\text{k}} =$

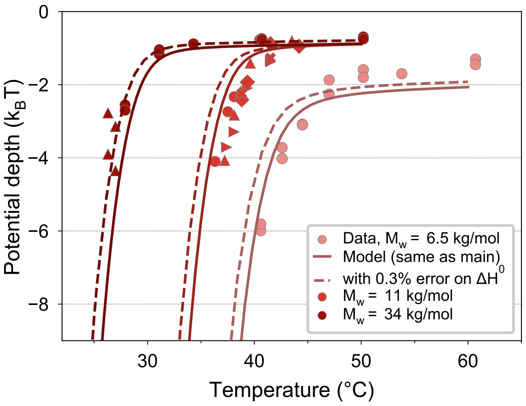


**Figure S17:** Effect of error on bare hybridization energy on the predictions of this model for the particles with $A^4/B^4$ used in this work. A −0.02 kcal/mol error per sticky nucleotide on $\Delta H^0$ was applied resulting in a 0.3% error on $\Delta H^0$ and a 1°C error on the melting temperature $T_m^{ab}$ of the bare sequence.

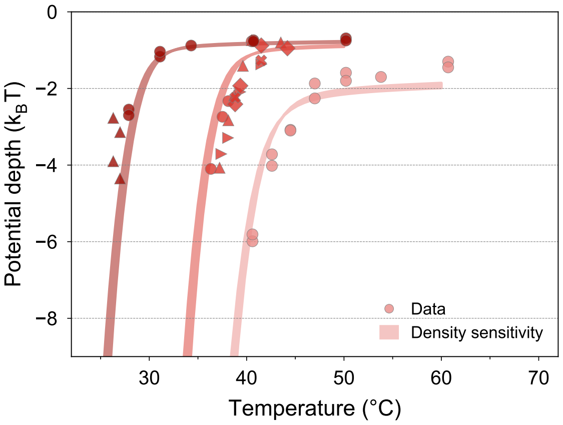


**Figure S18:** Effect of error on the calibration of the coating density on the bottom glass slide $\sigma_g$, on the predictions of this model for the particles with $A^4/B^4$ used in this work. Here we take into account a ±10% error range on $\sigma_g$.

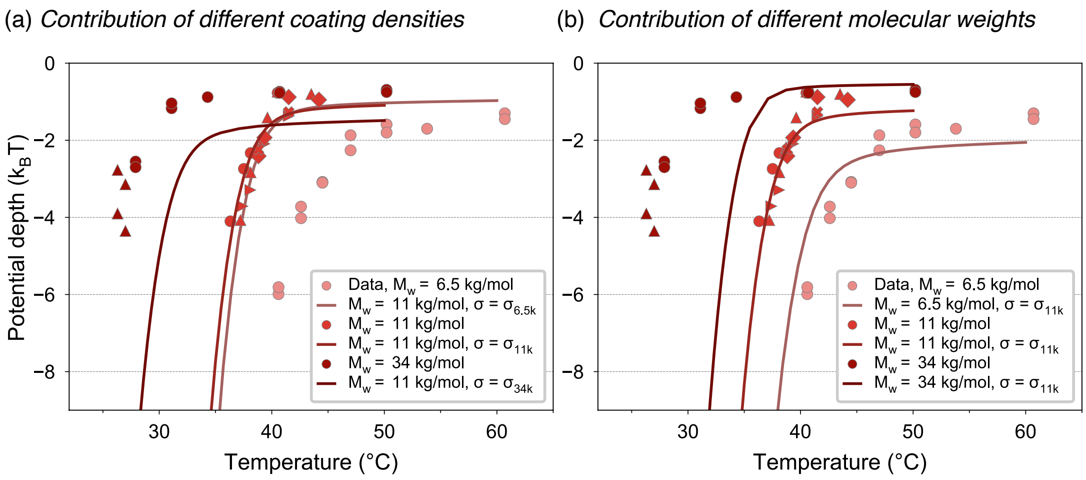


**Figure S19:** Model investigations to separate the different contributions of chain lengths, that both have specific molecular weight $M_w$ and associated coating density $\sigma$. (a) Effect of different densities, while keeping the molecular weight constant (equal to that of the intermediate length used, namely that of 11 kg/mol PEO). The effective excluded volume was also kept constant here, equal to that of 11 k. (b) Effect of different molecular weights, while keeping coating densities equal (to that of 11 k, $\sigma_{11k} = 1/(3.6\text{ nm})^2$. Model curves in (a) and (b) include shot noise. All investigations were conducted on the $A^4/B^4$ system of Fig. 3 of the main text.

$1/(3.6\text{ nm})^2$ to $\sigma_{6.5k} = 1/(3.27\text{ nm})^2$, systematically increases the melting temperature. This is not a trivial result. In fact, increasing the coating density increases both hybridization attraction (in absolute value) and steric repulsion. Therefore, a change in coating density may result in increased or decreased melting temperature *a priori*. However, the nonlinearities in the hybridization attraction $\varphi_{\text{bind}} \simeq \sigma f(\sigma)\Delta G_{ab}(\sigma)$ dominate compared to the mostly linear contributions in the steric repulsion $\varphi_{\text{steric}} \simeq \sigma$ (at least for the large coating densities investigated here). The increased densities thus result in increased melting temperature. Interestingly, such differences in the coating densities result in ~ 7°C range of melting temperatures, insufficient to account for the ~ 15°C range observed experimentally for this system.

We find that decreasing the molecular weight (albeit at constant density), from $M_w$ = 34, to 11 to 6.5 kg/mol results in increased melting temperature. This is quite easily understood. In fact, higher molecular weights correspond to a greater number of chain segments, and therefore increased steric re-

pulsion due to lost degrees of freedom during compression, because more chain segments are displaced. This effect accounts for the remaining $\sim 7°$C required to reproduce the experimental range of melting temperatures.

## 5.3 Analysis of modeling choices

### 5.3.1 Unified model versus "no brush modification"

We now evaluate the differences between our unified model, derived in Sec. 3.4, and a more "rapid" model that ignores modifications of the brush's elastic properties upon binding. This latter model with "no brush modification" assumes that the brushes structure and hence that the steric repulsion is not modified by the fraction of bound ends. This amounts to saying that $f = 0$ in the exclusion energy Eq. (S62) and in the stretching energy Eq. (S67). In essence, this simply means that the steric repulsion is given by the standard Milner-Witten-Cates theory. The fraction of bound ends can then simply be inferred by minimizing the binding energy $\partial\varphi_{\text{bind}}(h, f)/\partial f = 0$. In the symmetric case we obtain that the fraction of bound ends is then given by Eq. (S29), *e.g.* similarly as in Ref. 42. Note that the "no brush modification" model still contains the entropic contribution due to the loss of degrees of freedom upon binding.

As was mentioned before, we expect that in the "weak binding" regime, both models should not be too different. In fact, since just a few DNA sticky ends bind, the brush's elastic properties should not be too modified, and hence models should be equivalent. We remark, calculating binding and steric contributions for a representative system studied experimentally here, that both models give indeed indistinguishable results in the "weak binding" regime, *i.e.* around the melting point (see Fig. S20-a).

Reciprocally, in the "strong binding" regime, we expect discrepancies between the two models, as more DNA sticky ends bind and hence modify the brush's elastic properties. Comparing both models (Fig. S20-b) we indeed find small discrepancies. While the fraction of bound ends does not change significantly, the steric repulsion is underestimated by the "no brush modification" model.

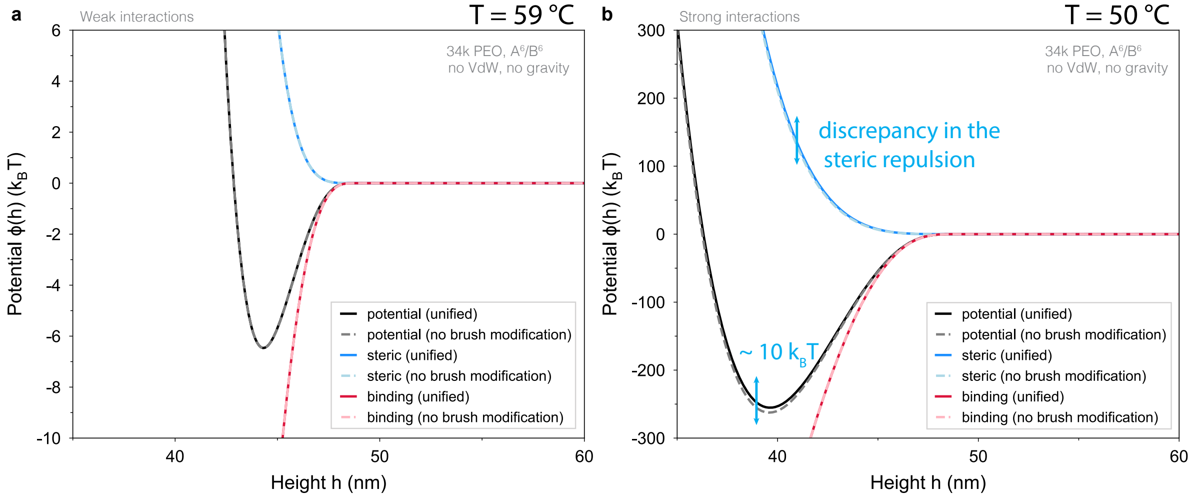


**Figure S20:** Comparison of the unified model of Sec. 3.4 with the model ignoring the modifications of the brush's elastic properties upon binding. We use a representative colloidal design with 34$k$ PEO and $A^6/B^6$ DNA sticky sequence (that of Figs. 1-2 of the main manuscript) at 2 representative temperatures $T = 59\ °C$ (a) and $T = 50\ °C$ (b).

### 5.3.2 Self-consistent approach versus "Poisson approximation"

We now assess how the self-consistent approach where we took into account the competition between binding partners (Eq. (S27)) versus the "Poisson approximation" Eq. (S24) that basically treats all

strands as indistinguishable, and in essence independent (they don't compete for partners). This approximation assumes that, given a certain temperature, the distribution of bonds $p(N)$ is distributed with a Poisson distribution

$$p(N) = \frac{(\langle N_{\text{bound}} \rangle)^N}{N!} e^{-\langle N_{\text{bound}} \rangle} \tag{S109}$$

where $\langle N_{\text{bound}} \rangle$ is the average number of bonds. Here we take it to be given by the full theory (with competition for binding partners) hence by Eq. (S84), and our aim is to check whether the "Poisson approximation" is consistently observed in our model, or not.

In the full theory, we can simply derive $p(N)$ since $p(N)dN = p(h)dh$ hence

$$p(N) = p(h)\frac{dN}{dh} = e^{-\beta\varphi(h)}\frac{dN_{\text{bound}}}{dh} \tag{S110}$$

where $N_{\text{bound}}(h)$ is defined by Eq. (S83). The binding strength is then given by $\beta\phi_{\text{bind,Poisson}}(h) = -N_{\text{bound}}(h)$ (see *e.g.* Ref. 1).

We compare the distributions in Fig. S21-a and c. As expected, the Poisson distribution is quite different compared to the full distribution obtained accounting for competition between binding partners, especially when just a few bonds are formed in average (Fig. S21-a). At lower temperatures, when a large number of bonds are in contact the discrepancies between both distributions are reduced (Fig. S21-c). At this stage, the law of large numbers brings both distributions towards Gaussian distributions, since also the number of accessible strands increases drastically.

The binding potential using the Poisson approximation is then compared to that predicted with the full model. Assuming the steric repulsion is the same in both cases, we can obtain the difference in the predicted potential well depth (Fig. S21-b and d). Around the melting temperature for a characteristic colloidal design, we already find a $\sim 1\ k_BT$ difference, accumulating to much larger discrepancies at lower temperatures. Hence, the "Poisson approximation" is clearly not satisfied in our systems and competition between binding partners must be taken into account.

# 6 Correspondence of microscopic model potential profiles to standard interaction profiles

The potential profile for the colloid, obtained as $\phi(h) = \phi_{\text{binding}}(h) + \phi_{\text{steric}}(h)$ calculated with the detailed DNA-DNA interactions as described above, is now compared to typical interaction profiles (Morse, Lennard-Jones and Wang-Frenkel[72]). We investigate which macroscopic interaction profile may represent the most accurately the full potential of interaction containing the microscopic details $\phi(h)$. Our constraint will be that each effective potential should capture the main thermodynamic features[38] of the simulated potential $\phi(h)$, namely the location of the minimum $h_{\text{min}}$, the depth of the minimum $\phi(h_{\text{min}})$ and the potential width (or curvature radius) given by $R_{\text{min}} = (\phi''(h_{\text{min}}))^{-1/2}$.

Please note, that in this Sec. 6, and in this section alone, we will use $\sigma$ to refer not to surface coating densities but to its usual meaning within the study of standard potentials, *i.e. to the range of the standard potential (such as that for Lennard-Jones, traditionally described through the* $(\epsilon, \sigma)$ *parameters).*

The standard potentials that we investigate are:

- the Morse potential

$$\phi_{\text{Morse}}(h, \sigma, r_e, \epsilon) = \epsilon\left(e^{-2(h-r_e)/\sigma} - 2e^{-(h-r_e)/\sigma}\right). \tag{S111}$$

  Here $\epsilon = -\phi(h_{\text{min}})$, $r_e = h_{\text{min}}$, and $\sigma = R_{\text{min}}\sqrt{2\epsilon}$.

- the truncated and shifted Lennard-Jones (LJ) potential (set at distance $r_e$ from the surface of the

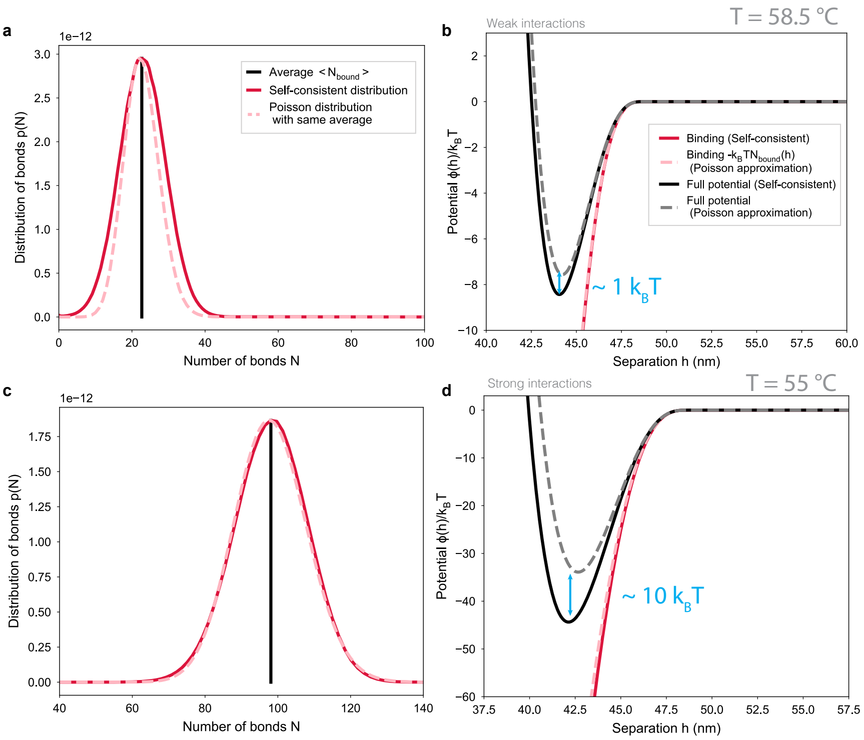


**Figure S21:** Comparison of the Self-consistent (our "Unified" model) and the Poisson approximation for a representative colloidal design with 34$k$ PEO and $A^6/B^6$ DNA sticky sequence (that of Figs. 1-2 of the main manuscript) at 2 representative temperatures $T = 58.5\ ^\circ C$ (a,b) and $T = 55\ ^\circ C$ (c,d). (a,c) Distribution of bound ends at each temperature using Eq. (S110) (self-consistent) or Eq. (S109) ("Poisson approximation"). Both distributions are set such that their maximum coincides (they are not normalized and presented in arbitrary units). Note that the horizontal axis has a different starting point between (a) and (c) but the scale is the same. (b) Potential profiles showing the binding part and the full potential (with binding and steric repulsion). $N_{\text{bound}}(h)$ is calculated via Eq. (S83) and $\langle N_{\text{bound}}\rangle$ via Eq. (S84). *Here the "Poisson approximation" underestimates the actual potential, which is different from Ref. 43 (which predicts an overestimate). In fact, here we do not use the "Poisson approximation" to estimate the number of bonds as in Ref. 43, rather, we estimate the "Poisson" interaction based on the number of bonds calculated using the self-consistent potential.*

colloid, since the repulsive interaction happens slightly beyond the colloid radius)

$$\phi_{\text{LJ}}(h, \sigma, r_e, \epsilon, m) = \begin{cases} \phi_{\text{LJ},0}(h, \sigma, r_e, \epsilon, m) - \phi_{\text{LJ},0}(r_e + 2.5\sigma, \sigma, r_e, \epsilon, m) & \text{for } r \le c \\ 0 \ \text{ for } r \ge 2.5\sigma \end{cases}$$
$$\phi_{\text{LJ},0}(h, \sigma, r_e, \epsilon, m) = 4\epsilon\left[\left(\frac{\sigma}{(h - r_e)}\right)^{2m} - \left(\frac{\sigma}{(h - r_e)}\right)^{m}\right]. \tag{S112}$$

The most common LJ potential has $m = 6$ which we refer to in the following simply as LJ potential. In that case, simply $\epsilon = -\phi(h_{\min})$, $\sigma = R_{\min}\sqrt{36 \times 2^{2/3}\epsilon}$, and $r_e = h_{\min} - \sigma 2^{1/6}$.

Generalized versions include arbitrary values of $m$. In that case, there is an additional parameter for the LJ potential compared to the number of constraints, and we add an additional constraint, namely that the potential cutoff corresponds to the maximum extent of the interaction profile $2.5\sigma = h_{\max}$. We thus have $\epsilon = -\phi(h_{\min})$, $\sigma = R_{\min}\sqrt{m^2 \times 2^{(m-2)/m}\epsilon}$, $r_e = h_{\min} - \sigma 2^{1/m}$, and $2.5\sigma = h_{\max}$. We refer to this potential as the LJ-m potential.

Note that there are other ways to truncate the LJ potential[72], and although we do not report results here, an alternative truncation such as the common spline LJ potential[73] does not achieve better results.

- Finally we also investigate the newly introduced Wang-Frenkel (WF) potential[72]

$$\phi_{\mathrm{WF}}(h,\sigma,r_e,\epsilon) = \begin{cases} \epsilon\alpha(r_e)\left[\left(\frac{\sigma}{h}\right)^2 - 1\right]\left[\left(\frac{r_e}{h}\right)^2 - 1\right]^2 & \text{for } r \le r_e \\ 0 \ \text{ for } r \ge r_e \end{cases}$$
$$\text{with } \alpha(r_e) = 2\left(\frac{r_e}{\sigma}\right)^2\left(\frac{3}{2}\frac{1}{\left(\frac{r_e}{\sigma}\right)^2 - 1}\right)^3 . \tag{S113}$$

For the WF potential, $\epsilon = -\phi(h_{\mathrm{min}})$, $r_e = \frac{h_{\mathrm{min}}\sigma}{\sqrt{3\sigma^2 - 2h_{\mathrm{min}}^2}}$, and $\frac{1}{R_{\mathrm{min}}^2} = \frac{2\epsilon(2r_e^2+\sigma^2)^3}{r_e^2\sigma^2(r_e^2-\sigma^2)^2}$.

We investigate a representative detailed potential $\phi(h)$, for typical experimental parameters a few degrees below the melting temperature . We compare the detailed interactions calculation to the model potential as obtained, again, for each potential, by imposing that essential thermodynamic parameters are preserved ($h_{\mathrm{min}}$, $\phi(h_{\mathrm{min}})$ and $\phi''(h_{\mathrm{min}})$), and additionally the cutoff for the LJ-m potential. We show the results in Fig. S22. The model potential parameters $(\sigma, r_e, \epsilon, m)$ are reported in Table. S13 and their visual comparison to $\phi(h)$ in Fig. S22. We find that the Wang-Frenkel potential allows to capture the narrow features of the full interaction profile $\phi(h)$ much better than any of the other macroscopic profiles, and this without any fitting parameters. Other macroscopic profiles (such as Lennard Jones or Morse) feature long range broadening of the potential, or some degree of asymmetry, that is not a characteristic of the multivalent ligand-receptor interactions investigated here. The WF potential therefore appears as a promising macroscopic model to account for such interactions.

Note that although we do not show it here, the results are reproducible regardless of the working temperature.

| Model | "range" $\sigma$ [nm] | "minimum" $r_e$ [nm] | depth $\epsilon$ $[k_BT]$ | parameter $m$ |
|---|---|---|---|---|
| Morse | 2.3 | 43.1 | 18.5 | N.A. |
| LJ (m = 6) | 12.1 | 29.6 | 18.5 | 6 |
| LJ-m | 19 | 22 | 18.5 | 9 |
| WF | 41 | 48 | 18.5 | N.A. |
| $\phi(h)$ | $R_{\mathrm{min}} = 0.4$ | $h_{\mathrm{min}} = 43.1$ | $-\phi(h_{\mathrm{min}}) = 18.5$ | N.A. |

**Table S13:** Parameters of the model potentials (Morse, LJ and WF) used such that their thermodynamic parameters correspond exactly with that of potential $\phi(h)$ calculated from detailed interactions.

# 7 Agreement of model predictions with previous experimental measurements

In this section we compare our model with previously reported data on different but related systems.

## 7.1 Experiments from Rogers & Crocker[1]

**Modeling details and experimental parameters entering the prediction.** Pair potentials where also measured for an A/B type surface through particle-particle distances in Ref. 1 for low density coverages. Here, as the brushes are not dense, they are likely in a mushroom configuration. Therefore, steric repulsion and binding energy have to be derived for mushroom polymers. Briefly, we use the mean-field probability distribution profiles building on the work of Ref. 74 for polymer mushrooms tethered on

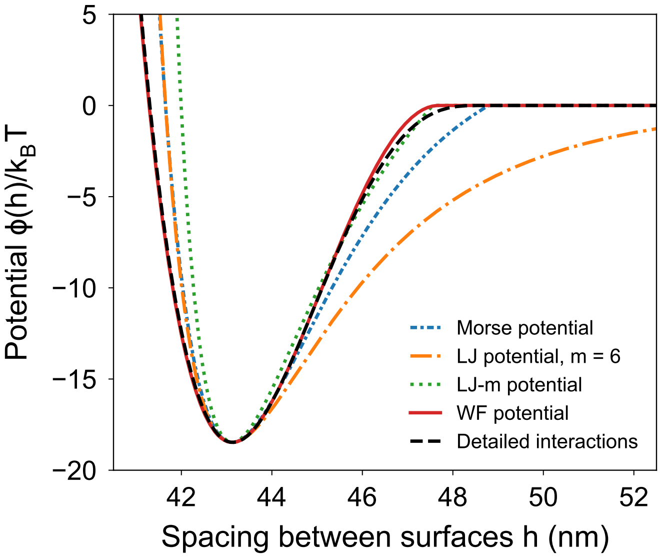


**Figure S22:** Model potential profiles with steric repulsion and binding attraction (without shot noise, without gravity and without van der Waals interactions) for a characteristic set of experimental parameters (dashed black). Here experimental parameters used are that of the experimental system of Fig. 1 and 2 of the main paper, namely a colloid with 34k PEO and 20 DNA nucleotides including 6 sticky ends, and density $1/(6.9\ \text{nm}^2)$; while the bottom layer is 60 DNA nucleotides including the 6 complementary ends, and density $1/(9.4\ \text{nm}^2)$. $T = 56.9°$ C. The simulated potential profile is compared to standard potential profiles. Standard potential profile parameters are set to match thermodynamic parameters of the interaction potential resulting from detailed microscopic account.

one end and compressed with a plate, that we extend to bridges. This allows us to find steric repulsion and entropic costs due to binding. Finally, we find the fraction of bound tethers self-consistently. Here we expect a "no brush modification" framework as the brushes are not dense enough that binding may affect the elastic properties of the tethered strands. We also divide the Dejarguin integral by a factor 2 to account for such colloid-colloid interactions (compared to colloid-plate interactions in our work).

The detailed parameters for the A/B colloids are used. Namely, the coating density of A particles is that measured experimentally $\sigma_A = 0.0013\ \text{nm}^{-2}$ (4800 DNA per particle) and of B is $\sigma_B = 0.0011\ \text{nm}^{-2}$ (4200 DNA per particle), the exact DNA ligand and sticky sequence is used, 1 PEO segment of the triblock F108 as linker on each side, and the particle radius $a = 550$ nm. The sticky sequence used allows us to infer bare hybridization energies according to the unified nearest neighbor model of SantaLucia[70] (see also Sec. 3.3.2). Here, to model the ssDNA strands we use a persistence length $\ell_{\text{DNA}} = 1.49$ nm (less than half the Kuhn length used in the model of Ref. 1, but in agreement with the recent measurements of persistence lengths for ssDNA at that salt concentration, 136 mM[29]). The total height of the brush is then given by $R$, with $R^2 = R^2_{\text{PEO}} + R^2_{\text{DNA}}$ where $R_{\text{PEO}}$ is given by Eq. (S3) and $R_{\text{DNA}} = \ell_{\text{DNA}}\sqrt{N_{\text{DNA}}}$ (and $N_{\text{DNA}} = N_0 b_0/\ell_{\text{DNA}}$, as is described in Sec. 2.4.2, with $N_0 = 65$ nucleotides). Note that van der Waals interactions are not included between the surfaces as surfaces in these experiments are passivated via a carboxyl layer.

To distort potentials due to measurement noise, we use, as in Ref. 1, a gaussian noise kernel of width 3 nm. This is an arbitrary choice for the noise-distorted potential – and the noise-distortion in such samples would have to be assessed in more details to understand the true effect of noise. Here we find that the gaussian noise kernel yields relatively comparable broad potentials as those measured and hence is a reasonable first approximation of noise distortion.

**Comparison between experiments and model predictions.** The interparticle potential depth is then plotted with respect to the temperature and compared to data found in Ref. 1 (data was extracted using WebPlotDigitizer[75]) in Fig. S23a.

With no fitting parameters, we find reasonable agreement between potential well depths data and

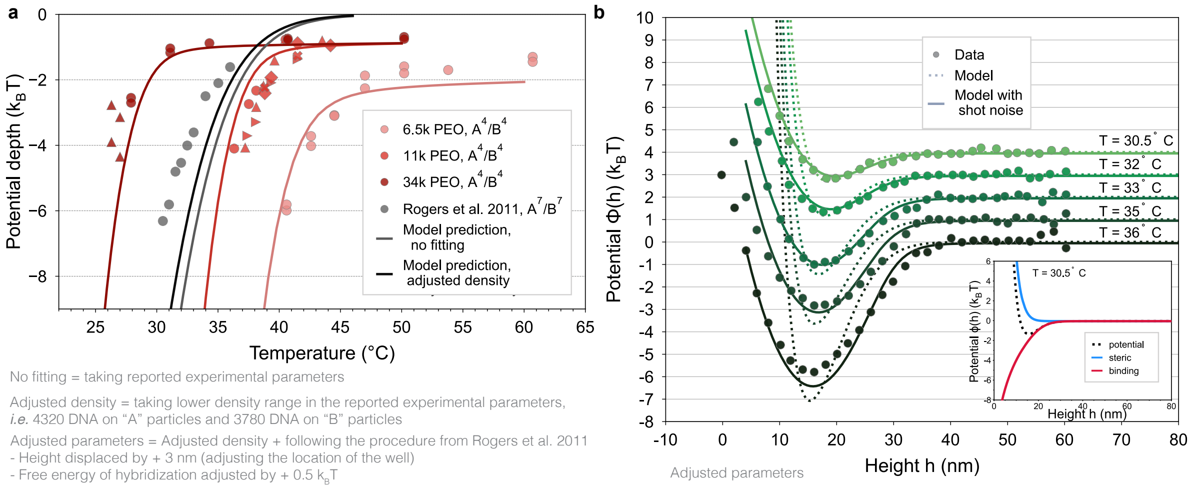


**Figure S23:** Comparison of our model with the experimental results of Ref. 1. (a) Potential well depths with measurements from Ref. 1 and model predictions, using the exact experimental parameters reported in Ref. 1 (see details in text), including gaussian noise (gray) and with adjusted density to the lowest coating density within experimental range (black). For comparison the measurements and model predictions on our $A^4/B^4$ coated colloids are reported here. (b) Pair potential measurements from Ref. 1 and model predictions with adjusted parameters within experimental error (coating density, height origin, free energy of hybridization – similarly as in the modelling efforts by Ref. 1, see text for details). Curves at 36°C are plotted at their exact potential value. Curves at higher temperatures are presented at $1k_BT$ apart for readability. (inset) Breakdown of contributions to the potential profile at high temperature.

prediction. In terms of the temperature corresponding to the melting transition, *i.e.* the temperature around which the well depth $\sim -3k_BT$, our prediction is within 2 °C of experimental data (Fig. S23a, gray). This corresponds to the typical experimental dispersion from one particle to another in our samples, hence the agreement can be considered already quite satisfactory. Furthermore, using an adjusted value of coating densities, similarly as we have done in our calibration procedure for the glass slide, we find the agreement can be improved to within 1 °C (Fig. S23b, gray). The adjusted density used corresponds to the lower experimental range, *i.e.* 10% lower (4320 DNA per type "A" particle and 3780 DNA per type "B" particle).

To further compare predictions and experimental results reported in Ref. 1 we investigate the potential profiles, and what corrections need to be used on our model to reproduce experimental data.

- *Adjustment of the height origin.* We notice that our potential wells feature a minimum at around $\sim$ 12 nm interparticle separation $h$. In experiments, the minimum arises further, around $\sim$ 15 nm. In the experiments in Ref. 1, the interparticle separation is obtained from the distance between the 2 particles center of mass. Hence, the exact particle size enters the determination of the interparticle separation, yet it does not appear to be known with a nm precision. Furthermore, the particle is coated with a carboxyl layer that is not characterized. We therefore expect uncertainties on the height origin as reported in Ref. 1. As a consequence, it is reasonable to allow for adjustment of the height origin, and in Fig. S23-b we plot pair-potential predictions with a +3 nm correction on the height origin. Note, that in the modeling efforts in Ref. 1, fitting of the height origin is performed.

The errors on the height origin are tied to the general issue that the brush characteristics in Ref. 1 are not measured. Hence, we do not know the exact thickness of the brush, in particular not of the carboxyl sub-layer. Yet, knowledge of brush characteristics is essential to accurately predict the steric repulsion associated with brush compression. Hence, it is not surprising that our predictions are 1 – 2°C off from experimental measurements. Further, we may expect that the carboxyl sub-layer increases the effective size of the brush, therefore increasing steric repulsion and hence would reduce the melting temperature: which would indeed improve agreement with data (see also Fig. S19-b where the effect of brush length increase was investigated).

- Adjustment of the bare free energy of hybridization. Alternatively, to account for well depth discrepancies, we can, similarly as in Ref. 1, adjust the bare free energy of hybridization $\Delta G^0_{ab}$ within experimental error (see Sec. 5.2.1). We use a +0.04 kcal/mol correction per sticky nucleotide on $\Delta G^0_{ab}$, which is well within the expected experimental error range, and obtain perfect agreement between experimental data and model predictions (see Fig. S23-b); suggesting that the elements of the model are sufficient to reproduce experimental data.

**Modeling differences with Ref. 1.** Finally, we discuss differences between the modeling approach in Ref. 1 and our own. While detailed differences are discussed at length in Sec. 3.3, here we explore one major difference, namely that we account for competition between binding partners while Ref. 1 neglects such competition, assuming strands are indistinguishable, and hence uses the "Poisson approximation" to obtain the binding free energy. Similarly as in Sec. 5.3.2 we compare the bond distribution and predicted potential profile with the 2 methods and find significant differences between modeling approaches (see Fig. S24). First, since the average number of bonds is much smaller than in our colloidal designs due to a lower coating density, we find that the distribution of bonds greatly differs from a Poisson distribution (Fig. S24-b). Hence the "Poisson approximation" in Ref. 1 is inaccurate. It notably predicts a 40% error on the potential depth for a $\sim 10k_BT$ potential depth (Fig. S24-b).

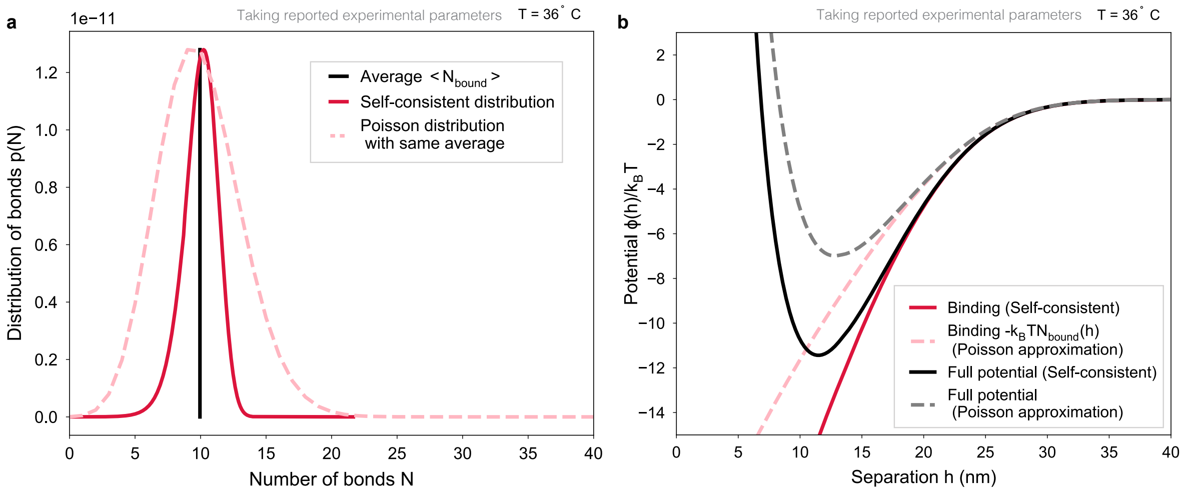


**Figure S24:** Comparison of the Self-consistent (our "Unified" model) and the Poisson approximations for the data of Ref. 1 at a representative temperature $T = 36\ °C$. Plot similar to Fig. S20. (a) Distribution of bound ends at that temperature and (b) Potential profiles showing the binding part and the full potential (with binding and steric repulsion). $N_{\text{bound}}(h)$ is calculated via Eq. (S83) and $\langle N_{\text{bound}} \rangle$ via Eq. (S84).

Overall this confirms that our mean-field model is well suited to account for DNA-coated colloids interactions even in less dense settings where brush properties are still rather uniform.

## 7.2 Experiments from Xu *et al.*[2]

We also investigate the reliability of our model on systems made with double-stranded DNA such as that of Ref. 2. We use our mean-field model with a polymer brush described by Milner-Witten-Cates, as in the main paper. In fact the systems in Ref. 2 are quite densely coated, and also feature adsorbed F108 surfactant in between sticky brushes. Instead of the PEO linker, We use here the parameters of the double-stranded DNA part (number of nucleotides, persistence length). The exact sequence of the sticky single stranded part is used. Coating densities of $1/(12\text{ nm})^2$ and $1/(18\text{ nm})^2$ for the particle and the surface where used as reported in Ref. 2. The effect of F108 adsorption on brushes is modelled exactly as F127 adsorption in our work. This is a reasonable assumption as both triblock copolymers are very similar.

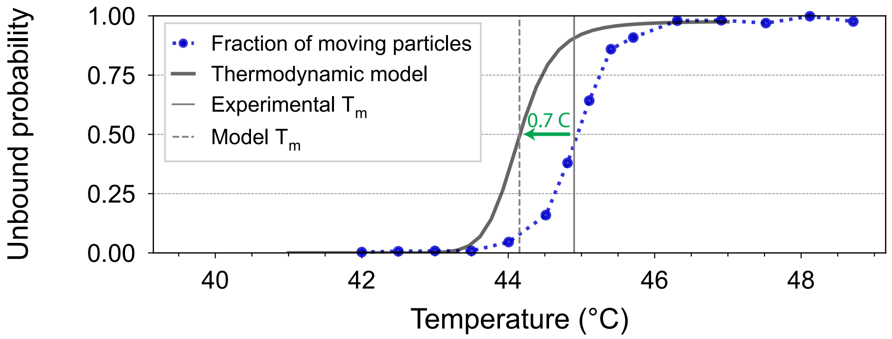


**Figure S25:** Comparison of our model with the experimental results of Ref. 2. Thermodynamic melting curve as predicted by our mean-field model, with no fitting parameters (see details in the text). Experimental data is from Ref. 2.

We obtain a thermodynamic melting curve, in Fig. S25 that we compare to the kinetic-like melting curve reported in Ref. 2 (data was extracted using WebPlotDigitizer[75]). We find a very good agreement with a discrepancy of only 0.7°C between the predicted melting temperature and the reported one, with absolutely no fitting parameters. This is quite remarkable considering that the brush was not thoroughly characterized, especially the effect of F108 adsorption on brush extension. Furthermore, as the definition of melting in Ref. 2 corresponds to a different kinetic-like definition, based on lateral motion of particles, it is highly dependent on kinetic features such as the observation time. Therefore we attribute the remaining temperature discrepancy to kinetic features that are not accounted for in our purely thermodynamic, universal description.

Overall this confirms that our mean-field model is suited to account for DNA-coated colloids interactions with other type of linkers such as double-stranded DNA.

## Supplementary References


[1] Rogers, W. B. & Crocker, J. C. Direct measurements of DNA-mediated colloidal interactions and their quantitative modeling. *Proceedings of the National Academy of Sciences* **108**, 15687–15692 (2011).

[2] Xu, Q., Feng, L., Sha, R., Seeman, N. & Chaikin, P. Subdiffusion of a sticky particle on a surface. *Physical Review Letters* **106**, 228102 (2011).

[3] National Instruments. *X Series User Manual* (2019).

[4] Helden, L., Eremina, E., Riefler, N., Hertlein, C., Bechinger, C., Eremin, Y. & Wriedt, T. Single-particle evanescent light scattering simulations for total internal reflection microscopy. *Applied optics* **45**, 7299–7308 (2006).

[5] Prieve, D. C. Measurement of colloidal forces with TIRM. *Advances in Colloid and Interface Science* **82**, 93–125 (1999).

[6] McKee, C. T., Clark, S. C., Walz, J. Y. & Ducker, W. A. Relationship between scattered intensity and separation for particles in an evanescent field. *Langmuir* **21**, 5783–5789 (2005).

[7] Li, J.-T., Caldwell, K. D. & Rapoport, N. Surface properties of pluronic-coated polymeric colloids. *Langmuir* **10**, 4475–4482 (1994).

[8] Vos, R., Rolin, C., Rip, J., Conard, T., Steylaerts, T., Cabanilles, M. V., Levrie, K., Jans, K. & Stakenborg, T. Chemical vapor deposition of azidoalkylsilane monolayer films. *Langmuir* **34**, 1400–1409 (2018).

[9] Nejadnik, M. R., Olsson, A. L., Sharma, P. K., van der Mei, H. C., Norde, W. & Busscher, H. J. Adsorption of pluronic F-127 on surfaces with different hydrophobicities probed by quartz crystal microbalance with dissipation. *Langmuir* **25**, 6245–6249 (2009).

[10] Valignat, M.-P., Theodoly, O., Crocker, J. C., Russel, W. B. & Chaikin, P. M. Reversible self-assembly and directed assembly of DNA-linked micrometer-sized colloids. *Proceedings of the National Academy of Sciences* **102**, 4225–4229 (2005).

[11] Wang, Y., Wang, Y., Zheng, X., Ducrot, É., Yodh, J. S., Weck, M. & Pine, D. J. Crystallization of DNA-coated colloids. *Nature Communications* **6**, 7253 (2015).

[12] Youssef, M., Morin, A., Aubret, A., Sacanna, S. & Palacci, J. Rapid characterization of neutral polymer brush with a conventional zetameter and a variable pinch of salt. *Soft Matter* (2020).

[13] Hiki, S. & Kataoka, K. A facile synthesis of azido-terminated heterobifunctional poly(ethylene glycol)s for "click" conjugation. *Bioconjugate Chemistry* **18**, 2191–2196 (2007).

[14] Oh, J. S., Wang, Y., Pine, D. J. & Yi, G.-R. High-density PEO-b-DNA brushes on polymer particles for colloidal superstructures. *Chemistry of Materials* **27**, 8337–8344 (2015).

[15] Ohshima, H. Modified Henry function for the electrophoretic mobility of a charged spherical colloidal particle covered with an ion-penetrable uncharged polymer layer. *Journal of Colloid and Interface Science* **252**, 119–125 (2002).

[16] He, M., Gales, J. P., Ducrot, É., Gong, Z., Yi, G.-R., Sacanna, S. & Pine, D. J. Colloidal diamond. *Nature* **585**, 524–529 (2020).

[17] Baker, J. A. & Berg, J. C. Investigation of the adsorption configuration of polyethylene oxide and its copolymers with polypropylene oxide on model polystyrene latex dispersions. *Langmuir* **4**, 1055–1061 (1988).

[18] Agard, N. J., Prescher, J. A. & Bertozzi, C. R. A strain-promoted [3+ 2] azide- alkyne cycloaddition for covalent modification of biomolecules in living systems. *Journal of the American Chemical Society* **126**, 15046–15047 (2004).

[19] Rubinstein, M. & Colby, R. H. *Polymer Physics* (Oxford University Press, Oxford, 2003).

[20] Mark, J. & Flory, P. The configuration of the polyoxyethylene chain. *Journal of the American Chemical Society* **87**, 1415–1423 (1965).

[21] Oelmeier, S. A., Dismer, F. & Hubbuch, J. Molecular dynamics simulations on aqueous two-phase systems-single peg-molecules in solution. *BMC biophysics* **5**, 14 (2012).

[22] Milner, S. T. Compressing polymer "brushes": a quantitative comparison of theory and experiment. *EPL (Europhysics Letters)* **7**, 695 (1988).

[23] Milner, S. T., Witten, T. & Cates, M. A parabolic density profile for grafted polymers. *EPL (Europhysics Letters)* **5**, 413 (1988).

[24] Milner, S. T., Witten, T. & Cates, M. Theory of the grafted polymer brush. *Macromolecules* **21**, 2610–2619 (1988).

[25] Money, N. P. Osmotic pressure of aqueous polyethylene glycols: relationship between molecular weight and vapor pressure deficit. *Plant Physiology* **91**, 766–769 (1989).

[26] Steuter, A. A., Mozafar, A. & Goodin, J. R. Water potential of aqueous polyethylene glycol. *Plant Physiology* **67**, 64–67 (1981).

[27] Stanley, C. B. & Strey, H. H. Measuring osmotic pressure of poly (ethylene glycol) solutions by sedimentation equilibrium ultracentrifugation. *Macromolecules* **36**, 6888–6893 (2003).

[28] Wu, T., Efimenko, K., Vlcek, P., Šubr, V. & Genzer, J. Formation and properties of anchored polymers with a gradual variation of grafting densities on flat substrates. *Macromolecules* **36**, 2448–2453 (2003).

[29] Chen, H., Meisburger, S. P., Pabit, S. A., Sutton, J. L., Webb, W. W. & Pollack, L. Ionic strength-dependent persistence lengths of single-stranded RNA and DNA. *Proceedings of the National Academy of Sciences* **109**, 799–804 (2012).

[30] Murphy, M., Rasnik, I., Cheng, W., Lohman, T. M. & Ha, T. Probing single-stranded DNA conformational flexibility using fluorescence spectroscopy. *Biophysical Journal* **86**, 2530–2537 (2004).

[31] Kenworthy, A. K., Hristova, K., Needham, D. & McIntosh, T. J. Range and magnitude of the steric pressure between bilayers containing phospholipids with covalently attached poly (ethylene glycol). *Biophysical Journal* **68**, 1921–1936 (1995).

[32] Ferreira, P. & Leibler, L. Copolymer brushes. *The Journal of Chemical Physics* **105**, 9362–9370 (1996).

[33] Zakin, J. L., Simha, R. & Hershey, H. C. Low-temperature thermal expansivities of polyethylene, polypropylene, mixtures of polyethylene and polypropylene, and polystyrene. *Journal of Applied Polymer Science* **10**, 1455–1473 (1966).

[34] Derjaguin, B. V. Untersuchungen über die reibung und adhäsion, iv. *Kolloid-Zeitschrift* **69**, 155–164 (1934).

[35] Dahal, U., Wang, Z. & Dormidontova, E. E. Hydration of spherical peo-grafted gold nanoparticles: curvature and grafting density effect. *Macromolecules* **51**, 5950–5961 (2018).

[36] Brenner, H. The slow motion of a sphere through a viscous fluid towards a plane surface. *Chemical Engineering Science* **16**, 242–251 (1961).

[37] Bevan, M. A. & Prieve, D. C. Hindered diffusion of colloidal particles very near to a wall: Revisited. *The Journal of Chemical Physics* **113**, 1228–1236 (2000).

[38] Gardiner, C. W. *et al. Handbook of Stochastic Methods*, vol. 4th edition, chap. 5 (Springer Berlin, 1985).

[39] Mani, M., Gopinath, A. & Mahadevan, L. How Things Get Stuck: Kinetics, Elastohydrodynamics, and Soft Adhesion. *Physical Review Letters* **108**, 226104–5 (2012).

[40] Hensley, A., Jacobs, W. M. & Rogers, W. B. Self-assembly of photonic crystals by controlling the nucleation and growth of DNA-coated colloids. *Proceedings of the National Academy of Sciences* **119**, e2114050118 (2022).

[41] Lee-Thorp, J. P. & Holmes-Cerfon, M. Modeling the relative dynamics of DNA-coated colloids. *Soft Matter* **14**, 8147–8159 (2018).

[42] Varilly, P., Angioletti-Uberti, S., Mognetti, B. M. & Frenkel, D. A general theory of DNA-mediated and other valence-limited colloidal interactions. *The Journal of Chemical Physics* **137**, 094108 (2012).

[43] Angioletti-Uberti, S., Varilly, P., Mognetti, B. M., Tkachenko, A. V. & Frenkel, D. Communication: A simple analytical formula for the free energy of ligand-receptor-mediated interactions (2013).

[44] Angioletti-Uberti, S., Mognetti, B. M. & Frenkel, D. Theory and simulation of DNA-coated colloids: a guide for rational design. *Physical Chemistry Chemical Physics* **18**, 6373–6393 (2016).

[45] Rogers, W. B. & Crocker, J. C. Reply to Mognetti et al.: DNA handshaking interaction data are well described by mean-field and molecular models. *Proceedings of the National Academy of Sciences* **109**, E380–E380 (2012).

[46] Mognetti, B. M., Varilly, P., Angioletti-Uberti, S., Martinez-Veracoechea, F. J., Dobnikar, J., Leunissen, M. E. & Frenkel, D. Predicting DNA-mediated colloidal pair interactions. *Proceedings of the National Academy of Sciences* **109**, E378–E379 (2012).

[47] Dreyfus, R., Leunissen, M. E., Sha, R., Tkachenko, A., Seeman, N. C., Pine, D. J. & Chaikin, P. M. Aggregation-disaggregation transition of DNA-coated colloids: Experiments and theory. *Physical Review E* **81**, 041404 (2010).

[48] Biancaniello, P. L., Kim, A. J. & Crocker, J. C. Colloidal interactions and self-assembly using DNA hybridization. *Physical Review Letters* **94**, 058302 (2005).

[49] Dreyfus, R., Leunissen, M. E., Sha, R., Tkachenko, A. V., Seeman, N. C., Pine, D. J. & Chaikin, P. M. Simple quantitative model for the reversible association of DNA coated colloids. *Physical Review Letters* **102**, 048301 (2009).

[50] SantaLucia, J. A unified view of polymer, dumbbell, and oligonucleotide DNA nearest-neighbor thermodynamics. *Proceedings of the National Academy of Sciences* **95**, 1460–1465 (1998).

[51] Markham, N. R. & Zuker, M. Dinamelt web server for nucleic acid melting prediction. *Nucleic acids research* **33**, W577–W581 (2005).

[52] Binder, K. & Milchev, A. Polymer brushes on flat and curved surfaces: How computer simulations can help to test theories and to interpret experiments. *Journal of Polymer Science Part B: Polymer Physics* **50**, 1515–1555 (2012).

[53] Meng, X.-X. & Russel, W. B. Telechelic associative polymers: Interactions between strongly stretched planar adsorbed layers. *Macromolecules* **36**, 10112–10119 (2003).

[54] Watanabe, H. & Tirrell, M. Measurement of forces in symmetric and asymmetric interactions between diblock copolymer layers adsorbed on mica. *Macromolecules* **26**, 6455–6466 (1993).

[55] Shim, D. & Cates, M. Forces between asymmetric polymer brushes. *Journal de Physique* **51**, 701–707 (1990).

[56] Hamaker, H. C. The London-van der Waals attraction between spherical particles. *Physica* **4**, 1058–1072 (1937).

[57] Israelachvili, J. N. *Intermolecular and surface forces* (Academic press, 2015).

[58] Parsegian, V. A. *Van der Waals forces: A handbook for biologists, chemists, engineers, and physicists* (Cambridge University Press, 2005).

[59] Bevan, M. A. & Prieve, D. C. Direct measurement of retarded van der waals attraction. *Langmuir* **15**, 7925–7936 (1999).

[60] Rudhardt, D., Bechinger, C. & Leiderer, P. Direct measurement of depletion potentials in mixtures of colloids and nonionic polymers. *Physical Review Letters* **81**, 1330 (1998).

[61] Lifshitz, E. M., Hamermesh, M. *et al.* The theory of molecular attractive forces between solids. In *Perspectives in Theoretical Physics*, 329–349 (Elsevier, 1992).

[62] Pedregosa, F. *et al.* Scikit-learn: Machine learning in Python. *Journal of Machine Learning Research* **12**, 2825–2830 (2011).

[63] Fernandez, D., Goodwin, A., Lemmon, E. W., Levelt Sengers, J. & Williams, R. A formulation for the static permittivity of water and steam at temperatures from 238 k to 873 k at pressures up to 1200 mpa, including derivatives and Debye-Hückel coefficients. *Journal of Physical and Chemical Reference Data* **26**, 1125–1166 (1997).

[64] Andelman, D. Electrostatic properties of membranes: the Poisson-Boltzmann theory. In *Handbook of Biological Physics*, vol. 1, 603–642 (Elsevier, 1995).

[65] von Grünberg, H.-H., Helden, L., Leiderer, P. & Bechinger, C. Measurement of surface charge densities on Brownian particles using total internal reflection microscopy. *The Journal of Chemical Physics* **114**, 10094–10104 (2001).

[66] Verwey, E. J. W., Overbeek, J. T. G. & Van Nes, K. *Theory of the stability of lyophobic colloids: The interaction of sol particles having an electric double layer* (Elsevier Publishing Company, 1948).

[67] Behrens, S. H. & Grier, D. G. The charge of glass and silica surfaces. *The Journal of Chemical Physics* **115**, 6716–6721 (2001).

[68] Cui, F. & Pine, D. J. Effect of photon counting shot noise on total internal reflection microscopy. *Soft Matter* **18**, 162–171 (2022).

[69] Gehrels, E. W., Rogers, W. B. & Manoharan, V. N. Using DNA strand displacement to control interactions in DNA-grafted colloids. *Soft Matter* **14**, 969–984 (2018).

[70] SantaLucia Jr, J. & Turner, D. H. Measuring the thermodynamics of RNA secondary structure formation. *Biopolymers: Original Research on Biomolecules* **44**, 309–319 (1997).

[71] SantaLucia Jr, J. & Hicks, D. The thermodynamics of DNA structural motifs. *Annu. Rev. Biophys. Biomol. Struct.* **33**, 415–440 (2004).

[72] Wang, X., Ramírez-Hinestrosa, S., Dobnikar, J. & Frenkel, D. The lennard-jones potential: when (not) to use it. *Physical Chemistry Chemical Physics* **22**, 10624–10633 (2020).

[73] Hafskjold, B., Travis, K. P., Hass, A. B., Hammer, M., Aasen, A. & Wilhelmsen, Ø. Thermodynamic properties of the 3d lennard-jones/spline model. *Molecular Physics* **117**, 3754–3769 (2019).

[74] Dolan, A. & Edwards, S. F. Theory of the stabilization of colloids by adsorbed polymer. *Proceedings of the Royal Society of London. A. Mathematical and Physical Sciences* **337**, 509–516 (1974).

[75] Rohatgi, A. WebPlotDigitizer: Version 4.5 (2021). URL https://automeris.io/WebPlotDigitizer.
